# A new *Cataleptoneta* species (Araneae, Leptonetidae) from sulfidic caves in Greece and Albania

**DOI:** 10.3897/zookeys.1294.202010

**Published:** 2026-10-06

**Authors:** István Urák, Andrei Ştefan, Rozalia Magda Motoc, Traian Brad, Andreea-Rebeka Zsigmond, Serban M. Sarbu, Tamás Szűts

**Affiliations:** 1 Department of Life Sciences, Sapientia Hungarian University of Transylvania, 520036 Sfântu Gheorghe, Ciucului str. 50, Cluj-Napoca, Romania Department of Life Sciences, Sapientia Hungarian University of Transylvania Cluj-Napoca Romania; 2 “Grigore Antipa” National Museum of Natural History, Şos. Kiseleff 1, 011341 Bucharest, Romania “Grigore Antipa” National Museum of Natural History Bucharest Romania; 3 Romanian Academy Cluj-Napoca Branch, Emil Racoviţă Institute of Speleology, Clinicilor str. 5-7, 400006 Cluj-Napoca, Bucharest, Romania Romanian Academy Cluj-Napoca Branch, Emil Racoviţă Institute of Speleology Cluj-Napoca Romania; 4 Department of Environmental Science, Sapientia Hungarian University of Transylvania, 400193 Cluj-Napoca, Calea Turzii str. 4, Romania Department of Environmental Science, Sapientia Hungarian University of Transylvania Cluj-Napoca Romania; 5 Emil Racoviţă Institute of Speleology, Calea 13 Septembrie str. 13, 050711 Bucharest, Romania Emil Racoviţă Institute of Speleology Bucharest Romania; 6 Department of Biological Sciences, California State University, Chico, CA, USA Department of Biological Sciences, California State University Chico United States of America; 7 Diversity and Evolution Research Group, Department of Zoology, University of Veterinary Medicine Budapest, Budapest, Hungary Diversity and Evolution Research Group, Department of Zoology, University of Veterinary Medicine Budapest Budapest Hungary

**Keywords:** Description, integrative taxonomy, scanning electron micrographs, Vromoner Canyon

## Abstract

A new species of the genus *Cataleptoneta* Denis, 1955, *Cataleptoneta sulfur***sp. nov**., is described from caves in the Vromoner Canyon (Sarandaporo River Valley), on the Greek–Albanian border (northwestern Greece and southern Albania), on the basis of both sexes. Drawings, scanning electron micrographs, and digital images of diagnostic features are presented. Analysis of COI mitochondrial barcode sequences confirmed the species distinction from all the other sequences. Within the species two separate lineages, unique to each cave were found. The genus *Cataleptoneta* is not monophyletic as now circumscribed and confirmed also by the phylogenetic analysis of the sequences.

## Introduction

Midget cave spiders (Araneae, family Leptonetidae Simon, 1890) are small (1.0–3.0 mm) spiders that are primarily adapted to life in cryptic habitats such as caves, leaf litter, soil crevices, and the undersides of stones (Ledford et al. 2011; Mammola and Isaia 2017; Wang et al. 2017, 2020; Liu et al. 2024; Bellvert et al. 2026). Some species exhibit clear troglomorphic traits, including elongated appendages, reduced pigmentation, and eye size, reflecting long-term isolation in subterranean environments (Wang et al. 2020; Bellvert et al. 2026). Leptonetids can be readily distinguished from other families by a characteristic 6-eyed pattern: four eyes form a compact group, with the remaining two situated posterior to them (Gertsch 1974; Le Peru 2011). In some troglophilic species, the eyes are strongly reduced to vestigial structures or may be entirely absent (Ledford et al. 2011; Bellvert et al. 2026).

Their distribution is notably fragmented, with centers of diversity in the Mediterranean region (Brignoli 1979), East Asia (Song et al. 1999), and North America (Gertsch 1974; Ledford et al. 2011), a pattern frequently discussed in the context of vicariance, often a result of insufficient data rather than a clear pattern, which is called the Wallacean shortfall (Lomolino 2004; Hortal et al. 2015). Despite the data insufficiency, leptonetids are of particular interest due to their limited dispersal ability and high levels of local endemism, especially among species inhabiting caves and other stable microhabitats (Gertsch 1974; Brignoli 1979; Ledford et al. 2011; Mammola and Isaia 2017). These characteristics make the family a valuable model for studying speciation processes driven by long-term isolation and microhabitat stability. Many cave leptonetid species exhibit low activity levels, traits that are consistent with ecological theories of energy optimization in resource-poor environments, as originally emphasized in classical biospeleological concepts (Racovitza 1907; Culver and Pipan 2009; Mammola and Isaia 2017; Bellvert et al. 2026). Although leptonetids are rarely encountered outside targeted surveys, they constitute an important yet often overlooked component of subterranean arthropod communities (Ledford et al. 2011).

The family Leptonetidae comprises 22 genera with 400 species worldwide (WSC 2026). In Europe 76 species were recorded, belonging to eight genera: *Barusia* Kratochvíl, 1978 (5 species), *Cataleptoneta* Denis, 1955 (7 species), *Leptoneta* Simon, 1872 (35 species), *Leptonetela* Kratochvíl, 1978 (12 species), *Paraleptoneta* Fage, 1913 (2 species), *Protoleptoneta* Deltshev, 1972 (4 species), *Sulcia* Kratochvíl, 1938 (10 species) and *Teloleptoneta* Ribera, 1988 (1 species) (Nentwig et al. 2026). Taxonomic delineation within Leptonetidae is particularly difficult due to their small body size, simplified female genital structures and limited external morphological differentiation. However, the male palps relative complexity aids the identification, although their size still provides a challenge. The study of male median apophyses or the stalk of female spermathecae often requires high-resolution microscopy. The genus *Cataleptoneta* Denis, 1955 was placed as a subgenus of *Barusia* by Kratochvíl (1978) and was so listed by Brignoli (1983), but if the taxa are congeneric, *Cataleptoneta* has priority as the generic name. These two genera are provisionally kept separate, treated as two distinct genera in the World Spider Catalog (WSC 2026), despite Ledford et al. (2021) recovering both as paraphyletic in their phylogenomic study.

The genus *Cataleptoneta* consists of seven European species: *Cataleptoneta aesculapii* (Brignoli, 1968), *C. aydintopcui* Demircan, 2020, and *C. sbordonii* (Brignoli, 1968) in Turkey; *C. detriticola* Deltshev & Li, 2013 in Bulgaria and Greece; *C. lingulata* Wang & Li, 2010 in Croatia; *C. semipinnata* Wang & Li, 2010 in Greece; *C. sengleti* (Brignoli, 1974) in Greece (Crete). In addition, the type species occurs outside Europe, in Lebanon and Israel: *C. edentula* Denis, 1955 (Nentwig et al. 2026; Gavish-Regev et al. 2016).

In this paper we describe a new species of *Cataleptoneta* from the Vromoner Canyon caves. *Cataleptoneta sulfur* sp. nov. can be distinguished from the other species by the unique setae with 3-petaled tulip-shaped bases. Detailed documentation of the diagnostic characters is provided via scanning electron micrographs, digital images, and drawings.

## Materials and methods

Specimens were collected by hand in Skordili’s Sulfur and in Turtle caves in Vromoner Canyon in the Sarandaporo River. These caves form an extensive hypogenic subterranean karst system along with Atmos Cave (Audy et al. 2022; Mareš et al. 2026). Later, the species was also found in caves in Pixaria Canyon (2.5 km upstream from Vromoner) and in Langarica Canyon (~35 km from Vromoner) (Fig. 10).

### Morphology

Specimens were preserved in 70% ethanol solution for morphological analysis and 96% ethanol solution for genetic analysis. External morphology was examined using a Nikon SMZ1270 stereomicroscope. Male and female specimens were photographed using a Kern ODC 825 microscope camera attached to a Kern OZL 464 trinocular stereomicroscope. Images of the male pedipalp and female vulva were taken using a Kern ODC 825 microscope camera mounted on a Kern OBN 132 trinocular optical microscope.

Scanning electron micrographs were obtained by a Phenom Pro scanning electron microscope (Thermo Fisher Scientific) at an acceleration voltage of 15 kV. Illustrated samples were rehydrated and then fixed in 3% glutaraldehyde in 0.1 M sodium cacodylate buffer (pH 7.4). They were dehydrated through a graded ethanol series (30%, 50%, 70%, 80%, 90%, and 100%), transferred to mixed ethanol–hexamethyldisilazane (HMDS) solutions (3:1, 1:1, and 1:3), and finally placed in 100% HMDS (Nation, 1983). To ensure complete drying the samples were left overnight to allow full chemical evaporation. Dried specimens were mounted on aluminum stubs using double-sided conductive carbon adhesive tabs and sputter-coated with gold for 60 s (SEM Coating Unit E5100). Multifocal images were compiled using Zerene Stacker software.

The length of the leg segments was measured on their dorsal side (Table 1). All measurements are in millimeters and were taken with a millimetric ocular lens. Palpal tarsus, tibia, and femur spination models, copulatory organ structures, and the terminology of copulatory organs mainly follow Denis (1955), Brignoli (1968, 1974), Wang and Li (2010), Deltshev and Li (2013), Li et al. (2024), and Liu et al. (2024). Abbreviations used in the text or figures are as follows: 3 pts = setae with 3-petaled tulip-shaped base (of the male palpal tarsus); abs = apical bent seta (of the male palpal tarsus); ALE = anterior lateral eye; bs = bent seta (of the male palpal tarsus); bss = basal straight seta (of the male palpal tarsus); CO = conductor; EM = embolus; Fe = femur; l = lateral; MA = medial apophysis; Mt = metatarsus; PLE = posterior lateral eye; PME = posterior median eye; Pt = patella; Ta = tarsus; Ti = tibia; to = tarsal organ; VS = ventral sclerite.

**Table 1.** Leg measurements of male and female *Cataleptoneta sulfur* sp. nov.

		**Fe**	**Pt**	**Ti**	**Mt**	**Ta**	**Total**
Holotype (♂)	I	1.43	0.23	1.58	1.34	0.82	5.4
	II	1.13	0.21	1.10	0.92	0.61	3.97
	III	0.96	0.19	0.86	0.83	0.48	3.32
	IV	1.29	0.18	1.25	1.10	0.61	4.43
Paratype (♀)	I	1.39	0.18	1.56	1.25	0.82	5.2
	II	1.09	0.17	1.06	0.85	0.57	3.74
	III	0.90	0.16	0.78	0.74	0.48	3.06
	IV	1.20	0.16	1.15	1.00	0.59	4.1

The specimens were deposited in the zoological collection of the Department of Life Sciences, Sapientia Hungarian University of Transylvania, Romania (**ZC-DLSSAP**; curator Dr István Urák).

### Molecular methods

The prosoma and legs of ethanol-preserved specimens were used for DNA isolation using the QIAmp DNA Mini Kit according to the manufacturer specifications. A fragment of the cytochrome c oxidase subunit I mitochondrial gene (COI) was amplified using the protocol and primers as previously described (Urák et al. 2025) and Sanger sequenced in one direction by Macrogen (Amsterdam, The Netherlands). The low-quality ends of the resulting chromatograms were trimmed using Chromas 2.6.6 (Technelysium Ltd, South Brisbane, Australia) and CodonCode Aligner 3.7.1 (CodonCode Corporation, MA, USA) and frameshift mutations or early stop codons were checked in MEGA 7 (Kumar et al. 2016).

Taxon selection was based on Ledford et al. (2021), to cover as many taxa of the *Paraleptoneta* group as possible. The original matrix contained 51 sequences: for outgroups we used four *Calileptoneta*, six *Leptonetela*, a *Protoleptoneta*, and five *Leptoneta* sequences. For the ingroup 22 *Sulcia*, six *Barusia*, and six *Cataleptoneta* sequences were used (for full list see Suppl. material 1). The multiple alignment was performed using MAFFT (Katoh et al. 2019) and trimmed in BGME (Criscuolo and Gribaldo 2010), with the final alignment being 655 nucleotides long. Model selection via SMS (Lefort et al. 2017) resulted the GTR G+I substitution model. Maximum-likelihood tree was inferred in PhyML (Guindon et al. 2010) on the NGPhylogeny server (Lemoine et al. 2019). Bootstrap values were calculated according to the method of Lemoine et al. (2018), and the final tree was edited in iTol (Letunic and Bork 2024). Due to the diversity of sequence lengths, we experienced suboptimal overlaps and low support values, so we had to delete taxa which did not sufficiently overlap. Also, the genus *Sulcia* was overrepresented; thus, we reduced the number of its species to three. The reduced dataset consists of 30 taxa and 417 bases. The phylogenetic analysis was performed the same way as described above.

Two species delimitation methods were used: ASAP (Assemble Species by Automatic Partitioning; Puillandre et al. 2021) and the tree-based method bPTP (Bayesian Poisson Tree Processes; Zhang et al. 2013), as implemented in the iTaxoTools 0.1 toolkit (Vences et al. 2021).

The map was created in QGIS 3.28.4 (QGIS.org 2026) using datasets downloaded from OpenTopography (Shuttle Radar Topography Mission-derived 30 m digital elevation model), OpenStreetMap (rivers and lakes shapefiles), and NaturalEarth (country borders shapefiles). Graphical edits to some of the figures were done in Inkscape 1.4 (Inkscape.org 2026).

## Results

### Cataleptoneta sulfur

Taxon classification

Animalia

AraneaeLeptonetidae

Urák, Sarbu & Szűts sp. nov.

FB7BD3BB-7CEE-5710-A2DF-3EF6A7C641BC

https://zoobank.org/A96B0037-C093-4ECA-839A-ACE47C2C46AD

#### Type material.

***Holotype* male** from Skordili’s Sulfur Cave. Sarandaporo River Valley, Vromoner Canyon on the Greek–Albanian border; (40.0961°N, 20.6789°E); 400 m; 11 November 2023; leg. S.M. Sarbu; (ZC-DLSSAP).

***Paratypes***: • 3 males and 5 females; same data as for holotype; • 2 females; same locality; June 2023; • 12 males and 15 females; same locality; 03 April 2023; leg. S.M. SArbu & I. Urák; • 6 males and 3 females; samle locality, Novermber 2024; leg. S.M. Sarbu. **Albania: Turtle Cave** • 2 males and 1 female; 04 May 2023; leg. S.M. Sarbu; • 4 males and 8 females; 03 April 2023; • 4 males and 7 females; 08 November 2023; • 3 males and 1 females; 19 July 2025; **Pixaria Cave** • 2 females; 23 June 2023; leg. S.M. Sarbu; **Langarica Cave**, • 2 males and 1 female; June 2024; leg. S.M. Sarbu; • 2 males and 21 ; November 2024; leg. S.M. Sarbu.

#### Diagnosis.

*Cataleptoneta sulfur* Urák, Sarbu & Szűts, sp. nov. differs from all congeners by the presence of the three setae with modified bases in the tarsal depression in retrolateral view. The setal bases resemble a tulip with three petals (Fig. 7D) in dorsal view. Apical to these setae directly a thin bent seta (bs – Fig. 7C), further to the tip a thick seta (abs – Fig. 7C) is located. Proximal to these modified based setae, is a thin straight seta (bss – Fig. 7C).

#### Etymology.

The species name is a noun in apposition, and it refers to Sulfur Cave, from where the first specimens were collected. Sulfur Cave has its entrance in Greece, but its deeper sections extend into Albania. The cave is renowned for its exceptionally rich chemosynthesis-based subterranean community (Sarbu et al. 2024), as well as for the extraordinarily large spider web and dense spider colonies it harbors (Urák et al. 2025).

#### Description.

**Figure 1.**
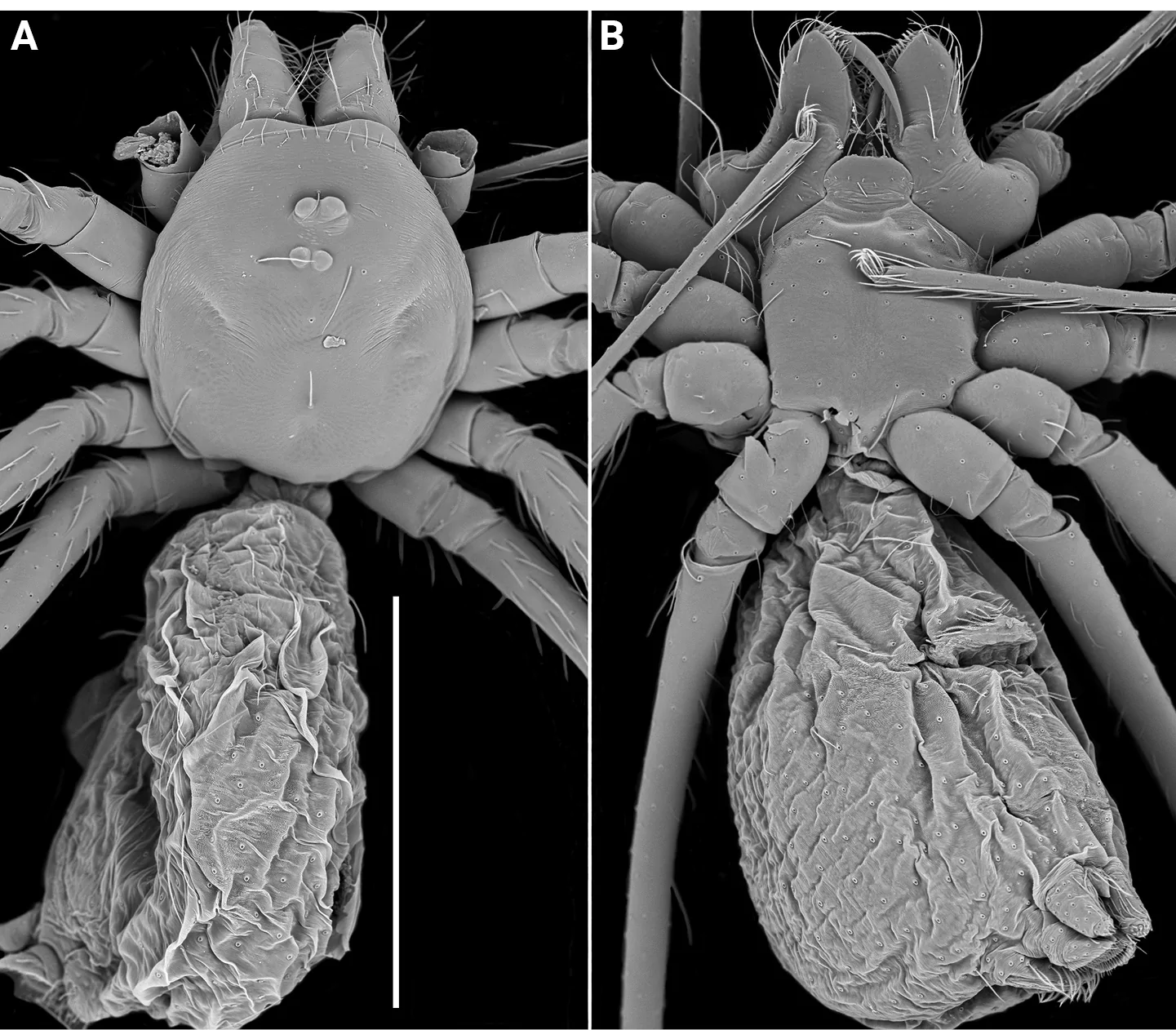
Male habitus of *Cataleptoneta sulfur* sp. nov. Scanning electron micrographs. **A**. Dorsal view; **B**. Ventral view. Scale bar: 0.5 mm.

**Male (holotype)**: total length 1.26. Carapace 0.45 long, 0.41 wide. Opisthosoma 0.81 long, 0.47 wide. Clypeus 0.12 heigh. Sternum 0.36 long, 0.33 wide. The entire body pale and highly depigmented. Carapace pale yellow, clypeus slightly dark orange-yellow. Opisthosoma pale yellow, but in some specimens faintly patterned with long, cylindrical spots. With six eyes, 4 contiguous eyes on anterior recurved row and 2 subcontiguous eyes on posterior row. Ocular area 0.10 long, 0.09 wide anteriorly. Eye measurements: diameter of ALE 0.02, PLE 0.02 (absent in some specimens), PME 0.01, distance of ALE-PME 0.06, PLE-PME 0.04. Chelicerae and endites slightly darker orange-yellow or yellow-brown, with 8 teeth on promargin and 4 small teeth on retromargin. Gnathocoxa with 20 denticles. Endites, labium and sternum yellowish. Legs long, slender, and yellow, tarsus with 2 or 3 spines; leg formula I, VI, II, III (Table 1). Palpal femur: 0.70, patella: 0.30, tibia: 0.32, tarsus: 0.28, total: 1.6. Two lyriform organs retrolaterally present on the palp: one at the apical portion of the femur, immediately proximal to the femur-patella articulation, and the other located anterior to the midpoint of the patella. Palpal femur with three strong prolateral spines and 9 or 10 spines retrolaterally, originating from small tubercles, with two strong macrosetae apically on bifid apophysis. Patella with one long dorsoapical macroseta. Tibia prolateral side with 3 strong macrosetae, evenly distributed (Figs 3A, 4A); ventral side with 6 macrosetae (Figs 3B, 4B), 1 proximal (broken off but base visible – Fig. 6E), 3 clustered in the middle of the segment (Fig. 6E), 2 on the apical end (Fig. 6E): retrolateral side with a thin basal macroseta and 4 or 5 apical macrosetae. Palpal tarsus with a dorsal transverse depression with a deep division (Fig. 7C), basal part longer (~2/3 of the total length) and with a retrolateral outgrowth (Fig. 7C), retrolateral outgrowth with 3 delicate setae with modified bases (marked red in Fig. 5B, with 3 pts – Figs 6A, 6B, 7C), shaped like a 3-petaled tulip (Fig. 7D) and with a bent seta, with straight seta (bss – Fig. 7C) basal to the outgrowth, with retrolateral bent seta (abs – Figs 5B, 6B, 6C, 7E) distal to palpal tarsus. Bulb with spoon-shaped embolus originating from prolateral side (Figs 3A, 4A, 5A, 8), conductor elongated (Figs 3A, 4A, 5A, 8), with a filamentous end (Fig. 7A), median apophysis originating on dorsal side of bulb (Figs 5A, 8).

**Figure 2.**
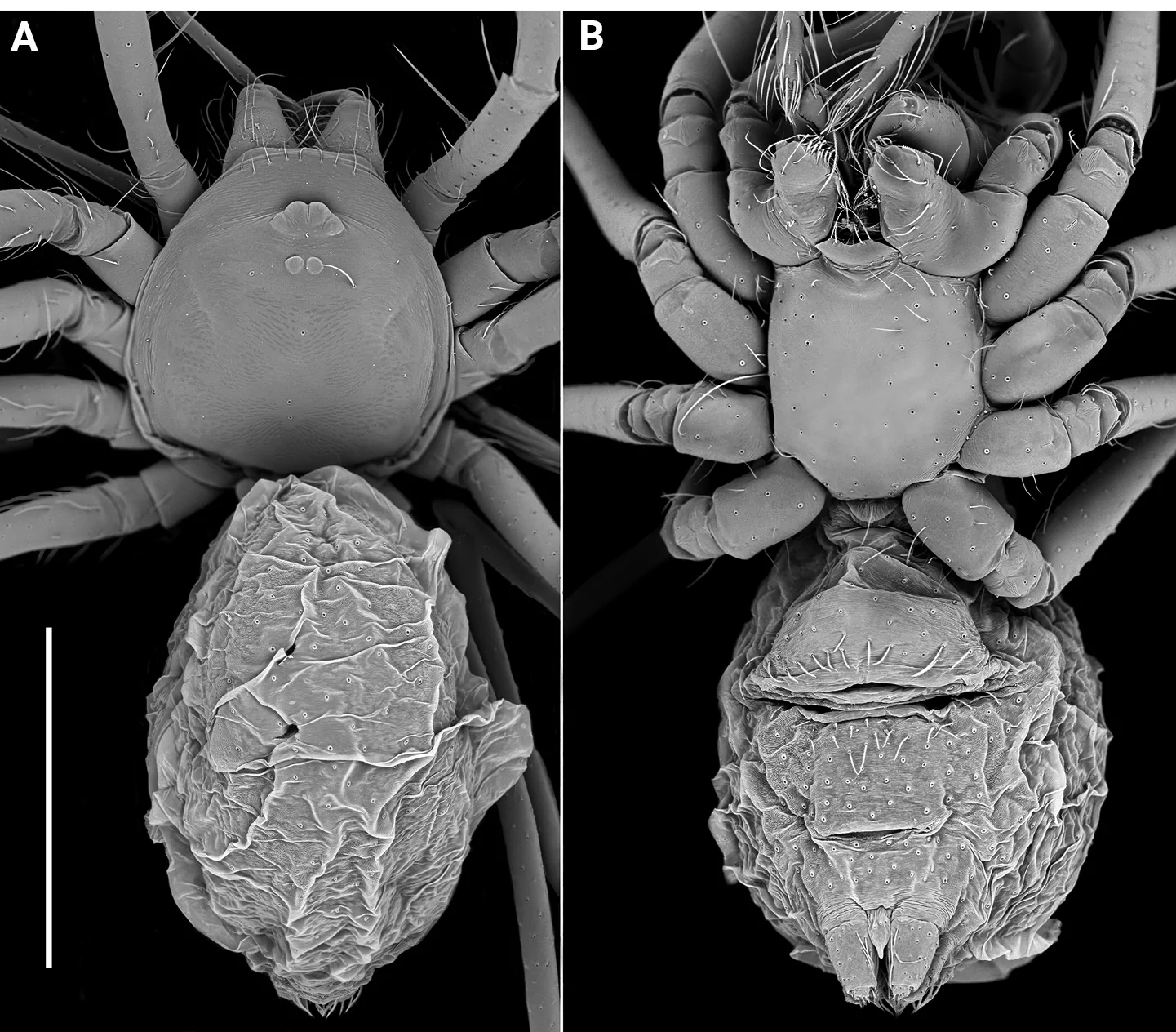
Female habitus of *Cataleptoneta sulfur* sp. nov. Scanning electron micrographs. **A**. Dorsal view; **B**. Ventral view. Scale bar: 0.5 mm.

**Figure 3.**
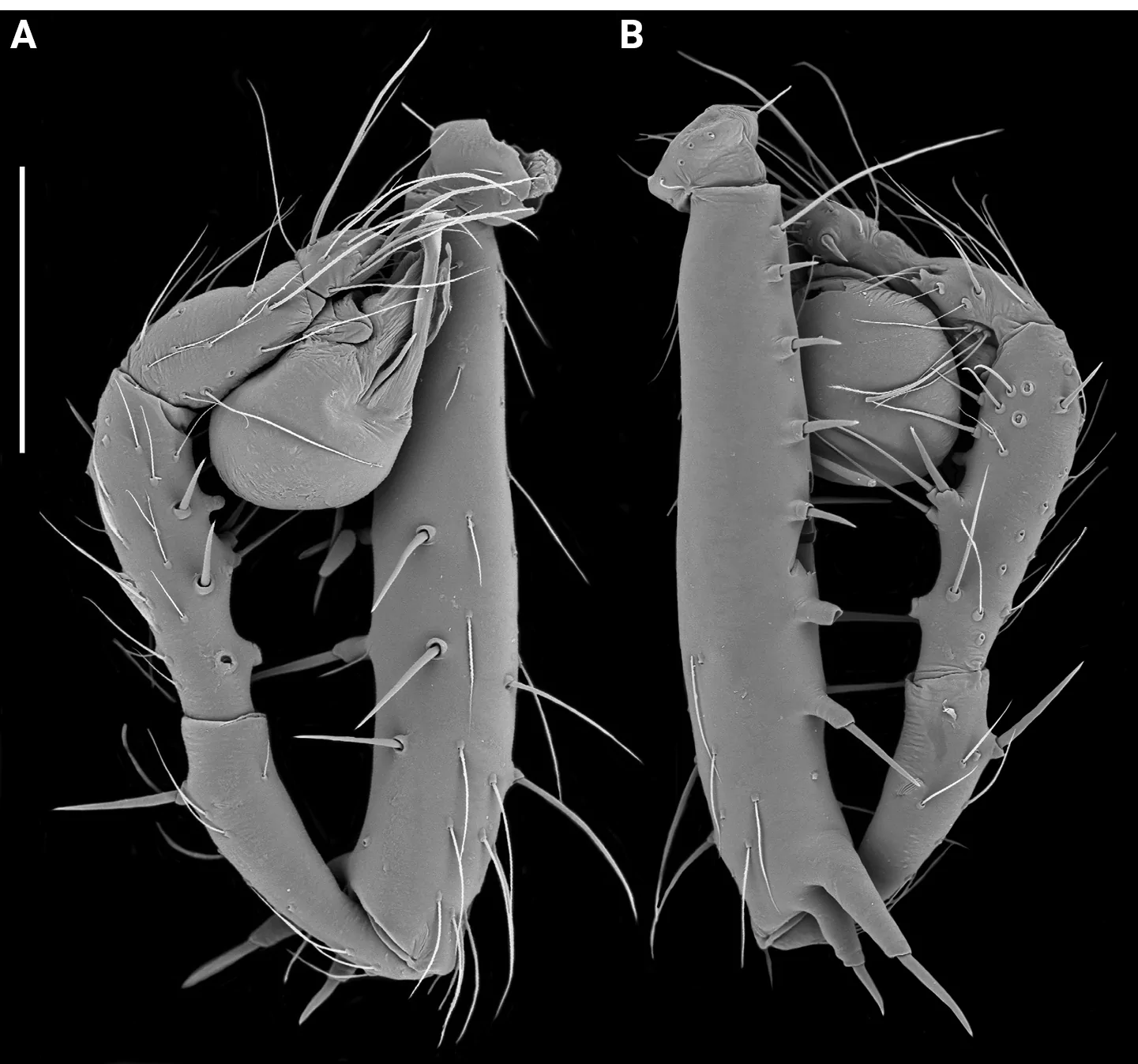
Male palp of *Cataleptoneta sulfur* sp. nov. Scanning electron micrographs. **A**. Prolateral view; **B**. Retrolateral view. Scale bar: 0.25 mm.

**Figure 4.**
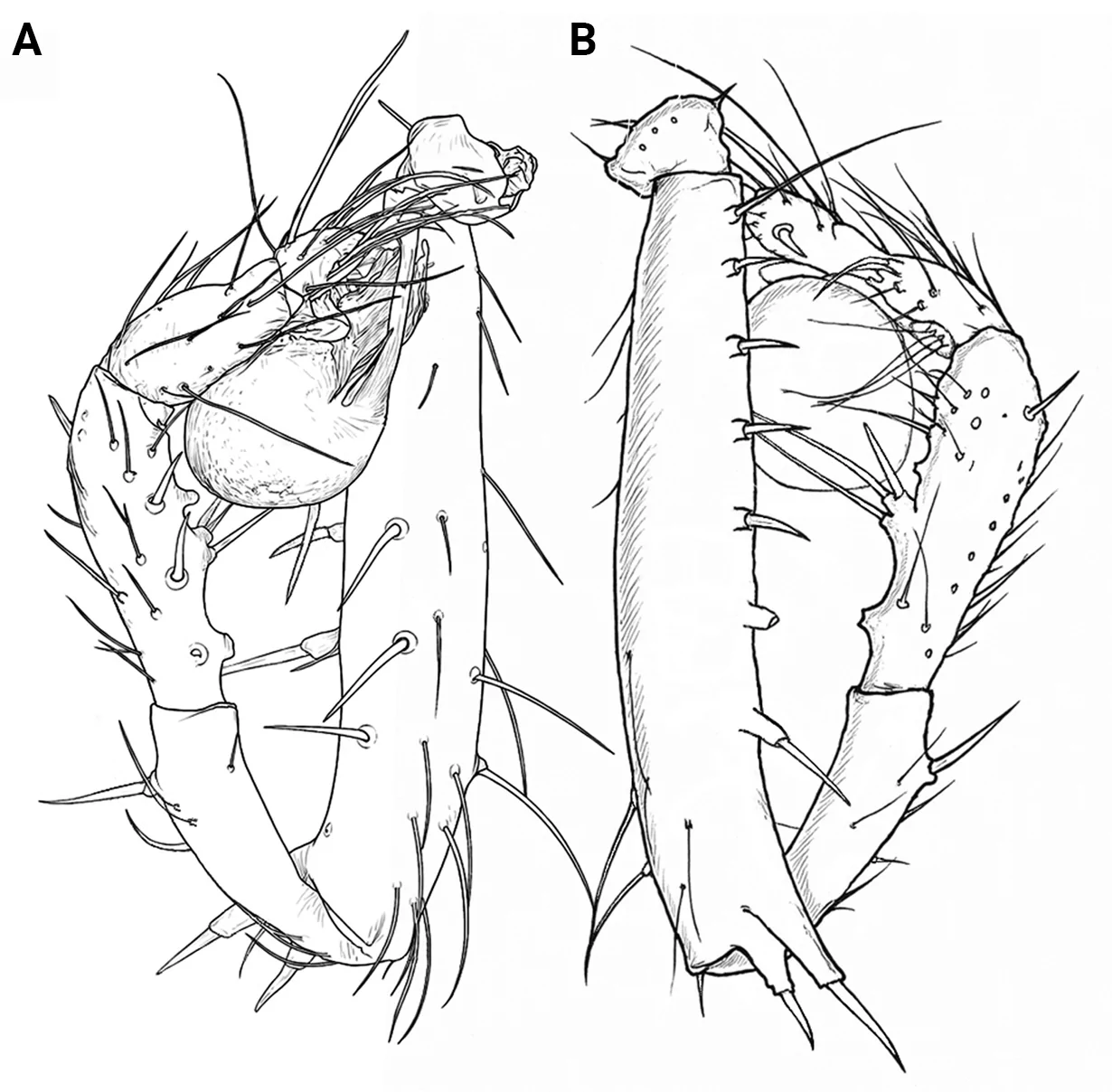
Male palp of *Cataleptoneta sulfur* sp. nov. **A**. Prolateral view; **B**. Retrolateral view. Scale bar: 0.25 mm.

**Figure 5.**
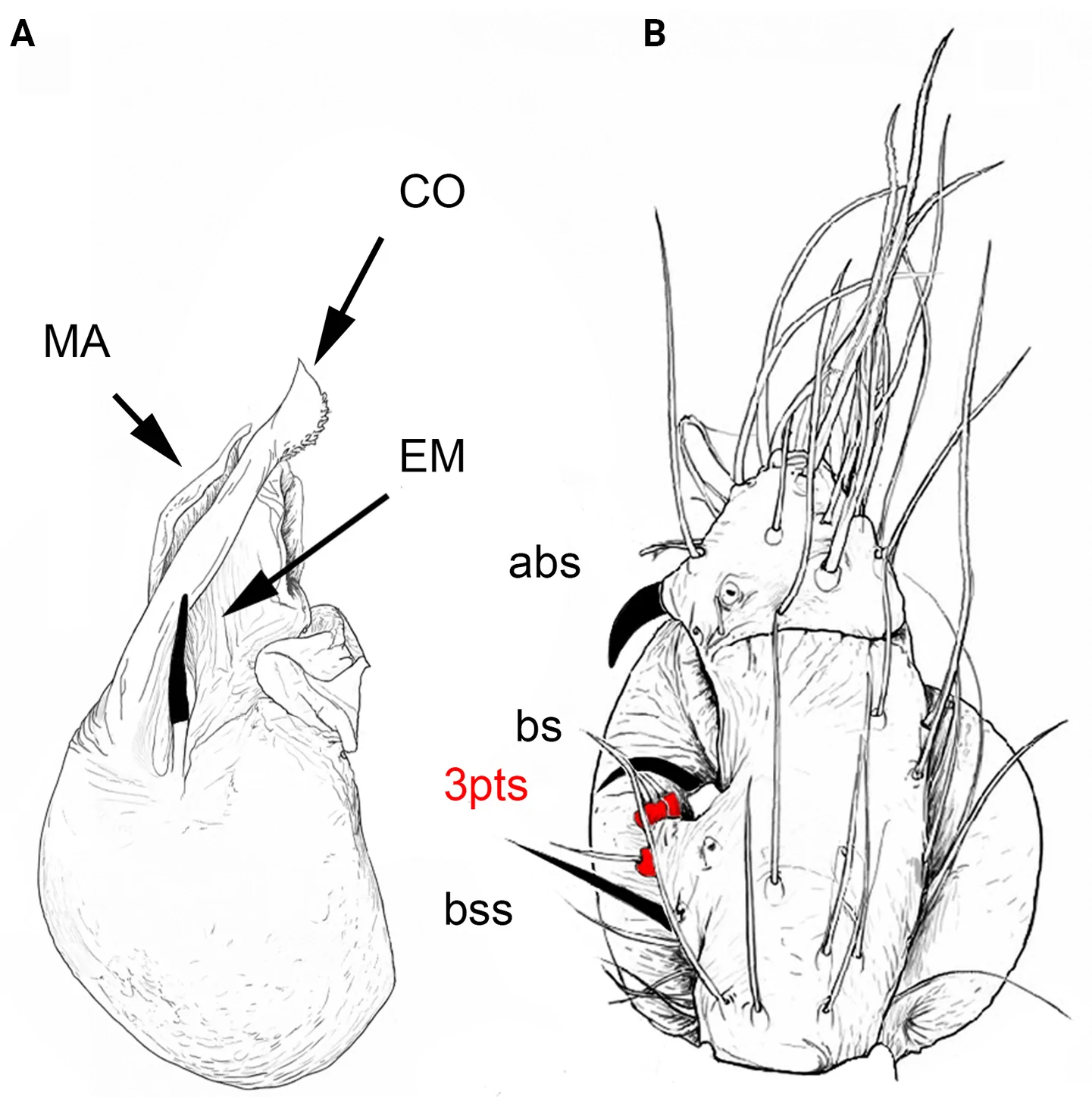
Male palp of *Cataleptoneta sulfur* sp. nov. **A**. Bulbus, prolateral view; **B**. Palpal tarsus dorsal view. Abbreviations: CO – conductor, EM – embolus, MA – median apohysis, abs – apical bent seta, bs – bent seta, 3 pts – seta with 3-petaled tulip-shaped base.

**Figure 6.**
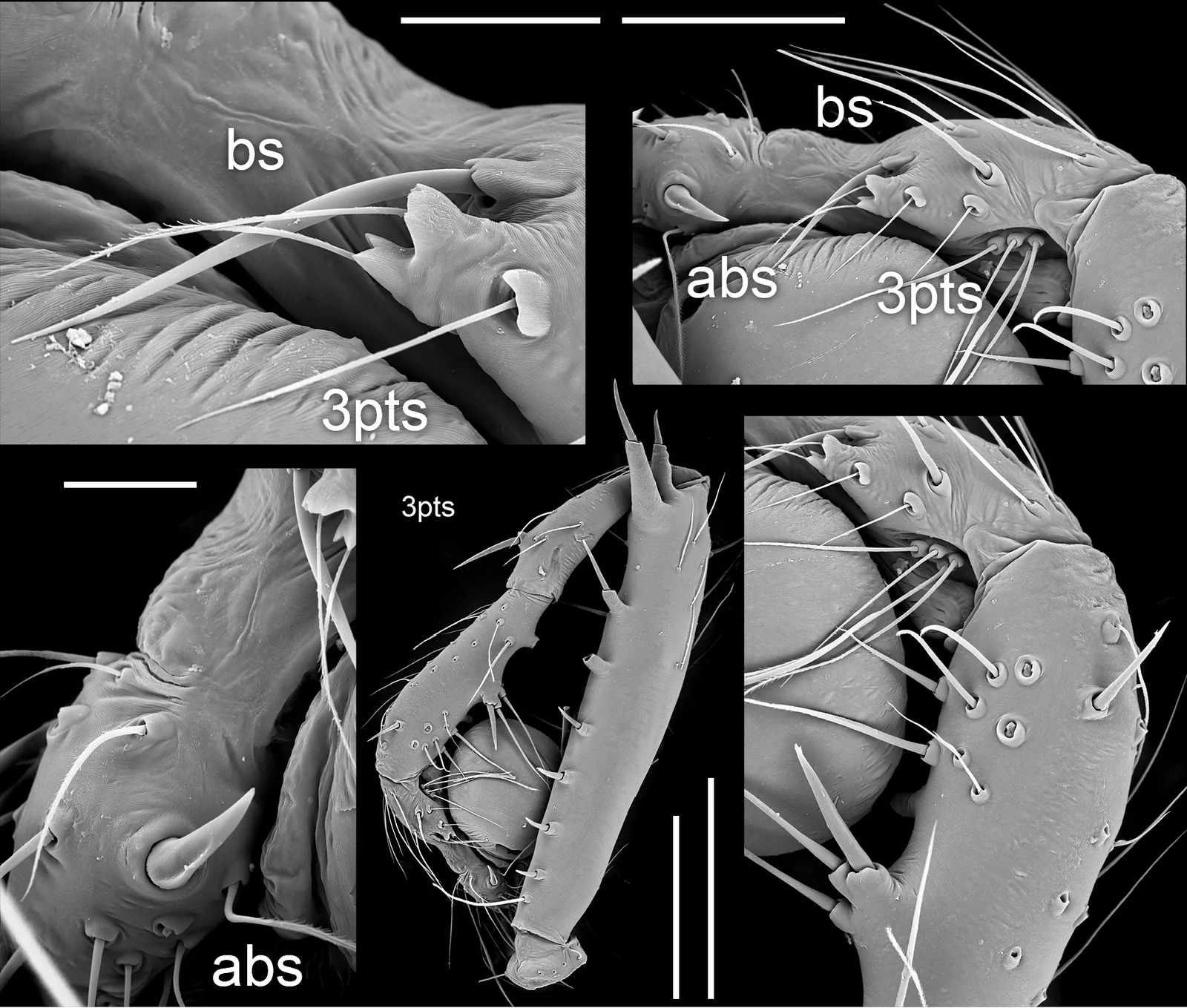
Male palp of *Cataleptoneta sulfur* sp. nov., closeups of the diagnostic characters, scanning electronmicrographs, retrolateral views. **A**. Palpal tarsal transverse depression, bent seta and three medial setae with 3-petal tulip-shaped bases; **B**. Palpal tarsus, proximal part; **C**. Palpal tarsal apical dorsal bent seta; **D**. Male full palp, **E**. Palpal tibia. Abbreviations: abs – apical bent seta, bs – bent seta, 3 pts – seta with 3-petaled tulip-shaped base. Scale bars: 0.25 mm (**D**); 0.1 mm (**B, E**); 0.03 mm (**A, C**).

**Figure 7.**
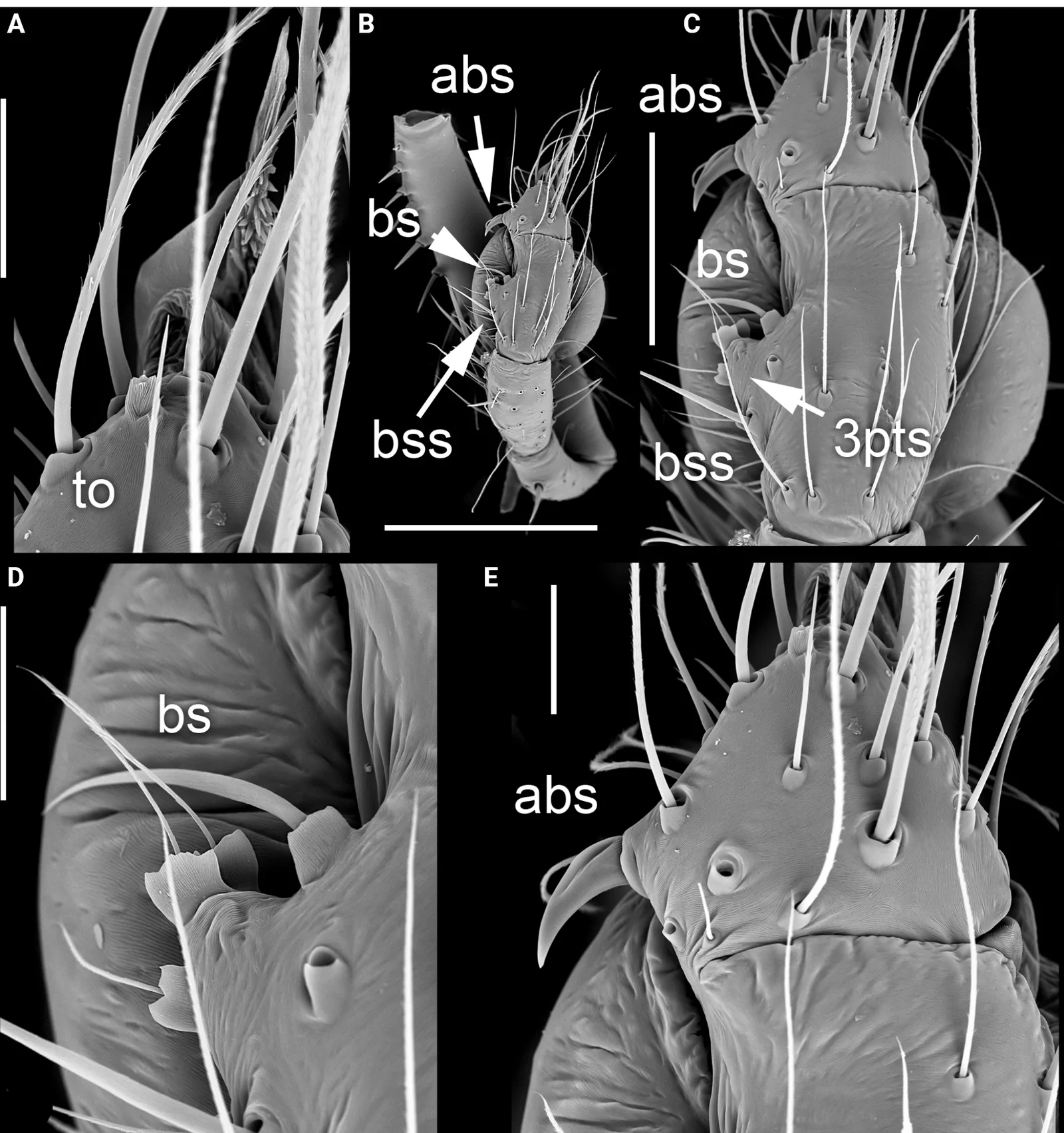
Male palp of *Cataleptoneta sulfur* sp. nov., cymbial diagnostic characters, scanning electronmicrographs, dorsal views. **A**. Tip of the palpal tarsus, showing the conductor’s dorsal side; **B**. Full palp, dorsal view; **C**. Palpal tarsus, dorsal view; **D**. Bent seta and three medial setae with 3-petaled tulip-shaped bases; **E**. Palpal tarsus, apical bent seta. Abbreviations: abs – apical bent seta, bs – bent seta, bss – basal straight seta, 3 pts – seta with 3-petaled tulip-shaped base, to – tarsal organ. Scale bars: 0.25 mm (**B**); 0.1 mm (**C**); 0.03 mm (**A, D, E**).

**Figure 8.**
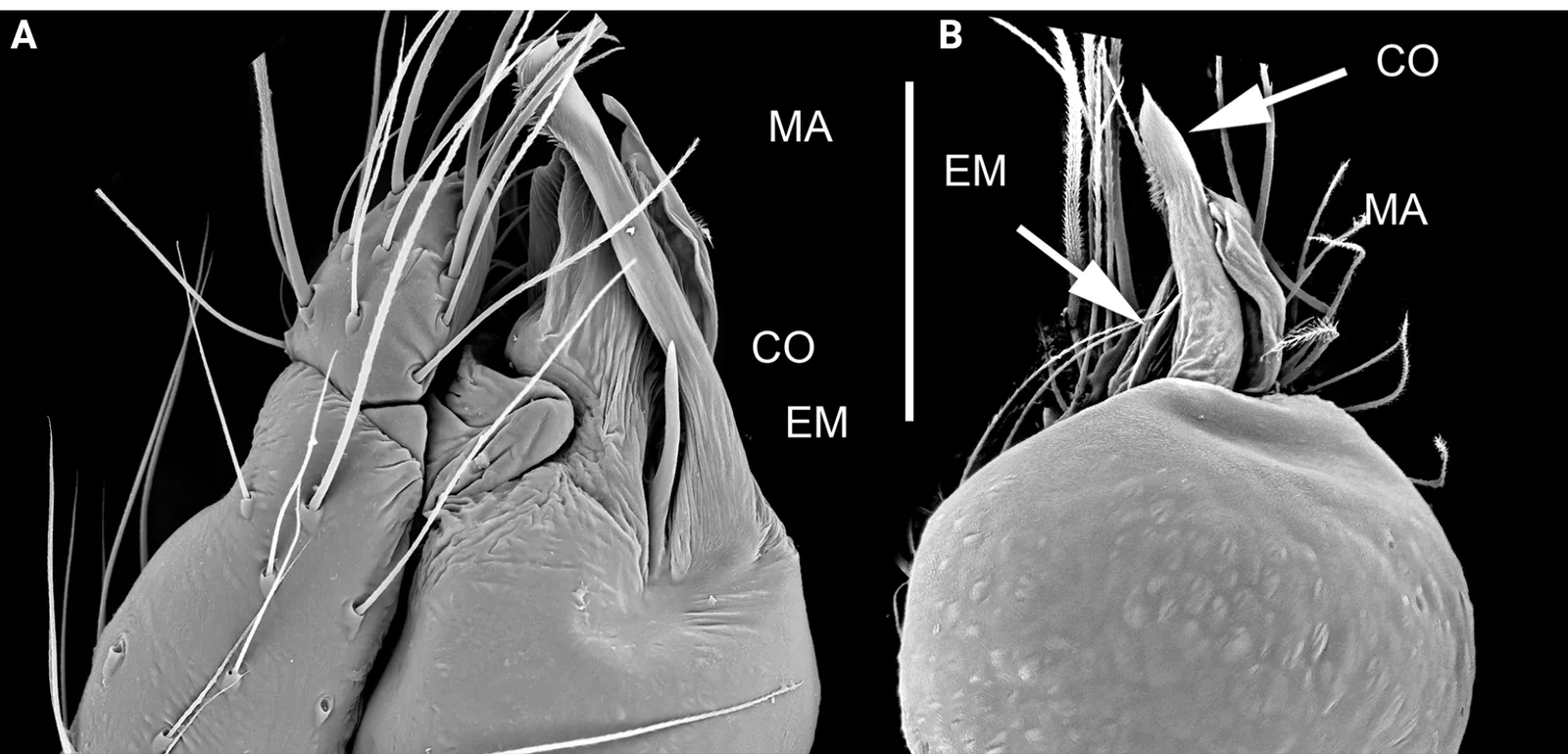
Male palpal bulb of *Cataleptoneta sulfur* sp. nov. Scanning electronmicrographs. **A**. Tip of the palpal tarsus and bulb, prolateral view; **B**. Bulb, ventral view. Abbreviations: CO – conductor, EM – embolus, MA – median apohysis. Scale bar: 0.1 mm.

**Figure 9.**
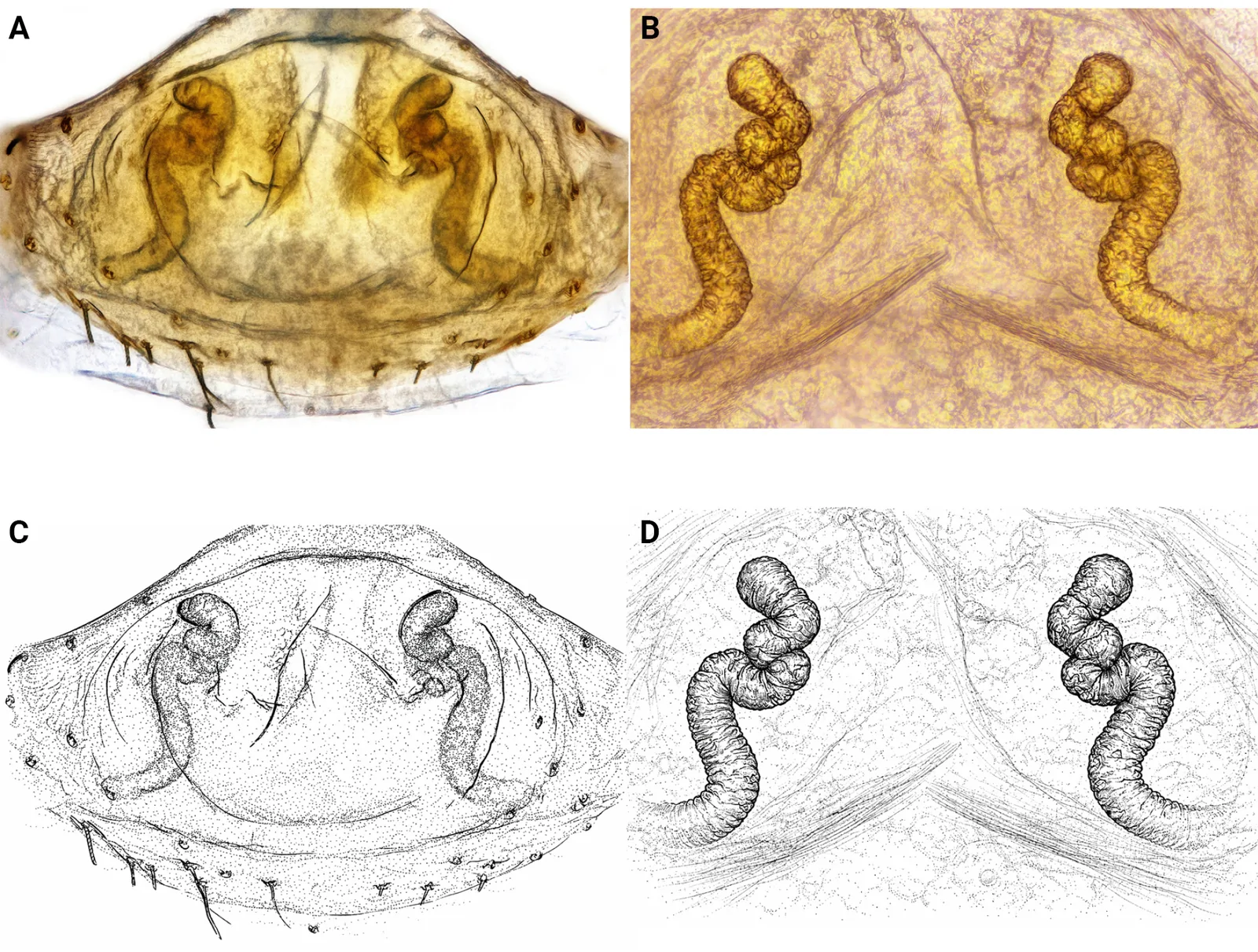
Female genitalia of *Cataleptoneta sulfur* sp. nov. Micrographs and drawings. **A, C**. Ventral view; **B, D**. Vulva, dorsal view. Scale bar: 0.1 mm.

**Female (paratype)**: similar to male in coloration and general features, but with a larger body size and shorter legs (Figs 1, 2). Total length 1.26. Carapace 0.5 long, 0.42 wide. Opisthosoma 0.76 long, 0.53 wide. Clypeus 0.11 heigh. Sternum 0.39 long, 0.32 wide. Carapace pale yellow, opisthosoma whitish with pale gray pattern, visible only in some specimens, suboval. Six eyes with 4 contiguous eyes on anterior recurved row and 2 subcontiguous eyes in posterior row. Ocular area 0.10 long, 0.09 wide anteriorly. Eye measurements: ALE 0.03, PLE 0.02, PME 0.01, ALE-PME 0.06, PLE-PME 0.04, posterior median eyes smaller than anterior eyes. Chelicerae yellowish with 7 teeth on promargin and 3 small teeth on retromargin. Gnathocoxa with 20 denticles. Endites, labium, and sternum yellowish. Legs long, slender, and yellow, tarsus with 2 or 3 spines; leg formula I, VI, II, III (Table 1). Female palp with a long claw, femur: 0.64, patella: 0.20, tibia: 0.41, tarsus: 0.47, total: 1.72. Genital area (Fig. 2B) covered with hairs. Internal genitalia (Fig. 9) simple, with a pair of spermathecae and weakly sclerotized, very elongate, S-shaped sperm ducts, with a characteristic inwardly directed medial twist (Fig. 9B, D).

#### Variation.

The number of retrolateral macrosetae on the male palpal femur, with tubercles, may vary either nine or ten: one long and slender spine basally, followed by four or five shorter and thicker spines (four on the left and five on the right pedipalp in the holotype), two slightly longer spines medially arising from somewhat larger tubercles, and two strong spines apically on a bifid apophysis.

#### Biology.

*Cataleptoneta sulfur* sp. nov. thrives in the sulfidic caves in southeastern Albania and northwestern Greece. Numerous specimens have been observed on the cave walls and on speleothemes in moist areas in these caves, often close to the sulfidic streams and lakes where they build simple, flat webs. Males, females, and juveniles have been observed throughout the year as conditions in the caves are constant and they are not affected by surface daily or seasonal changes. Their food consists of collembola which are unusually abundant in these caves, where they graze on the rich chemoautotrophic sulfur-oxidizing biofilms that cover the moist cave walls (Sarbu et al. 2024; Urák et al. 2025). Several other small predators such as mites, pseudoscorpions, carabids, and centipedes rely on the same autochthonous food source.

#### Distribution.

**Figure 10.**
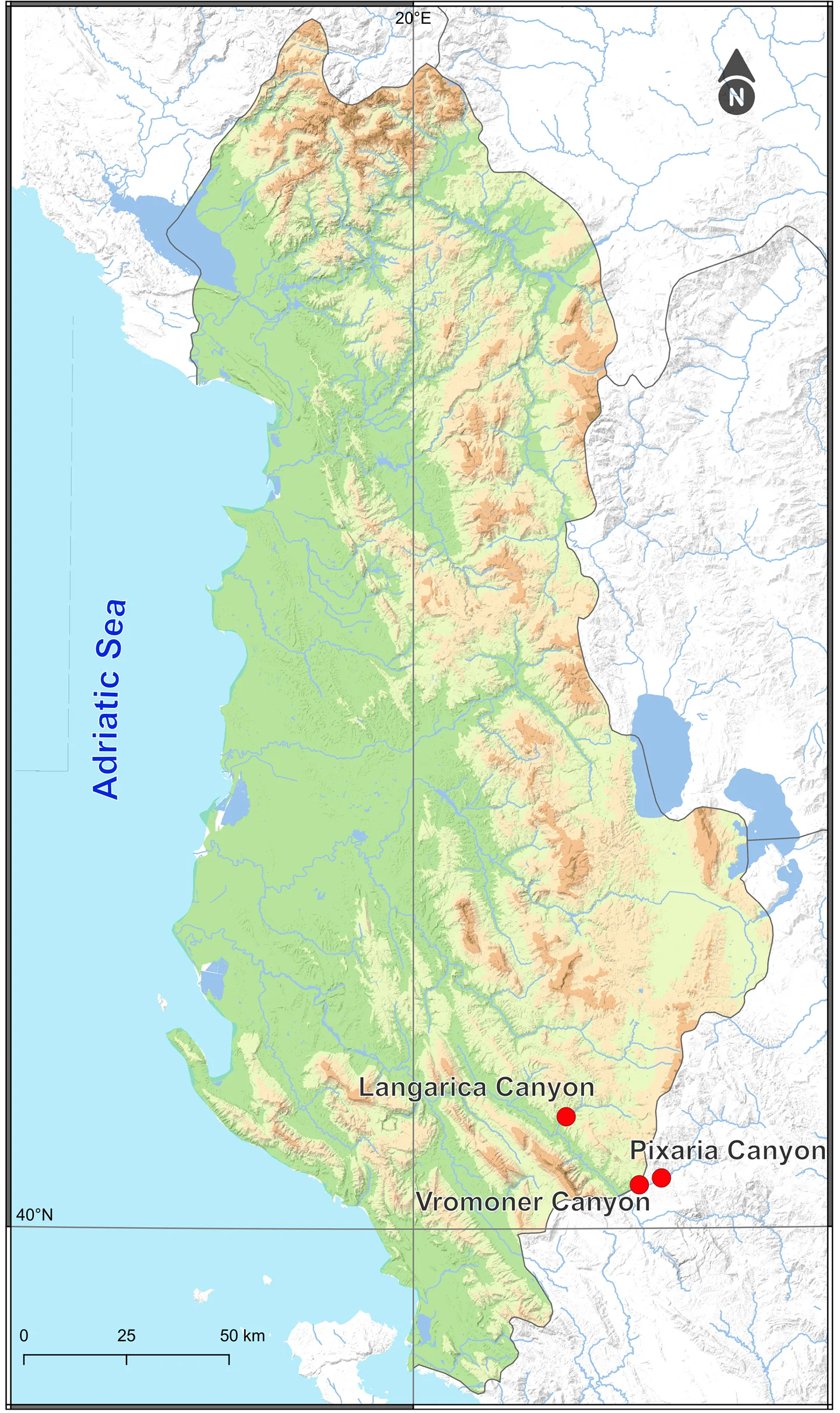
Map of Albania and Greece showing the locations of *Cataleptoneta sulfur* sp. nov.

Greece and Albania, in the deep aphotic sections of Skordili’s Sulfur, Turtle, Pixaria, and Langarica caves (Fig. 10).

#### Molecular results.

**Figure 11.**
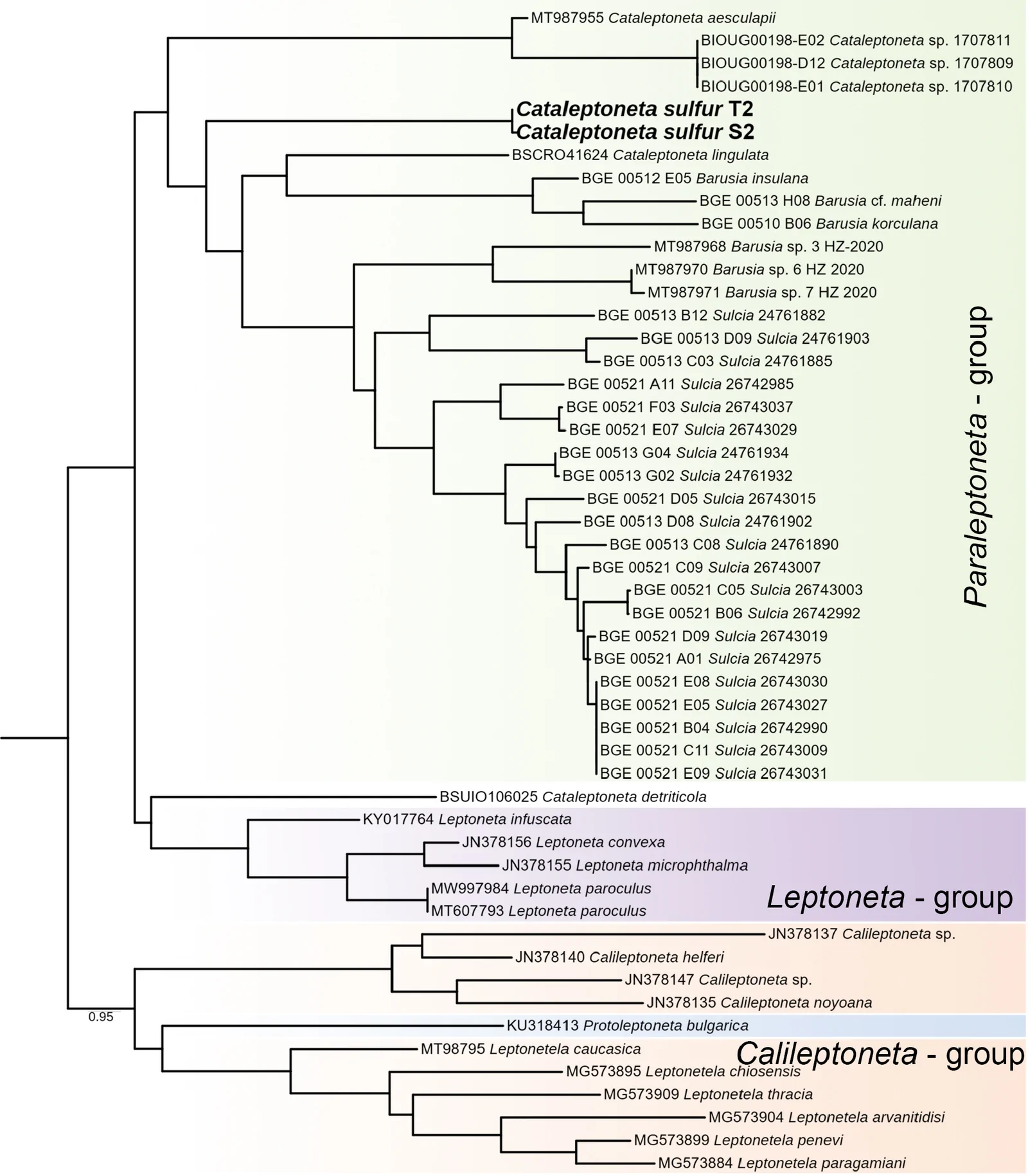
Maximum-likelihood tree of all the available *Paraleptoneta*-group COI sequences. Color schema adapted from Ledford et al. (2021).

**Figure 12.**
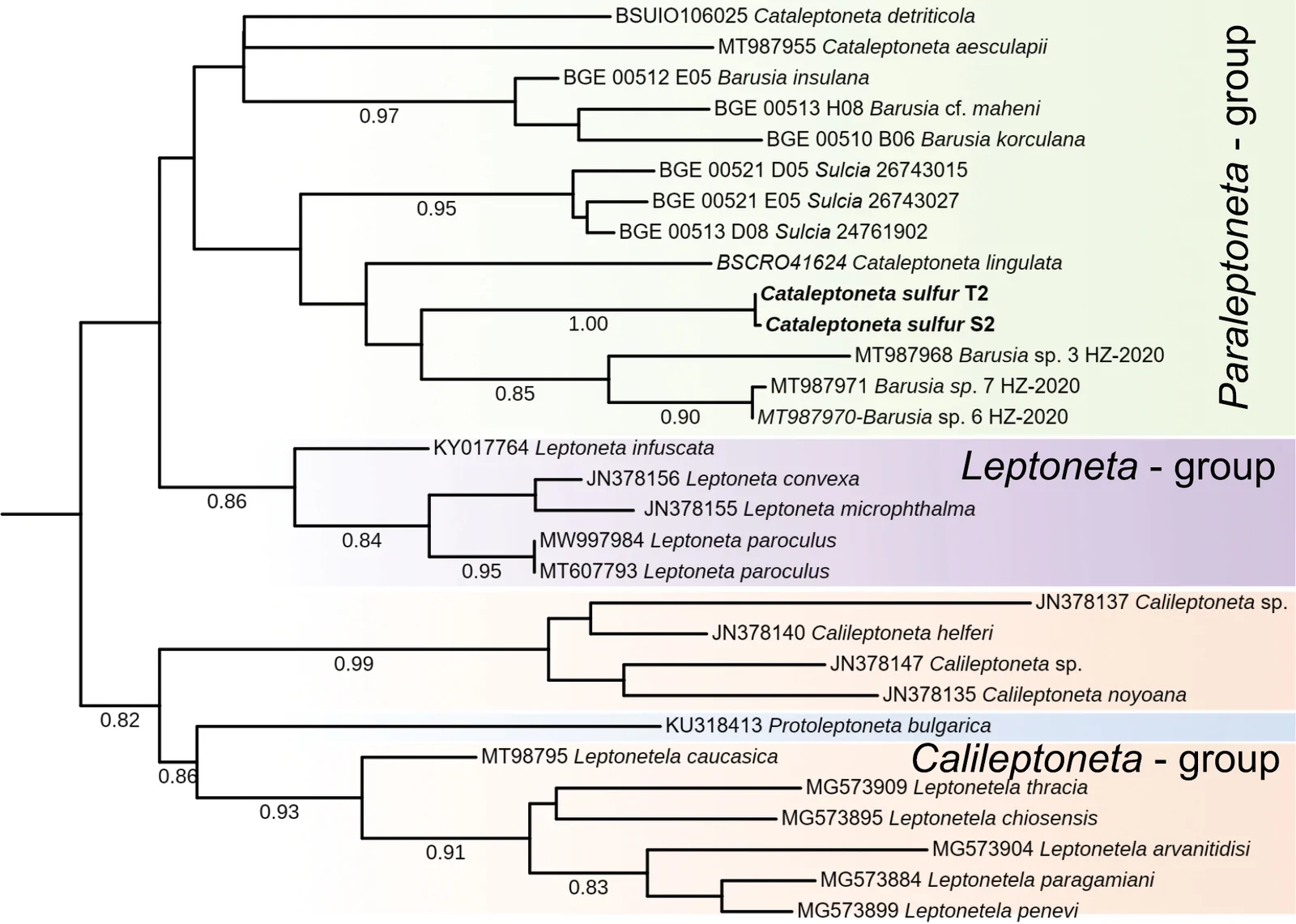
Maximum-likelihood tree of the reduced dataset. Color schema adapted from Ledford et al. (2021).

Two COI sequences were obtained; one from a Skordili’s Sulfur Cave and one from a Turtle Cave specimen, each with a length of 642 bp. The sequences belong to two distinct haplotypes. Hap1 is present in Sulfur Cave, and Hap2 is present in Turtle Cave (GenBank accession numbers PZ139234 and PZ139235, respectively). The *p*-distance value of 0.008 between the two haplotypes indicates a low genetic differentiation. Both phylogenetic analyses (Figs 11, 12) placed *Cataleptoneta sulfur* sp. nov. in the *Paraleptoneta* group. In both analyses, only *Sulcia* was monophyletic, and *Barusia* and *Cataleptoneta* were rendered paraphyletic. The larger matrix’s analysis placed *Cataleptoneta sulfur* sp. nov. in a basal position within the group (Fig. 11). The pruned dataset’s analysis placed *C. sulfur* sp. nov. in a clade with *C. lingulata* with three samples of *Barusia* (not identified to species). Sister to this clade was the monophyletic *Sulcia*. Sister to this clade we have the group including the other two *Cataleptoneta* species, *C. aesculapii* and *C. detriticola*, which cluster on a different clade with *Barusia insulana* (Kratochvíl & Miller, 1939), *B. korculana* (Kratochvíl & Miller, 1939) and *B. cf. maheni* (Kratochvíl & Miller, 1939). Both the ASAP and bPTP species delimitation methods clearly distinguish *Cataleptoneta sulfur* sp. nov. as a distinct species.

**Table 2.** Additional row of *Cataleptoneta sulfur* sp. nov. to compare to Demircan’s (2020) table.

**Species**	**Spine/s of palpal tarsus**	**Spine of palpal patella**	**Spine/s of palpal tibia**	**Spines and tubercles of palpal femur**	**Teeth of chelicerae Promargin / Retromargin**	
					♀	♂
*Cataleptoneta sulfur* sp. nov.	1 bent macroseta (ra)	1 short spine (da)	(1-1-1) straight macroseta (p)	2 digitiform apophyses (ra)	7 / 3	8 / 4
	1 bent seta (r)		(1-3-2) macroseta (v)	3 small tubercles (p)		
	1 straight seta (b)		2 macroseta (r)	~7-8 short spines (r)		

## Discussion

This study describes a new species of *Cataleptoneta*, thus adding to the known diversity of the genus and contributes to the spider fauna of Greece and Albania, as well as to their cave fauna (Mammola et al. 2022). The number of known species of genus *Cataleptoneta* has increased to nine worldwide and to eight in Europe, and this is the first species of this genus in Albania. They all occur in the eastern Mediterranean (Denis 1955; Brignoli 1968, 1974, 1978; Wang and Li 2010; Deltshev and Li 2013; Gavish-Regev et al. 2016; Bayram et al. 2017; Demir and Seyyar 2017; Demircan 2020). The distribution pattern of the genus indicates that further species are likely to occur in caves in the Mediterranean region and their discovery is a matter of further studies.

The COI marker was previously used in leptonetid taxonomy. Oh et al. (2022) described the cryptic diversity of Korean leptonetid spiders, while Wang et al. (2017) used an integrative taxonomy approach to describe 46 new leptonetid species. The genetic data generated in this study provides information into the taxonomic affiliation of the new species described herein and might be helpful for future research. Based on our analyses *Cataleptoneta sulfur* sp. nov. belongs to a group sister to *Sulcia*. It contains *C. lingulata* and three species identified as *Barusia*. Using ultraconserved elements (UCE) data in a phylogenomics study, Ledford et al. (2021) highlighted the paraphyly of *Barusia* and *Cataleptoneta*. Without the examination of those *Barusia* vouchers, it is impossible to further evaluate our findings. A well-sampled integrative study is required to solve these paraphyletic groups, which may be a result of using plesiomorphies as diagnostic characters.

## Supplementary Material

XML Treatment for Cataleptoneta sulfur

## Additional information

### Artificial Intelligence (AI) use

The authors accept full responsibility for the content of the manuscript, including the disclosure of any use of AI.

No AI tools were used in the preparation of this manuscript.

### Author ORCIDs

I. Urák https://orcid.org/0000-0003-2130-5068

A. Ştefan https://orcid.org/0000-0003-2339-210X

R.M. Motoc https://orcid.org/0000-0002-2724-8458

T. Brad https://orcid.org/0000-0002-6749-4338

A.-R. Zsigmond https://orcid.org/0000-0003-3999-2548

S.M. Sarbu https://orcid.org/0000-0001-8126-7193

T. Szűts https://orcid.org/0000-0001-8954-0641

### Data availability

All of the data that support the findings of this study are available in the main text or Supplementary Information.

## Supplementary materials

Supplementary material 1 Contains the phylogenetic analyses' data, alignment, model selection and trees in parenthetical format Data type docx https://binary.pensoft.net/file/1802616This dataset is made available under the Open Database License (http://opendatacommons.org/licenses/odbl/1.0/). The Open Database License (ODbL) is a license agreement intended to allow users to freely share, modify, and use this Dataset while maintaining this same freedom for others, provided that the original source and author(s) are credited. István Urák, Andrei Ştefan, Rozalia Magda Motoc, Traian Brad, Andreea-Rebeka Zsigmond, Serban M. Sarbu, Tamás Szűts
