# Supplementary material for: A new Cataleptoneta species (Araneae, Leptonetidae) from sulfidic caves in Greece and Albania
Source: Zookeys. 2026 Oct 6;1294:89–107. doi: 10.3897/zookeys.1294.202010 (PMC13662615; doi:10.3897/zookeys.1294.202010)
Supplement: Supplementary file 1 [file zookeys-1294-089_article-202010__-s001.docx]

Contents

[Large Matrix	1](#_Toc231138707)

[Final alignment	15](#_Toc231138708)

[Best Model	21](#_Toc231138709)

[ML Tree	22](#_Toc231138710)

[Small Matrix, final alignment	24](#_Toc231138711)

[Best Model	30](#_Toc231138712)

[ML Tree	31](#_Toc231138713)

[Species Delimitations ASAP	32](#_Toc231138714)

# Large Matrix

>BGE_00510_B06 Barusia korculana

GAGTTTATACTTTATATTTGGGTTGTGATCAGCTATGTTAGGTACTGCTATGTCTTTATTGATTCGACTTGAATTAAGACAGAGAGGTAGTTTTTTAGGAAATGATCATCTGTATAACGTTATTGTAACTGCTCATGCTTTTGTAATGATTTTTTTTATGGTTATGCCAATCATGATTGGGGGGTTTGGGAATTGGTTGATTCCTTTAATAATTGGGGCTCCTGATATAGCTTTTCCTCGAATAAATAATTTAAGTTTTTGATTACTACCTCCTTCTTTAGTTTTATTAATTGTTTCTAGAATAGTGGAGATTGGTGTAGGCACTGGATGGACCGTGTACCCGCCTTTGGCTTCGTATGTTGGACATGCTTACATTTCTGTGGATTTTGCTATTTTTTCTTTACATTTGGCTGGTGCTTCATCCATTATGGGGGCTATTAATTTTATTACTACAGTTATTAATATACGTAGAGGGGGGATAACTATGGAGAAAGTCCCATTGTTTGTTTGAAGAGTTTTGATTACAGCGGTTTTGCTATTATTATCTTTGCCTGTATTGGCGGGGGCTATCACCATATTACTTACTGATCGTAATTTTAATACATCATTTTTTGATCCAGCAGGAGGAGGGGATCCAGTCTTGTTTCAACATTTA

>BGE_00512_E05 Barusia insulana

NNNNTTATATTTTTTATTTGGAGCATGGTCAGCTATGCTAGGTACTGCTATGTCTTTATTGATTCGTCTTGAATTGAGACAGAGAGGGAGTTTTTTAGGAAATGATCATTTATATAATGTTATTGTTACTGCTCACGCTTTTGTAATGATTTTTTTTATGGTTATGCCAATTATGATTGGAGGTTTTGGTAATTGATTAATTCCTATAATGATTGGGGCGCCAGATATGGCTTTTCCTCGAATAAATAATTTAAGATTCTGGTTGTTGCCCCCTTCTTTAGTTTTGTTAGTTGTTTCTAGAATAGTGGAGATTGGCGTGGGGACTGGATGGACTGTTTATCCTCCCCTAGCTTCGTATGTAGGACATGCTGTTGTTTCTGTGGATTTTGCTATTTTTTCTTTGCATTTGGCTGGTGCCTCATCTATTATGGGGGCTATTAATTTTATTACTACAGTTATCAATATACGAAGAGGTGGTATAACTATAGAGAAAGTTCCGTTATTTGTTTGGAGAGTTTTAATTACAGCGGTTTTATTGTTATTATCTTTACCTGTATTGGCCGGGGCTATTACTATGTTACTTACTGATCGGAATTTTAATACATCATTTTTTGACCCTGCTGGAGGGGGGGATCCAGTATTGTTTCAGCATTTA

>BGE_00513_H08 Barusia cf. maheni

NNNNTTATATTTTATATTTGGGGTTTGATCAGCTATATTGGGAACTGCTATATCTTTGTTAATTCGGCTTGAACTGAGACAGAGTGGAAGTTTTCTTGGAAATGACCATCTATATAATGTTATTGTTACTGCACATGCTTTTGTAATGATTTTTTTTATGGTTATGCCAATTTTAATTGGGGGGTTTGGTAATTGGTTAATCCCTTTAATGATTGGGGCTCCTGATATAGCTTTTCCTCGAATGAATAATTTAAGATTTTGGTTGCTTCCACCTTCTTTAGTTTTGTTAATTATTTCTAGAATAGTGGAGATTGGAGCAGGAACTGGGTGAACTGTGTATCCGCCTTTAGCTTCATATGTTGGGCATGCTCATGTTTCTGTAGACTTTGCTATTTTTTCTTTGCATTTGGCTGGTGCTTCATCTATTATGGGGGCTATTAATTTTATTACTACGGTAATAAACATACGAAGAGGAGGAATGACCATAGATAAGGTTCCTTTATTTGTTTGAAGAGTGTTAATTACAGCGGTTTTATTATTATTGTCTTTGCCTGTGTTGGCTGGGGCTATTACTATGTTGCTTACTGATCGAAATTTTAATACATCATTTTTTGACCCTGCTGGTGGGGGGGATCCAGTATTGTTTCAGCATTTA

>BGE_00513_B12 Sulcia 24761882

NNNNTTGTATTTTCTGTTGGGGGCGTGGTCTGCTATGGTAGGGGCTGCTATGAGTATGTTGATTCGGATTGAGTTGGGGCATCCTGGATCATTCCTTGGGAATGACCATGTTTATAACGTGATTGTAACGGCGCATGCGTTTGTAATGATTTTTTTTATGGTCATGCCTATTTTAATTGGAGGGTTTGGGAATTGGCTGGTGCCTTTAATGATTGGGGCGCCTGATATGGCTTTTCCTCGAATAAATAATTTAAGTTTTTGGTTGTTACCTCCATCAATAAGAATGTTGCTTATTTCTGGGATAGTGGAAATGGGGGTGGGGACGGGATGAACAATTTATCCTCCTTTAGCTTCTTACGTGGGTCATGCTTTTGTTTCTGTTGATTTTGCTATTTTTTCTTTGCATTTGGCTGGGGCTTCGTCTATTATGGGGGCTATTAATTTTATCACCACGGTGTTGAACATGCGGGGTGCTGGAGTGTCGATGGATAGGGTGCCTTTGTTTGTGTGGTCTGTTTTAATTACTGCGGTGTTATTATTATTATCGCTCCCGGTGTTGGCGGGAGCGATCACCATGTTGTTGACAGATCGCAATTTTAACACTTCCTTTTTCGATCCCGCAGGGGGAGGAGATCCGGTTTTATTTCAGCACTTG

>BGE_00513_C03 Sulcia 24761885

NNNNTTGTACTTTCTTTTTGGGGCATGGTCGGCTATGTTAGGGACTGCTATGAGTTTATTGATTCGGGTTGAATTGGGACAGTCTGGAAGTTTCTTTGGAAATGATCATTTGTATAATGTGATTGTGACTGCTCATGCGTTTGTGATGATTTTTTTTATGGTTATGCCTATTTTAATTGGGGGATTTGGAAATTGATTGGTGCCTTTAATGATTGGAGCGCCGGATATGGCTTTTCCTCGAATGAATAATTTAAGTTTTTGGTTGTTACCACCATCGATGACTTTGCTTCTTATTTCGGGGATGGTTGAAATAGGGGTGGGGACGGGGTGAACGATTTATCCCCCTTTGGCTTCTTATGTGGGGCATGCCTTTGTTTCTGTAGATTTTGCTATTTTTTCGTTGCACTTGGCTGGGGCGTCTTCTATTATGGGTGCGATTAATTTTATTACGACGGTGTTAAATATGCGGGGAGCAGGAGTGAGTATGGATAGGGTTCCTTTGTTTGTTTGGTCTGTTTTTATTACTGCAATTTTGTTGTTGTTGTCGTTGCCTGTGCTGGCGGGGGCAATTACTATGTTATTGACGGATCGAAATTTTAATACTTCATTTTTTGATCCTGCGGGGGGGGGGGATCCAGTTTTGTTTCAACATTTG

>BGE_00513_D09 Sulcia 24761903

NNNNTTGTACTTTCTTTTTGGGGCATGGTCGGCTATGTTAGGGACTGCTATGAGTTTGTTGATTCGGGTTGAGTTGGGACAGTCTGGTAGTTTTTTTGGAAATGATCATTTGTACAATGTGATTGTGACTGCTCATGCGTTTGTGATGATTTTTTTTATGGTTATGCCTATTTTAATTGGGGGATTTGGAAATTGGTTAGTACCTTTAATAATTGGGGCGCCGGATATGGCTTTCCCTCGAATGAATAATTTAAGTTTTTGATTGTTACCACCTTCGGTAACATTGCTTCTTATTTCAGGGATGGTTGAAATAGGAGTGGGGACTGGGTGGACGATTTATCCTCCTTTGGCTTCTTATGTGGGGCATGCTTTTGTTTCTGTAGATTTTGCTATTTTTTCTTTGCACTTGGCTGGGGCGTCTTCTATTATGGGTGCAATTAATTTTATTACAACGGTGTTAAATATGCGGGGAGCAGGAGTGAGTATGGATAAGGTTCCTTTGTTTGTTTGGTCTGTTTTTATTACTGCAATTTTGTTGTTGTTATCATTGCCTGTGCTTGCTGGGGCTATTACTATGCTATTAACCGATCGAAATTTTAATACTTCATTTTTTGACCCTGCTGGGGGGGGCGATCCGGTTTTGTTTCAGCATTTG

>BGE_00513_C08 Sulcia 24761890

NNNNTTGTATTTTTTGTTCGGGGCTTGGTCTGCAATGTTAGGGACGGCTATAAGTTTATTGATTCGAATTGAATTAGGCCAGGTGGGGTCATTTTTTGGAAATGACCATTTATATAATGTTGTTGTAACAGCACATGCGTTTGTGATAATTTTTTTTATAGTTATGCCTATTTTAATTGGTGGTTTTGGAAATTGATTAATTCCTTTGATGATTGGGGCTCCTGATATAGCTTTTCCTCGAATGAATAATTTAAGTTTTTGATTATTGCCTCCTTCATTATCTTTGCTTCTTATTTCTGGAATGGTGGAAATGGGAGTGGGAACTGGGTGGACTATTTATCCTCCGTTGGCTTCTTATGTAGGGCATGCCTTTGTTTCTGTGGATTTTGCTATTTTTTCTTTACATTTGGCGGGAGCTTCTTCTATCATGGGAGCTATTAATTTTATTACTACCGTAGTGAACATGCGGGGTGCAGGATTAACCATGGATAAGGTTCCTTTATTTGTATGATCTGTTTTGATTACTGCTGTGTTGTTACTTTTATCTTTGCCTGTGTTGGCTGGTGCAATTACAATGTTGTTGACGGATCGTAATTTTAATACTTCGTTTTTTGATCCTGCGGGAGGGGGAGATCCTGTTTTATTTCAACATTTG

>BGE_00521_A01 Sulcia 26742975

NNNNTTGTATTTTTTGTTTGGGGCTTGGTCTGCAATGTTAGGGACGGCTATGAGTTTATTGATTCGAATTGAGTTAGGTCAGGTAGGATCATTTCTTGGAAATGATCATTTATATAATGTTATTGTGACGGCACATGCATTTGTGATAATTTTTTTTATGGTTATGCCTATTTTGATTGGTGGTTTTGGAAATTGATTGATTCCTTTGATGATTGGGGCTCCTGATATAGCTTTTCCTCGAATGAATAACTTAAGTTTTTGGTTATTGCCTCCTTCATTATCTTTGCTTCTTATTTCTGGAATGGTGGAAATGGGAGTGGGAACTGGGTGGACTATTTACCCTCCGTTGGCTTCTTATGTGGGGCATGCCTTTGTTTCTGTAGATTTTGCTATTTTTTCTTTACATTTAGCTGGAGCTTCTTCTATTATGGGGGCAATTAATTTTATTACTACTGTAGTAAACATACGAGGTGCGGGAATAACTATGGATAAGGTTCCTTTATTTGTATGATCTGTTTTGGTTACTGCTGTGTTATTACTTTTGTCCTTGCCTGTGTTGGCTGGTGCGATTACAATGTTGTTGACAGATCGTAATTTTAATACTTCGTTTTTTGACCCTGCAGGGGGGGGGGATCCTGTTTTATTTCAACATTTG

>BGE_00521_D09 Sulcia 26743019

NNNNTTGTATTTTTTGTTTGGGGCTTGATCTGCAATGTTAGGGACAGCTATGAGTTTATTGATTCGAATTGAGTTAGGTCAGGTAGGATCATTTCTTGGAAATGATCATTTATATAATGTTATTGTGACGGCACATGCATTTGTGATAATTTTTTTTATGGTTATGCCTATTTTGATTGGTGGTTTTGGGAATTGATTGATTCCTTTGATGATTGGAGCTCCTGATATAGCTTTTCCTCGAATGAATAACTTAAGTTTTTGGTTATTGCCTCCTTCATTATCTTTGCTTCTTATTTCTGGAATGGTGGAGATGGGAGTGGGAACTGGGTGGACTATTTACCCTCCGTTGGCTTCTTATGTGGGGCATGCCTTTGTTTCTGTAGATTTTGCTATTTTTTCTTTACATTTGGCTGGAGCTTCTTCTATTATGGGGGCAATTAATTTTATTACTACTGTAGTAAACATACGAGGTGCGGGAATAACTATGGATAAGGTTCCTTTATTTGTATGATCTGTTTTGATTACTGCTGTGTTATTACTTTTGTCCTTGCCTGTGTTGGCTGGTGCGATTACAATGTTGTTGACAGATCGTAATTTTAATACTTCGTTTTTTGACCCTGCCGGGGGGGGGGATCCTGTTTTATTTCAACATTTG

>BGE_00521_B04 Sulcia 26742990

NNNNTTGTATTTTTTGTTTGGGGCTTGGTCTGCAATGTTAGGGACGGCTATGAGTTTATTGATTCGAATTGAGTTAGGTCAGGTAGGATCATTTCTTGGAAATGATCATTTATATAATGTTATTGTGACGGCACATGCATTTGTGATAATTTTTTTTATGGTTATGCCTATTTTGATTGGTGGTTTTGGAAATTGATTGATTCCTTTGATGATTGGGGCTCCTGATATAGCTTTTCCTCGAATGAATAACTTAAGTTTTTGGTTATTACCTCCTTCATTATCTTTGCTTCTTATTTCTGGAATGGTGGAAATGGGAGTGGGAACTGGGTGGACTATTTACCCTCCGTTGGCTTCTTATGTGGGGCATGCCTTTGTTTCTGTAGATTTTGCTATTTTTTCTTTACATTTGGCTGGAGCTTCTTCTATTATGGGGGCAATTAATTTTATTACTACTGTGGTAAACATACGAGGTGCGGGAATAACTATGGATAAGGTTCCTTTATTTGTATGGTCTGTTTTGATTACTGCTGTGTTATTACTTTTGTCTTTGCCTGTGTTGGCTGGTGCGATTACAATGTTGTTAACAGATCGTAATTTTAATACTTCGTTTTTTGATCCTGCAGGAGGGGGGGATCCTGTTTTATTTCAACATTTG

>BGE_00521_C11 Sulcia 26743009

NNNNTTGTATTTTTTGTTTGGGGCTTGGTCTGCAATGTTAGGGACGGCTATGAGTTTATTGATTCGAATTGAGTTAGGTCAGGTAGGATCATTTCTTGGAAATGATCATTTATATAATGTTATTGTGACGGCACATGCATTTGTGATAATTTTTTTTATGGTTATGCCTATTTTGATTGGTGGTTTTGGAAATTGATTGATTCCTTTGATGATTGGGGCTCCTGATATAGCTTTTCCTCGAATGAATAACTTAAGTTTTTGGTTATTACCTCCTTCATTATCTTTGCTTCTTATTTCTGGAATGGTGGAAATGGGAGTGGGAACTGGGTGGACTATTTACCCTCCGTTGGCTTCTTATGTGGGGCATGCCTTTGTTTCTGTAGATTTTGCTATTTTTTCTTTACATTTGGCTGGAGCTTCTTCTATTATGGGGGCAATTAATTTTATTACTACTGTGGTAAACATACGAGGTGCGGGAATAACTATGGATAAGGTTCCTTTATTTGTATGGTCTGTTTTGATTACTGCTGTGTTATTACTTTTGTCTTTGCCTGTGTTGGCTGGTGCGATTACAATGTTGTTAACAGATCGTAATTTTAATACTTCGTTTTTTGATCCTGCAGGAGGGGGGGATCCTGTTTTATTTCAACATTTG

>BGE_00521_E05 Sulcia 26743027

NNNNTTGTATTTTTTGTTTGGGGCTTGGTCTGCAATGTTAGGGACGGCTATGAGTTTATTGATTCGAATTGAGTTAGGTCAGGTAGGATCATTTCTTGGAAATGATCATTTATATAATGTTATTGTGACGGCACATGCATTTGTGATAATTTTTTTTATGGTTATGCCTATTTTGATTGGTGGTTTTGGAAATTGATTGATTCCTTTGATGATTGGGGCTCCTGATATAGCTTTTCCTCGAATGAATAACTTAAGTTTTTGGTTATTACCTCCTTCATTATCTTTGCTTCTTATTTCTGGAATGGTGGAAATGGGAGTGGGAACTGGGTGGACTATTTACCCTCCGTTGGCTTCTTATGTGGGGCATGCCTTTGTTTCTGTAGATTTTGCTATTTTTTCTTTACATTTGGCTGGAGCTTCTTCTATTATGGGGGCAATTAATTTTATTACTACTGTGGTAAACATACGAGGTGCGGGAATAACTATGGATAAGGTTCCTTTATTTGTATGGTCTGTTTTGATTACTGCTGTGTTATTACTTTTGTCTTTGCCTGTGTTGGCTGGTGCGATTACAATGTTGTTAACAGATCGTAATTTTAATACTTCGTTTTTTGATCCTGCAGGAGGGGGGGATCCTGTTTTATTTCAACATTTG

>BGE_00521_E08 Sulcia 26743030

NNNNTTGTATTTTTTGTTTGGGGCTTGGTCTGCAATGTTAGGGACGGCTATGAGTTTATTGATTCGAATTGAGTTAGGTCAGGTAGGATCATTTCTTGGAAATGATCATTTATATAATGTTATTGTGACGGCACATGCATTTGTGATAATTTTTTTTATGGTTATGCCTATTTTGATTGGTGGTTTTGGAAATTGATTGATTCCTTTGATGATTGGGGCTCCTGATATAGCTTTTCCTCGAATGAATAACTTAAGTTTTTGGTTATTACCTCCTTCATTATCTTTGCTTCTTATTTCTGGAATGGTGGAAATGGGAGTGGGAACTGGGTGGACTATTTACCCTCCGTTGGCTTCTTATGTGGGGCATGCCTTTGTTTCTGTAGATTTTGCTATTTTTTCTTTACATTTGGCTGGAGCTTCTTCTATTATGGGGGCAATTAATTTTATTACTACTGTGGTAAACATACGAGGTGCGGGAATAACTATGGATAAGGTTCCTTTATTTGTATGGTCTGTTTTGATTACTGCTGTGTTATTACTTTTGTCTTTGCCTGTGTTGGCTGGTGCGATTACAATGTTGTTAACAGATCGTAATTTTAATACTTCGTTTTTTGATCCTGCAGGAGGGGGGGATCCTGTTTTATTTCAACATTTG

>BGE_00521_E09 Sulcia 26743031

NNNNTTGTATTTTTTGTTTGGGGCTTGGTCTGCAATGTTAGGGACGGCTATGAGTTTATTGATTCGAATTGAGTTAGGTCAGGTAGGATCATTTCTTGGAAATGATCATTTATATAATGTTATTGTGACGGCACATGCATTTGTGATAATTTTTTTTATGGTTATGCCTATTTTGATTGGTGGTTTTGGAAATTGATTGATTCCTTTGATGATTGGGGCTCCTGATATAGCTTTTCCTCGAATGAATAACTTAAGTTTTTGGTTATTACCTCCTTCATTATCTTTGCTTCTTATTTCTGGAATGGTGGAAATGGGAGTGGGAACTGGGTGGACTATTTACCCTCCGTTGGCTTCTTATGTGGGGCATGCCTTTGTTTCTGTAGATTTTGCTATTTTTTCTTTACATTTGGCTGGAGCTTCTTCTATTATGGGGGCAATTAATTTTATTACTACTGTGGTAAACATACGAGGTGCGGGAATAACTATGGATAAGGTTCCTTTATTTGTATGGTCTGTTTTGATTACTGCTGTGTTATTACTTTTGTCTTTGCCTGTGTTGGCTGGTGCGATTACAATGTTGTTAACAGATCGTAATTTTAATACTTCGTTTTTTGATCCTGCAGGAGGGGGGGATCCTGTTTTATTTCAACATTTG

>BGE_00521_C09 Sulcia 26743007

NNNNTTGTATTTTTTGTTTGGGGCTTGGTCTGCAATGTTAGGGACGGCTATAAGTTTGTTGATTCGAATTGAGTTAGGTCAGGTGGGATCATTTCTTGGAAATGATCATTTATATAATGTTATTGTGACGGCACATGCATTTGTGATAATTTTTTTTATGGTTATGCCTATTTTGATTGGGGGTTTTGGAAATTGATTAATTCCTTTAATGATTGGGGCTCCTGATATAGCTTTTCCTCGGATGAATAATTTAAGTTTTTGGTTATTGCCTCCTTCATTATTTTTGCTTCTTGTTTCTGGAATGGTGGAAATGGGAGTGGGAACTGGGTGGACTATTTATCCTCCGTTGGCTTCTTATGTGGGGCATGCCTTTGTTTCTGTAGATTTTGCTATTTTTTCTTTACATTTGGCTGGAGCTTCTTCTATTATGGGGGCAATTAATTTTATTACTACTGTAGTAAACATGCGAGGTGCGGGAATGACCATGGATAAGGTTCCTTTATTTGTATGATCTGTTTTGATTACTGCTGTGTTATTACTTTTGTCCTTGCCTGTGTTGGCTGGTGCGATTACAATGTTGTTGACAGATCGTAATTTTAATACCTCGTTTTTTGACCCTGCAGGGGGAGGGGATCCTGTTTTATTTCAACATTTG

>BGE_00521_B06 Sulcia 26742992

NNNATTGTATTTTTTGTTCGGAGCTTGGTCTGCAATGTTAGGGACCGCAATGAGTTTATTGATTCGAATTGAGTTAGGTCAGGTAGGATCATTTCTTGGAAATGATCATTTGTATAATGTTATTGTAACAGCACATGCGTTTGTGATAATTTTTTTTATGGTTATGCCTATTTTGATTGGGGGTTTTGGAAATTGGTTGATTCCTTTGATGATTGGGGCTCCTGATATGGCTTTTCCTCGAATGAATAACTTAAGTTTTTGATTATTACCTCCCTCATTATCTTTGCTTCTTATTTCTGGAATGGTGGAAATGGGGGTGGGGACTGGGTGGACCATTTATCCTCCGTTGGCTTCTTATGTAGGGCATGCCTTTGTTTCTGTGGATTTTGCTATTTTTTCTTTACATTTGGCCGGGGCTTCTTCTATTATGGGGGCGATTAATTTTATTACTACTGTAGTAAATATGCGGAGTGCGGGAATAACCATGGATAAGGTTCCTTTATTTGTATGATCTGTTTTGATTACTGCTGTGTTATTGCTTTTATCCTTGCCTGTTTTAGCTGGGGCGATTACAATGTTGTTGACAGATCGTAATTTTAATACTTCGTTTTTTGACCCTGCAGGGGGGGGGGACCCTGTTTTATTTCAACATTTG

>BGE_00521_C05 Sulcia 26743003

NNNATTGTATTTTTTGTTCGGAGCTTGGTCTGCAATGTTAGGGACCGCAATGAGTTTATTGATTCGAATTGAGTTAGGTCAGGTAGGATCATTTCTTGGAAATGATCATTTGTATAATGTTATTGTAACAGCACATGCGTTTGTGATAATTTTTTTTATGGTTATGCCTATTTTGATTGGGGGTTTTGGAAATTGGTTGATTCCTTTGATGATTGGGGCTCCTGATATGGCTTTTCCTCGAATGAATAACTTAAGTTTTTGATTATTACCTCCCTCATTATCTTTGCTTCTTATTTCTGGAATGGTGGAAATGGGGGTGGGGACTGGGTGGACCATTTATCCTCCGTTGGCTTCTTATGTAGGGCATGCCTTTGTTTCTGTGGATTTTGCTATTTTTTCTTTACATTTGGCCGGAGCTTCTTCTATTATGGGGGCGATTAATTTTATTACTACTGTAGTAAATATGCGGAGTGCGGGAATAACTATGGATAAGGTTCCTTTATTTGTATGATCTGTTTTGATTACTGCTGTGTTATTGCTTTTATCCTTGCCTGTTTTAGCTGGGGCGATTACAATGTTGTTGACAGATCGTAATTTTAATACTTCGTTTTTTGACCCTGCAGGCGGGGGGGACCCTGTTTTATTTCAACATTTG

>BGE_00513_D08 Sulcia 24761902

NNNNTTATATTTTTTGTTTGGAGCTTGATCTGCTATATTAGGGACGGCTATAAGTTTATTGATTCGAATTGAATTAGGTCATGTGGGATCGTTTCTTGGAAATGATCATTTATATAATGTTATTGTAACGGCTCATGCGTTTGTGATAATTTTTTTTATGGTTATACCTATTTTGATTGGTGGTTTTGGAAATTGATTAATTCCTTTAATGATTGGGGCTCCTGATATAGCTTTTCCTCGTATAAATAATTTAAGTTTTTGATTGTTACCCCCTTCATTATCTTTGCTTCTTATTTCTGGAATGGTAGAAATAGGGGTGGGAACTGGATGGACTATTTATCCTCCATTGGCTTCTTATGTAGGGCATGCCTTTGTTTCTGTGGATTTTGCTATTTTTTCTTTGCATTTGGCTGGAGCTTCTTCTATTATGGGGGCAATTAATTTTATTACTACAGTGGTAAATATGCGAGGTGCTGGGATAACTATGGATAAGGTCCCTTTATTTGTGTGGTCTGTTTTAATTACTGCTGTGTTGTTACTTTTGTCTTTGCCTGTGTTGGCTGGAGCAATTACAATATTGTTAACGGATCGTAATTTTAATACTTCGTTTTTTGATCCTGCAGGAGGAGGAGATCCTGTTTTATTTCAACATTTG

>BGE_00521_D05 Sulcia 26743015

NNNNTTGTATTTTTTGTTTGGGGCTTGATCTGCGATGTTAGGGACGGCTATAAGTCTATTGATTCGAATTGAATTAGGTCAGGTTGGGTCATTTCTTGGTAATGACCATTTGTATAATGTTATTGTGACAGCTCATGCGTTTGTCATGATTTTTTTTATGGTTATGCCTATTTTGATTGGTGGTTTTGGAAATTGATTAATTCCTTTAATGATTGGAGCTCCTGATATAGCTTTTCCTCGTATGAATAATTTAAGTTTTTGGTTATTACCTCCTTCATTATCTTTGCTTCTTATTTCTGGAATGGTTGAGATAGGGGTAGGGACTGGGTGGACTATTTATCCTCCTTTAGCTTCTTATGTGGGGCATGCCTTTGTTTCTGTGGATTTTGCTATTTTTTCTTTACATTTGGCTGGAGCTTCTTCTATTATGGGGGCGATTAATTTTATTACTACTGTGGTAAATATGCGAGGTGCTGGGATGACTATAGATAAGGTTCCTTTATTTGTGTGATCTGTTTTAGTTACTGCTGTGTTATTGCTCTTGTCTTTACCTGTGTTGGCTGGGGCCATTACAATATTATTGACAGATCGTAATTTTAATACTTCATTTTTTGATCCCGCAGGAGGAGGGGATCCTGTGTTATTCCAGCATTTG

>BGE_00513_G02 Sulcia 24761932

NNNGATGTATCTCTTGTTTGGGGCTTGATCTGCGATGTTAGGAACGGCTATGAGTTTGTTGATCCGAATTGAATTGGGACAGGTGGGGTCATTTCTTGGGAATGACCATTTGTACAATGTTATTGTGACGGCTCACGCGTTTGTCATAATTTTTTTTATGGTAATGCCTATTTTGATTGGTGGTTTTGGAAATTGGTTAATTCCTTTAATGATTGGGGCTCCTGACATAGCCTTTCCTCGTATGAATAATTTAAGTTTTTGGTTGTTGCCTCCTTCATTGTTTTTGCTTCTTATCTCTGGAATGGTAGAAATGGGGGTGGGGACAGGATGAACAATTTATCCTCCTTTGGCTTCTTATGTAGGGCATNNNNNNNNNTCTGTGGATTTTGCAATTTTTTCTTTACATTTGGCTGGGGCTTCTTCTATTATGGGGGCGATTAATTTTATTACTACTGTGTTGAACATGCGAGGTGCTGGGTTGTCCATAGATAAGGTTCCTTTATTTGTGTGATCTGTGTTAATTACTGCTGTGTTGTTATTATTGTCTTTGCCTGTGTTGGCTGGGGCAATTACAATGTTGTTGACAGATCGTAATTTTAATACTTCGTTTTTTGATCCTGCAGGAGGGGGGGATCCTGTTTTATTTCAACATTTG

>BGE_00513_G04 Sulcia 24761934

NNNNTTGTATTTTTTGTTTGGGGCTTGATCTGCGATGTTAGGAACGGCTATGAGTTTGTTGATCCGAATTGAATTGGGACAGGTGGGGTCATTTCTTGGGAATGACCATTTGTACAATGTTATTGTGACGGCTCACGCGTTTGTCATAATTTTTTTTATGGTAATGCCTATTTTGATTGGTGGTTTTGGAAATTGGTTAATTCCTTTAATGATTGGGGCTCCTGACATAGCCTTTCCTCGTATGAATAATTTAAGTTTTTGGTTGTTGCCTCCTTCATTGTTTTTGCTTCTTATCTCTGGAATGGTAGAAATGGGGGTGGGGACAGGATGAACAATTTATCCTCCTTTGGCTTCTTATGTAGGGCATGCCTTTGTTTCTGTGGATTTTGCAATTTTTTCTTTACATTTGGCTGGGGCTTCTTCTATTATGGGGGCGATTAATTTTATTACTACTGTGTTGAACATGCGAGGTGCTGGGTTGTCCATAGATAAGGTTCCTTTATTTGTGTGATCTGTGTTAATTACTGCTGTGTTGTTATTATTGTCTTTGCCTGTGTTGGCTGGGGCAATTACAATGTTGTTGACAGATCGTAATTTTAATACTTCGTTTTTTGATCCTGCAGGAGGGGGGGATCCTGTTTTATTTCAACATTTG

>BGE_00521_A11 Sulcia 26742985

NNNNTTGTATTTTTTGTTTGGAGCATGATCTGCGATGTTAGGGACGGCGATGAGTTTGTTGATTCGAGTTGAGTTGGGTCAAGTAGGATCATTTTTTGGGAATGATCATTTGTATAATGTGGTTGTGACGGCGCATGCGTTTGTAATGATTTTTTTTATGGTTATGCCTATTTTAATTGGTGGTTTTGGAAACTGGTTAATTCCTTTAATGATTGGGGCACCTGATATAGCTTTTCCTCGTATGAATAATTTGAGTTTTTGATTGTTGCCTCCTTCTTTATCGATGTTGCTTGTTTCTGGCATAGTGGAAATAGGTGTGGGAACAGGATGAACAATTTATCCGCCACTAGCTTCTTATGTGGGGCATGCCTTTGTTTCTGTGGATTTTGCTATTTTTTCATTGCATCTGGCTGGGGCCTCTTCTATCATAGGGGCTATCAATTTTATTACAACTGTGGTCAACATGCGAGGTGCGGGGATGTCTATAGATAAGGTGCCCTTGTTTGTGTGGTCTGTTTTAATTACTGCGGTGTTGTTGTTGTTATCTTTGCCGGTGTTGGCTGGAGCAATCACCATACTATTAACGGATCGTAATTTTAATACTTCGTTTTTTGATCCTGCTGGGGGGGGAGATCCTGTTTTATTTCAACATTTG

>BGE_00521_E07 Sulcia 26743029

NNNNTTGTATTTTTTATTTGGGGCATGGTCTGCAATGTTAGGGACGGCTATAAGTTTGTTGATTCGGATTGAGTTAGGTCAAGTGGGATCATTTTTTGGAAATGATCACTTGTATAATGTGGTTGTAACAGCTCATGCATTTGTAATGATTTTTTTTATGGTTATGCCAATTTTGATCGGTGGTTTTGGAAACTGGCTGATTCCTTTAATGATTGGAGCACCTGATATGGCTTTCCCTCGTATGAATAATTTGAGTTTTTGATTACTACCCCCTTCAATATTGATGTTACTTATTTCTGGCATAGTAGAGATGGGTGTGGGGACAGGATGAACAATTTACCCCCCCCTTGCTTCTTATGTGGGGCATGCTTTTGTTTCTGTGGATTTTGCTATCTTTTCTTTGCATTTGGCTGGGGCTTCTTCTATTATGGGGGCAATTAATTTTATTACAACTGTGGTTAACATGCGGGGCGCGGGAATGTCCATAGATAAAGTGCCTTTGTTTGTGTGGTCTGTTTTAATCACTGCGGTATTGTTATTGTTATCTTTACCGGTGTTGGCTGGAGCGATTACTATATTGTTAACAGATCGCAATTTTAATACTTCGTTTTTTGACCCTGCTGGAGGGGGAGATCCTGTTTTATTTCAACATTTG

>BGE_00521_F03 Sulcia 26743037

NNNNTTGTATTTTTTATTTGGGGCATGGTCTGCGATGTTAGGGACGGCTATAAGTTTGTTGATTCGGATTGAGTTAGGTCAAGTGGGATCATTTTTTGGAAATGATCACTTGTATAATGTGGTTGTGACAGCTCATGCGTTTGTAATGATTTTTTTTATGGTTATGCCAATTTTGATCGGTGGTTTTGGAAACTGGCTGATTCCTTTAATGATTGGAGCACCTGATATGGCTTTCCCTCGTATGAATAATTTGAGTTTTTGATTACTACCCCCTTCAATATTGATGTTACTTATTTCTGGCATAGTAGAGATGGGTGTGGGGACAGGATGAACAATTTACCCTCCCCTTGCTTCTTATGTGGGGCATGCCTTTGTTTCTGTGGATTTTGCTATTTTTTCTTTGCATTTGGCTGGGGCTTCTTCTATTATGGGAGCAATTAATTTTATTACAACTGTGGTTAACATGCGGGGCGCGGGAATGTCCATAGATAAAGTGCCTTTGTTTGTGTGGTCTGTTTTAATCACTGCGGTATTATTATTGTTATCTTTACCGGTGTTGGCTGGAGCGATTACTATATTGTTAACAGATCGCAATTTTAATACTTCGTTTTTTGACCCTGCTGGAGGGGGAGATCCTGTTTTATTTCAACATTTG

>BSCRO41624 Cataleptoneta_lingulata

NNNNNNNNNNNNNNNNNNNNNNNNNNNNNNNNNNNNNNNNNNNNNNNNNNNNNNNNNNNNNNNNNNNNNNNNNNNNNNNNNNNNNNNNNNNNNNNNNNNNNNNNNNNNNNNNNNNNNNNNNNNNNNNNNNNNNNNNNNNNNNNNNNNNNNNNNNNNNNNNNNNNNNNNNNNNNNNNNNNNNNNNNNNNNNNNNNNNNNNNNNNNNNNNNNNNNNNNNNNNNNNNNNNNNNNNNNNNNNCGAATAAATAATTTAAGTTTTTGATTATTACCACCTTCGTTAATTTTGTTGATTATTTCTAGTATGGTAGAAATTGGTGTAGGTACTGGTTGAACTGTTTACCCTCCTTTAGCTTCTTATGTTGGGCATNNNNNNNNNNNNGTAGATTATGCTATTTTTTCATTACATTTAGCTGGTGCTTCGTCTATTATAGGTGCAGTTAATTTTATTACTACTGTAGTAAATATACGTAGTGGTGGAATAACTATGGAAAAAACGCCTTTATTTGTATGGTCTGTTTTAATTACTGCGGTGTTGTTATTATTGTCTTTACCCGTGTTGGCAGGTGCTATTACTATGTTATTGACGGATCGAAATTTTAATACTTCGTTTTTTGATCCTGCTGGTGGAGGGGATCCTGTTTTATTTCAACATTTN

>BSUIO106025 Cataleptoneta_detriticola

NNNNNNNNNNNNNNNNNNNNNNNNNNNNNNNNNNNNNNNNNNNNNNNNNNNNNNNNNNNNNNNNNNNNNNNNNNNNNNNNNNNNNNNNNNNNNNNNNNNNNNNNNNNNNNNNNNNNNNNNNNNNNNNNNNNNNNNNNNNNNNNNNNNNNNNNNNNNNNNNNNNNNNNNNNNNNNNNNNNNNNNNNNNNNNNNNNNNNNNNNNNNNNNNNNNNNNNNNNNNNNNNNNNNNNNNNNNNNNCGTATGAATAACTTGAGATTTTGGTTATTGCCTCCTTCTTTGTTATTATTAATTGTTTCTGGAATGGTAGAATTGGGAGTGGGGACGGGTTGGACTGTCTATCCTCCTTTGGCTTCTNNNNNNNNNNNNNNNNNNNNNTCTGTGGATTTTGCTATTTTTTCTTTACATTTGGCAGGGGCATCATCTATTATGGGGGCTATTAATTTTATTACTACTATTNNNNNNNNNNNNNNNNNNNNNNNNNNNNNNGAAAAAGTACCTTTGTTTGTATGGTCTGTATTGGTTACAGCAGTTTTATTATTGTTGTCGTTGCCTGTTTTAGCAGGAGCAATTACAATGTTATTAACTGATCGTAATTTTAATACTTCTTTTTTTGATCCTGCCGGGGGTGGAGATCCAGTATTGTTTCAACATTTN

>JN378155 Leptoneta_microphthalma

NNNNNNNNNNNNNNNNNNNNNNNNNNNNNNNNNNNNNNNNNNNNNNNNNNNNNNNNNNNNNNNNNNNNNNNNNNNNNNNNNNNNNNNNNNNNNNNNNNNNNNNNNNNNNNNNNNNNNNNNNNNNNNNNNNNNNNNNNNNNNNNNNNNNNNNNNNNNNNNNNNNNNNNNNNNNNNNNNNNNNNNNNNNNNNNNNNNNNNNNNNNNNNNNNNNNNNNNNNNNNNNNNNNNNNNNNNNNNNCGTATGAATAATTTGAGTTTTTGGTTGTTACCTCCTTCTTTATTTTTGTTGGTGGTTTCTAGAATAGTTGAAATAGGGGTTGGAACAGGTTGAACTGTATATCCTCCATTATCTTCTAATGTTGGACATGCTTTTGTGTCGGTTGATTTTGCTATTTTTTCTCTTCATTTGGCTGGAGCTTCTTCTATTATGGGGGCTGTAAATTTTATTACTACTATTGTAAATATGCGAACTTTTGGTATAACTATAGAAAAAGTTCCTTTGTTTGTTTGATCAGTGTTAGTGACTGCTGTTCTGTTGTTGCTTTCTTTACCTGTGTTAGCAGGTGCCATTACTATGTTGTTAACTGATCGTAATTTTAATACATCTTTTTTTGATCCTGCTGGTGGGGGGGATCCAGTGTTATTTCAACACTTN

>JN378156 Leptoneta convexa voucher 9040712

NNNNNNNNNNNNNNNNNNNNNNNNNNNNNNNNNNNNNNNNNNNNNNNNNNNNNNNNNNNNNNNNNNNNNNNNNNNNNNNNNNNNNNNNNNNNNNNNNNNNNNNNNNNNNNNNNNNNNNNNNNNNNNNNNNNNNNNNNNNNNNNNNNNNNNNNNNNNNNNNNNNNNNNNNNNNNNNNNNNNNNNNNNNNNNNNNNNNNNNNNNNNNNNNNNNNNNNNNNNNNNNNNNNNNNNNNNNNNTCGTATGAATAATTTGAGTTTTTGGTTATTACCCCCTTCTTTATTTTTATTAGTGGTTTCTAGAATAGTTGAAATAGGAGTAGGAACAGGTTGGACTGTGTATCCTCCATTGTCTTCTAATGTAGGGCATGCTTTTGTGTCGGTTGATTTTGCTATTTTTTCTCTTCATTTAGCTGGGGCTTCTTCTATTATAGGGGCTGTTAATTTCATTACTACTATTATTAATATGCGAACTTTTGGTATGAGTATGGAAAAGGTTCCCTTGTTTGTTTGATCAGTGTTAGTGACTGCTGTTTTGTTGTTGTTATCTTTACCTGTATTAGCTGGTGCTATTACTATGTTGTTAACTGATCGTAATTTTAATACCTCTTTTTTTGACCCTGCTGGCGGGGGGGATCCAGTGTTATTTCAACATTTG

>MT607793 Leptoneta_paroculus

NNNNNNNNNNNNNNNNNNNNNNNNNNNNNNNNNNNNNNNNNNNNNNNNNNNNNNNNNNNNNNNNNNNNNNNNNNNNNNNNNNNNNNNNNNNNNNNNNNNNNNNNNNNNNNNNNNNNNNNNNNNNNNNNNNNNNNNNNNNNNNNNNNNNNNNNNNNNNNNNNNNNNNNNNNNNNNNNNNNNNNNNNNNNNNNNNNNNNNNNNNNNNNNNNNNNNNNNNNNNNNNNNNNNNNNNNNNNNNCGAATGAATAATTTAAGTTTTTGATTGTTGCCTCCTTCTTTATTTTTATTGGTGGTTTCTAGAATAGTTGAAATAGGGGTGGGTACAGGATGAACTGTGTATCCTCCCTTGTCTTCTAATGTAGGGCATGCTTTTGTTTCTGTAGATTTTGCTATTTTTTCTCTTCATTTGGCGGGGGCTTCTTCTATTATGGGTGCTGTTAATTTTATTACTACTATTGTAAATATGCGAACTTTTGGAATGACAATGGAAAAAGTTCCATTGTTTGTGTGATCAGTGTTAGTTACTGCTGTTTTATTGTTGTTATCTTTACCAGTATTAGCTGGAGCTATTACTATATTATTAACAGATCGTAATTTTAATACTTCTTTTTTTGACCCCGCTGGGGGCGGGGATCCGGTATTGTTTCAACATTTN

>MW997984 Leptoneta_paroculus

NNNNNNNNNNNNNNNNNNNNNNNNNNNNNNNNNNNNNNNNNNNNNNNNNNNNNNNNNNNNNNNNNNNNNNNNNNNNNNNNNNNNNNNNNNNNNNNNNNNNNNNNNNNNNNNNNNNNNNNNNNNNNNNNNNNNNNNNNNNNNNNNNNNNNNNNNNNNNNNNNNNNNNNNNNNNNNNNNNNNNNNNNNNNNNNNNNNNNNNNNNNNNNNNNNNNNNNNNNNNNNNNNNNNNNNNNNNNNNCGAATGAATAATTTAAGTTTTTGATTGTTGCCTCCTTCTTTATTTTTATTGGTGGTTTCTAGAATAGTTGAAATAGGGGTGGGTACAGGATGAACTGTGTATCCTCCCTTGTCTTCTAATGTAGGGCATGCTTTTGTTTCTGTAGATTTTGCTATTTTTTCTCTTCATTTGGCGGGGGCTTCTTCTATTATGGGTGCTGTTAATTTTATTACTACTATTGTAAATATGCGAACTTTTGGAATGACAATGGAAAAAGTTCCATTGTTTGTGTGATCAGTGTTAGTTACTGCTGTTTTATTGTTGTTATCTTTACCAGTATTAGCTGGAGCTATTACTATATTATTAACAGATCGTAATTTTAATACTTCTTTTTTTGACCCCGCTGGGGGCGGGGATCCGGTATTGTTTCAACATTTN

>KY017764 Leptoneta_infuscata

NNNNNNNNNNNNNNNNNNNNNNNNNNNNNNNNNNNNNNNNNNNNNNNNNNNNNNNNNNNNNNNNNNNNNNNNNNNNNNNNNNNNNNNNNNNNNNNNNNNNNNNNNNNNNNNNNNNNNNNNNNNNNNNNNNNNNNNNNNNNNNNNNNNNNNNNNNNNNNNNNNNNNNNNNNNNNNNNNNNNNNNNNNNNNNNNNNNNNNNNNNNNNNNNNNNNNNNNNNNNNNNNNNNNNNNNNNNNNNCGTATAAATAATTTAAGATTTTGGTTGTTGCCACCTTCTTTATTTTTGTTAATTGTTTCTAGTATAGTAGAAATAGGGGTGGGAACTGGTTGGACTGTTTATCCCCCTTTAGCTTCTAATGTTGGTCATGCTTTTGTATCTGTAGATTTTGCTATTTTTTCTCTTCATTTGGCTGGAGCTTCTTCTATTATGGGGGCTATTAATTTTATTACTACTATTATGAATATGCGTACTTTTGGGATGAGAATGGAGAAGGTTCCATTATTTGTTTGGTCGGTATTAGTAACTGCTATTTTGTTATTATTGTCTTTACCTGTGTTAGCAGGTGCTATTACTATATTGCTAACAGATCGAAACTTTAATACTTCTTTTTTTGATCCTGCTGGAGGGGGAGATCCAGTTTTATTTCAACATTTN

>MT98795 Leptonetela_caucasica

NNNNNNNNNNNNNNNNNNNNNNNNNNNNNNNNNNNNNNNNNNNNNNNNNNNNNNNNNNNNNNNNNNNNNNNNNNNNNNNNNNNNNNNNNNNNNNNNNNNNNNNNNNNNNNNNNNNNNNNNNNNNNNNNNNNNNNNNNNNNNNNNNNNNNNNNNNNNNNNNNNNNNNNNNNNNNNNNNNNNNNNNNNNNNNNNNNNNNNNNNNNNNNNNNNNNNNNNNNNNNNNNNNNNNNNNNNNNNNCGAATAAATAATTTGAGATTTTGGTTATTGCCACCCTCTTTATTTTTATTGGTAGTTTCTGGTATGGTAGAGATAGGGGTGGGCACTGGTTGAACTATTTATCCTCCTTTGGCTGCTTATTTGGGGCATTCTTTTGTGTCAGTGGATTATGCTATTTTTTCTTTACATTTAGCTGGTGCTTCTTCTATTATGGGGGCTGTTAATTTTATTACTACTATTTTTAATATGCGTATAAATGGGATGAGAATGGAGAAGATGCCATTATTTGTTTGGTCAGTATTAATTACAGCAGTTTTATTATTATTGTCACTTCCAGTATTGGCTGGGGCCATTACTATGTTGCTAACAGATCGAAATTTTAATACTTCTTTTTTTGATCCGGCAGGTGGGGGAGATCCTGTTTTATTTCAGCATTTN

>MG573884 Leptonetela_paragamiani

NNNNNNNNNNNNNNNNNNNNNNNNNNNNNNNNNNNNNNNNNNNNNNNNNNNNNNNNNNNNNNNNNNNNNNNNNNNNNNNNNNNNNNNNNNNNNNNNNNNNNNNNNNNNNNNNNNNNNNNNNNNNNNNNNNNNNNNNNNNNNNNNNNNNNNNNNNNNNNNNNNNNNNNNNNNNNNNNNNNNNNNNNNNNNNNNNNNNNNNNNNNNNNNNNNNNNNNNNNNNNNNNNNNNNNNNNNNNNNCGAATGAATAATTTGAGTTTTTGATTGTTACCTCCATCTTTGTTTTTATTGGTAGTTTCTGGGATGGTAGAGGTAGGGGTTGGGACGGGATGAACTATTTATCCTCCTTTGGCTGCATATGTGGGACATTCTTTTGTGTCGGTGGATTATGCTATTTTTTCTTTGCATTTAGCGGGAGCGTCTTCTATTATAGGGGCTGTTAATTTTATTACGACTATTTTGAATATGCGCATGCTTGGAATAAGCATGGATAAAACACCTTTATTTGTTTGGTCTGTGTTGATTACAGCGGTTTTGTTGTTATTGTCTCTTCCTGTGCTCGCGGGGGCGATTACGATATTGTTAACGGATCGGAATTTTAATACTTCGTTTTTTGACCCCGCGGGGGGAGGGGATCCTGTTTTATTTCAACATCTN

>MG573899 Leptonetela_penevi

NNNNNNNNNNNNNNNNNNNNNNNNNNNNNNNNNNNNNNNNNNNNNNNNNNNNNNNNNNNNNNNNNNNNNNNNNNNNNNNNNNNNNNNNNNNNNNNNNNNNNNNNNNNNNNNNNNNNNNNNNNNNNNNNNNNNNNNNNNNNNNNNNNNNNNNNNNNNNNNNNNNNNNNNNNNNNNNNNNNNNNNNNNNNNNNNNNNNNNNNNNNNNNNNNNNNNNNNNNNNNNNNNNNNNNNNNNNNNNCGGATAAATAATTTGAGTTTTTGATTGTTGCCGCCGTCTTTGTTTTTGCTGGTAGTTTCTGGGATAGTGGAGGTAGGGGTTGGTACGGGGTGAACTATTTATCCTCCTTTGGCTGCATATGTGGGACATTCGTTTGTGTCGGTGGATTATGCTATTTTTTCTTTACATTTAGCGGGAGCGTCTTCTATTATAGGGGCTGTTAATTTTATTACGACTATTTTGAATATACGAATGTCTGGAATAAGTATGGATAAGACGCCTTTGTTTGTTTGATCAGTGTTGATTACGGCGGTACTGTTGTTGTTATCTCTTCCTGTATTAGCGGGGGCAATTACGATGTTGTTAACGGATCGGAATTTCAATACTTCATTTTTTGATCCTGCAGGGGGAGGGGATCCTGTTTTGTTTCAACATTTN

>MG573895 Leptonetela_chiosensis

NNNNNNNNNNNNNNNNNNNNNNNNNNNNNNNNNNNNNNNNNNNNNNNNNNNNNNNNNNNNNNNNNNNNNNNNNNNNNNNNNNNNNNNNNNNNNNNNNNNNNNNNNNNNNNNNNNNNNNNNNNNNNNNNNNNNNNNNNNNNNNNNNNNNNNNNNNNNNNNNNNNNNNNNNNNNNNNNNNNNNNNNNNNNNNNNNNNNNNNNNNNNNNNNNNNNNNNNNNNNNNNNNNNNNNNNNNNNNNCGGATAAATAATTTAAGTTTTTGGTTGTTGCCGCCATCTTTGTTTTTGTTGGTGGCTTCTGGGTTAGTGGAAATAGGGGTGGGGACTGGGTGGACTGTTTATCCGCCATTGGCTGCGTATTCTGGTCATTCTTTTGTGTCAGTAGATTATGCTATTTTTTCTTTACATTTAGCGGGGGCGTCTTCTATTATGGGGGCTGTTAATTTTATTACGACTATTTTGAATATGCGAACTAGTGGCATGAGAGTGGATAGGATGCCTTTATTTGTTTGGTCTGTGCTTATTACTGCGGTGTTATTATTATTATCCCTTCCAGTGTTAGCTGGGGCTATTACGATGTTATTGACGGATCGTAATTTTAATACTTCATTTTTTGATCCTGCTGGAGGGGGGGATCCTGTGTTGTTTCAGCATTTN

>MG573909 Leptonetela_thracia

NNNNNNNNNNNNNNNNNNNNNNNNNNNNNNNNNNNNNNNNNNNNNNNNNNNNNNNNNNNNNNNNNNNNNNNNNNNNNNNNNNNNNNNNNNNNNNNNNNNNNNNNNNNNNNNNNNNNNNNNNNNNNNNNNNNNNNNNNNNNNNNNNNNNNNNNNNNNNNNNNNNNNNNNNNNNNNNNNNNNNNNNNNNNNNNNNNNNNNNNNNNNNNNNNNNNNNNNNNNNNNNNNNNNNNNNNNNNNNCGTATAAATAATTTGAGTTTTTGATTGTTGCCTCCGTCTTTGTTTATATTGGTTGTTTCTGGTGTGGTGGGGGCAGGGGTGGGTACTGGATGGACGGTTTATCCTCCGTTGGCTGCGTATTCTGGGCATTCATTTGTGTCGGTGGATTATGCTATTTTTTCTTTACATTTGGCTGGAGCATCTTCTATTATGGGGGCTATCAATTTTATTACAACTGTTTTGAATATGCGAACGAGGGGGATAAGAATGGATAGGATGCCTTTATTTGTTTGGTCTGTTTTAATTACAGCGGTTTTGTTGTTGTTATCACTTCCAGTATTGGCGGGTGCTATTACTATATTGTTGACGGATCGAAATTTTAACACTTCATTCTTTGATCCTGCAGGAGGGGGGGATCCAGTGTTATTTCAACATTTN

>KU318413 Protoleptoneta_bulgarica

NNNNNNNNNNNNNNNNNNNNNNNNNNNNNNNNNNNNNNNNNNNNNNNNNNNNNNNNNNNNNNNNNNNNNNNNNNNNNNNNNNNNNNNNNNNNNNNNNNNNNNNNNNNNNNNNNNNNNNNNNNNNNNNNNNNNNNNNNNNNNNNNNNNNNNNNNNNNNNNNNNNNNNNNNNNNNNNNNNNNNNNNNNNNNNNNNNNNNNNNNNNNNNNNNNNNNNNNNNNNNNNNNNNNNNNNNNNNNNCGAATGAATAATTTGAGTTTTTGATTGTTGCCACCCTCTTTATTTTTGTTGGTTGTGTCTGGCATAGTTGAAATAGGGGTAGGTACTGGTTGGACGGTGTATCCTCCGTTGTCTTCGTATTTGGGGCATTCTCATGTTTCTGTTGATTTTGCTATTTTTTCTTTGCATTTGGCTGGGGCTTCGTCAATTATGGGGGCTATTAATTTTATTAGAACCATTATTAACATGCGTTCTGTTGGTATGAGAATGGATAGGGTTCCATTGTTTGTGTGATCGGTATTAATTACTGCTGTTTTGTTGTTGTTGTCTCTTCCTGTATTGGCTGGGGCTATTACTATGTTGTTAACGGATCGGAACTTTAATACTTCTTTTTTTGATCCTGCTGGAGGCGGGGATCCAATTCTTTTTCAGCACTTN

>MT987955 Cataleptoneta_aesculapii

NNNNNNNNNNNNNNNNNNNNNNNNNNNNNNNNNNNNNNNNNNNNNNNNNNNNNNNNNNNNNNNNNNNNNNNNNNNNNNNNNNNNNNNNNNNNNNNNNNNNNNNNNNNNNNNNNNNNNNNNNNNNNNNNNNNNNNNNNNNNNNNNNNNNNNNNNNNNNNNNNNNNNNNNNNNNNNNNNNNNNNNNNNNNNNNNNNNNNNNNNNNNNNNNNNNNNNNNNNNNNNNNNNNNNNNNNNNNNNCGTATGAATAATTTAAGATTTTGGTTGTTGCCCCCTTCTTTATTTTTTTTGATTGTTTCTAGAATAGTAGAGATAGGAGTAGGGACGGGGTGGACGTTGTATCCTCCTCTGGCTAGTTATGTGGGGCATGGTTTTATTTCGGTGGATTATGCAATTTTTTCTCTTCATTTGGCTGGTGCTTCGTCTATTATGGGGGCTATTAATTTTATTACTACTATTATTAACATGCGAAGAAATGGGCTAGTAATGGAGAGGGTATCATTGTTTGTTTGGTCTGTTTTGATTACAGCGGTGTTGTTGTTGTTATCTTTACCTGTTTTGGCGGGAGCCATTACTATATTGTTGACTGATCGAAACTTTAATACTTCTTTTTTTGACCCTGCGGGGGGTGGTGATCCGATTTTGTTTCAACATTTN

>MG573904 Leptonetela_arvanitidisi

NNNNNNNNNNNNNNNNNNNNNNNNNNNNNNNNNNNNNNNNNNNNNNNNNNNNNNNNNNNNNNNNNNNNNNNNNNNNNNNNNNNNNNNNNNNNNNNNNNNNNNNNNNNNNNNNNNNNNNNNNNNNNNNNNNNNNNNNNNNNNNNNNNNNNNNNNNNNNNNNNNNNNNNNNNNNNNNNNNNNNNNNNNNNNNNNNNNNNNNNNNNNNNNNNNNNNNNNNNNNNNNNNNNNNNNNNNNNNNCGAATGAATAATTTAAGTTTTTGGTTATTACCTCCGTCGCTTTTTTTGTTGGTAGTCTCTAGACTAGTAGGGGTGGGGGCTGGCACGGGTTGAACTGTTTATCCCCCGTTAGCGGCATATTTAGGGCATTCGTTTGTGTCAGTAGATTATGCTATTTTCTCTTTACATTTAGCTGGGGCGTCTTCTATTATAGGGGCTGTCAATTTTATTACGACTATTTTAAATATGCGTACATGGGGTATGAGAATAGATAAGACTCCCTTGTTTGTTTGATCTGTGTTGATTACAGCGGTTTTACTGTTGTTATCCCTTCCCGTGTTAGCGGGAGCGATCACAATGTTATTGACTGATCGTAATTTTAATACTTCATTTTTTGATCCTGCGGGGGGGGGGGATCCTGTGTTATTTCAGCATTTN

>Cataleptoneta_sulfur_S2 Cataleptoneta_sulfur_S2

NNNNNNNNNNNNNNNNNNNNNNNNNNNNNNNNNNNNNNNNNNNNNNNNNNNNNNNNNNNNNNNNNNNNNNNNNNNNNNNNNNNNNNNNNNNNNNNNNNNNNNNNNNNNNNNNNNNNNNNNNNNNNNNNNNNNNNNNNNNNNNNNNNNNNNNNNNNNNNNNNNNNNNNNNNNNNNNNNNNNNNNNNNNNNNNNNNNNNNNNNNNNNNNNNNNNNNNNNNNNNNNNNNNNNNNNNNNNNNCGTATGAATAATTTGAGTTTTTGATTGTTGCCGCCATCGTTGACTTTGTTAGTAATTTCAAGAATGGTGGAAATTGGGGCGGGGACTGGATGAACTGTGTATCCTCCTTTAGCTTCGTATATAGGGCATGCATTTTGTTCCGTAGATTTTGTTATTTTTTCCTTACATTTTGCTGGGGCGTCTTCGATTATAGGGGCTATTAATTTTATTACTACTATTATTAATATACGAAGAGGAGGGATAACAATAGAAAAGGTACCTTTGTTTGTTTGATCTGTTTTGGTTACTGCCGTATTGCTTTTATTGTCTTTACCTGTATTGGCTGGTGCTATTACTATATTGTTGACGGATCGAAACTTTAATACATCTTTTTTCGATCCTGCCGGGGGGGGGGATCCGGTTCTATTTCAGCATTTN

>Cataleptoneta_sulfur_T2 Cataleptoneta_sulfur_T2

NNNNNNNNNNNNNNNNNNNNNNNNNNNNNNNNNNNNNNNNNNNNNNNNNNNNNNNNNNNNNNNNNNNNNNNNNNNNNNNNNNNNNNNNNNNNNNNNNNNNNNNNNNNNNNNNNNNNNNNNNNNNNNNNNNNNNNNNNNNNNNNNNNNNNNNNNNNNNNNNNNNNNNNNNNNNNNNNNNNNNNNNNNNNNNNNNNNNNNNNNNNNNNNNNNNNNNNNNNNNNNNNNNNNNNNNNNNNNNCGTATGAATAATTTGAGTTTTTGATTGTTGCCGCCATCGTTGATTTTGTTAGTAATTTCAAGAATGGTGGAAATTGGGGCGGGGACTGGATGAACTGTGTATCCTCCTTTAGCTTCGTATATAGGGCATGCATTTTGTTCCGTAGATTTTGTTATTTTTTCCTTACATTTTGCTGGGGCGTCTTCGATTATAGGGGCTATTAATTTTATTACTACTATTATTAATATACGAAGTGGAGGGATAACAATAGAAAAGGTACCTTTGTTTGTTTGATCTGTTTTGGTTACTGCCGTATTGCTTTTATTGTCTTTACCTGTATTGGCTGGTGCTATTACTATATTGTTGACGGATCGAAACTTTAATACATCTTTTTTCGACCCTGCCGGGGGGGGGGATCCGGTTCTATTTCAGCATTTN

>MT987968 Barusia sp. 3 HZ-2020

NNNNNNNNNNNNNNNNNNNNNNNNNNNNNNNNNNNNNNNNNNNNNNNNNNNNNNNNNNNNNNNNNNNNNNNNNNNNNNNNNNNNNNNNNNNNNNNNNNNNNNNNNNNNNNNNNNNNNNNNNNNNNNNNNNNNNNNNNNNNNNNNNNNNNNNNNNNNNNNNNNNNNNNNNNNNNNNNNNNNNNNNNNNNNNNNNNNNNNNNNNNNNNNNNNNNNNNNNNNNNNNNNNNNNNNNNNNNNNCGGATAAATAATTTGAGTTTTTGATTGTTACCCCCTTCGTTAAGACTTTTGCTTATTTCTGGGATGGTGGAAATAGGGGCAGGAACGGGATGAACTATTTATCCTCCTTTGGCTTCTTATGTGGGGCATCCTTTTGTGTCTGTGGATTTTGCTATCTTTTCTTTACATTTAGCAGGGGCGTCTTCTATTATAGGAGCTATTAATTTTATCACGACTATTTTAAATATACGGAGTGTAGGAATGACGATGGATAAGGTGCCTTTATTTGTTTGGTCGGTTTTAGTGACAGCGGTGTTGTTGTTGTTATCCTTGCCGGTATTAGCGGGGGCGGTCACAATATTATTGACGGATCGAAATTTTAATACGTCTTTTTTTGACCCTGCTGGAGGAGGAGATCCGGTGTTGTTTCAACATTTA

>MT987970 Barusia sp. 6 HZ-2020

NNNNNNNNNNNNNNNNNNNNNNNNNNNNNNNNNNNNNNNNNNNNNNNNNNNNNNNNNNNNNNNNNNNNNNNNNNNNNNNNNNNNNNNNNNNNNNNNNNNNNNNNNNNNNNNNNNNNNNNNNNNNNNNNNNNNNNNNNNNNNNNNNNNNNNNNNNNNNNNNNNNNNNNNNNNNNNNNNNNNNNNNNNNNNNNNNNNNNNNNNNNNNNNNNNNNNNNNNNNNNNNNNNNNNNNNNNNNNNCGGATGAATAATTTGAGTTTTTGATTGTTGCCACCGTCATTAAGATTATTAATTATTTCAGGAATAGTGGAGATAGGAGCGGGAACTGGGTGAACGATTTATCCGCCCTTGGCTTCTTATATTGGTCATCCGTTTGTTTCTGTGGATTTTGCTATTTTTTCTTTACATTTAGCGGGGGCGTCTTCTATTATAGGGGCTATTAATTTTATTACAACCGTGTTAAATATACGTAGAGTTGGGATGACTATAGATAAAGTGCCTTTATTTGTTTGGTCTGTTTTGGTCACTGCGGTGTTGTTGTTGTTGTCTTTACCTGTTTTGGCAGGTGCGATTACAATATTGTTGACGGATCGGAATTTTAATACATCATTTTTTGATCCTGCCGGGGGAGGGGATCCAGTGTTATTTCAACATTTA

>MT987971 Barusia sp. 7 HZ-2020

NNNNNNNNNNNNNNNNNNNNNNNNNNNNNNNNNNNNNNNNNNNNNNNNNNNNNNNNNNNNNNNNNNNNNNNNNNNNNNNNNNNNNNNNNNNNNNNNNNNNNNNNNNNNNNNNNNNNNNNNNNNNNNNNNNNNNNNNNNNNNNNNNNNNNNNNNNNNNNNNNNNNNNNNNNNNNNNNNNNNNNNNNNNNNNNNNNNNNNNNNNNNNNNNNNNNNNNNNNNNNNNNNNNNNNNNNNNNNNCGGATGAATAATTTGAGTTTTTGATTGTTGCCACCGTCATTAAGATTATTGATTATTTCGGGAATGGTGGAGATAGGAGCGGGAACTGGGTGAACGATTTATCCGCCCTTGGCTTCTTATATTGGTCATCCGTTTGTTTCTGTGGATTTTGCTATTTTTTCTTTACATTTAGCGGGGGCGTCTTCTATTATAGGGGCTATTAATTTTATTACAACCGTGTTAAATATACGTAGAGTTGGGATGACTATAGATAAAGTGCCTTTATTTGTTTGGTCTGTTTTGGTCACTGCGGTGTTGTTGTTATTATCTTTACCTGTTTTGGCAGGTGCGATTACAATATTGTTGACGGATCGGAATTTTAATACATCATTTTTTGATCCTGCAGGGGGAGGGGACCCAGTGTTATTTCAACATTTA

>JN378135 Calileptoneta_noyoana

NNNNNNNNNNNNNNNNNNNNNNNNNNNNNNNNNNNNNNNNNNNNNNNNNNNNNNNNNNNNNNNNNNNNNNNNNNNNNNNNNNNNNNNNNNNNNNNNNNNNNNNNNNNNNNNNNNNNNNNNNNNNNNNNNNNNNNNNNNNNNNNNNNNNNNNNNNNNNNNNNNNNNNNNNNNNNNNNNNNNNNNNNNNNNNNNNNNNNNNNNNNNNNNNNNNNNNNNNNNNNNNNNNNNNNNNNNNNNTCGTATAAATAATTTGAGTTTTTGATTACTTCCGCCTTCTTTATTATTGTTGTTATTTTCTAGAATAGTGGAGTTAGGGGCGGGTACTGGATGAACTGTTTATCCTCCGCTAGCATCTTATTTTGGGCATGCATTTGTTTCTGTTGATTTTGCTATTTTTTCTTTACACTTGGCAGGGGCTTCTTCTATTATAGGGGCGGTTAATTTTATTACTACTATTATTAATATACGTGTAAGAGGTATATCTTTTGATAAGGTATCTCTTTTTGTATGATCAGTTTTGATTACTGCGGTTTTGTTATTATTATCTCTTCCGGTATTAGCTGGTGCTATTACTATGTTGTTAACTGATCGTAATTTTAATACTTCTTTTTTTGATCCTTCAGGAGGAGGAGATCCTATTTTGTTTCAGCATTTG

>JN378140 Calileptoneta_helferi_voucher_904

NNNNNNNNNNNNNNNNNNNNNNNNNNNNNNNNNNNNNNNNNNNNNNNNNNNNNNNNNNNNNNNNNNNNNNNNNNNNNNNNNNNNNNNNNNNNNNNNNNNNNNNNNNNNNNNNNNNNNNNNNNNNNNNNNNNNNNNNNNNNNNNNNNNNNNNNNNNNNNNNNNNNNNNNNNNNNNNNNNNNNNNNNNNNNNNNNNNNNNNNNNNNNNNNNNNNNNNNNNNNNNNNNNNNNNNNNNNNNTCGTATGAATAATTTAAGATTTTGGTTGTTGCCGCCTTCTTTATTATTGTTGTTGTTTTCTAGCATGGTCGAGTTAGGGGCAGGTACCGGGTGAACTGTTTATCCCCCGCTTGCTTCTTATTTTGGGCATTCTTTTATTTCTGTGGATTTTGCTATTTTTTCTTTGCACTTAGCTGGGGCTTCTTCTATTATGGGGGCGGTAAACTTTATTTCTACTATTTTTAACATGCGAATGAAGGGCATGACTATAGATAGGGTCCCTCTTTTTGTGTGATCTGTTTTAATTACCGCGGTTTTGTTATTATTATCCCTTCCAGTGTTGGCTGGGGCCATTACTATGTTGTTGACTGATCGTAATTTTAATACGTCTTTTTTCGATCCTGCCGGTGGGGGGGACCCAGTTTTATTTCAGCATTTG

>JN378147 Calileptoneta_sp__undet__JL_2011_9040372

NNNNNNNNNNNNNNNNNNNNNNNNNNNNNNNNNNNNNNNNNNNNNNNNNNNNNNNNNNNNNNNNNNNNNNNNNNNNNNNNNNNNNNNNNNNNNNNNNNNNNNNNNNNNNNNNNNNNNNNNNNNNNNNNNNNNNNNNNNNNNNNNNNNNNNNNNNNNNNNNNNNNNNNNNNNNNNNNNNNNNNNNNNNNNNNNNNNNNNNNNNNNNNNNNNNNNNNNNNNNNNNNNNNNNNNNNNNNNNNNNNNNNNTAATTTGAGTTTTTGGTTGTTGCCACCCTCTTTGTTGTTGTTGTTATTTTCTAGTATGGTGGAGTTGGGGGCGGGTACTGGATGAACCATTTATCCTCCGCTTTCTTCGTATTTTGGTCATTCTTTTATTTCTGTGGATTTTGCTATTTTTTCTTTGCATTTAGCCGGAGCTTCTTCTATTATGGGAGCGGTGAATTTTATTACTACTATTGTAAATATACGTGTGGCTGGTATATCTATGGATAGGGTACCTCTTTTTGTTTGATCTGTTTTAATCACTGCAGTTTTGTTGTTATTATCGCTCCCGGTTTTGGCGGGAGCTATCACTATGTTATTAACTGATCGAAATTTTAATACTTCTTTTTTTGACCCCTCTGGGGGAGGGGATCCTATTTTGTTTCAGCATTTG

>JN378137 Calileptoneta_sp__undet__JL_2011_9040234

NNNNNNNNNNNNNNNNNNNNNNNNNNNNNNNNNNNNNNNNNNNNNNNNNNNNNNNNNNNNNNNNNNNNNNNNNNNNNNNNNNNNNNNNNNNNNNNNNNNNNNNNNNNNNNNNNNNNNNNNNNNNNNNNNNNNNNNNNNNNNNNNNNNNNNNNNNNNNNNNNNNNNNNNNNNNNNNNNNNNNNNNNNNNNNNNNNNNNNNNNNNNNNNNNNNNNNNNNNNNNNNNNNNNNNNNNNNNNTCGNATAAATAATTTAAGTTTTTGGTTGTTACCTCCCTCTCTATTGTTGTTGTTGTTTTCTAGAATGGTGGAATTAGGGGTGGGGACGGGGTGGACGGTATACCCCCCTCTTGCTTCTTATTTCGGACACTCTTTTATTTCTGTTGATTTTGCTATTTTTTCTTTACATTTGGCTGGAGCTTCTTCTATTATGGGGGCGGTTAATTTTATTTCTACTATTTTTAACATGCGAAGTGTGGGTATATCTATAGATAAAATTTCTCTCTTTGTATGATCTATACTAATTACGGCAATTTTATTGTTGTTGTCTCTTCCCGTATTAGCAGGTGCTATCACGATATTATTAACAGATCGAAATTTTAATACTTCTTTTTTTGACCCTGCAGGGGGTGGGGATCCTGTTTTATTTCAACATTTG

>BIOUG00198-D12 Cataleptoneta 1707809

NNNNNNNNNNNNNNNNNNNNNNNNNNNNNNNNNNNNNNNTGGTACTGCTTTAAGAATGTTGATTCGTGTTGAATTGATGCAAGGTGGATCATTTCTGGGAAACGATCATTTGTATAATGTGGTGGTGACTGCTCATGCATTTGTGATGATTTTTTTTATGGTTATACCAATTTTAATTGGTGGATTTGGAAATTGATTGGTTCCAATGATGATTGGGGCTCCAGATATGGCTTTTCCGCGTATGAATAATTTGAGATTTTGATTGTTGCCTCCTTCNNNNNNNNNNNNNNNNNNNNNNNNNNNNNNNNNNNNNNNNNNNNNNNNNNNNNNNNNNNNNNNNNNNNNNNNNNNNNNNNNNNNNNNNNNNNNNNNNNNNNNNNNNNNNNNNNNNNNNNNNNNNNNNNNNNNNNNNNNNNNNNNNNNNNNNNNNNNNNNNNNNNNNNNNNNNNNNNNNNNNNNNNNNNNNNNNNNNNNNNNNNNNNNNNNNNNNNNNNNNNNNNNNNNNNNNNNNNNNNNNNNNNNNNNNNNNNNNNNNNNNNNNNNNNNNNNNNNNNNNNNNNNNNNNNNNNNNNNNNNNNNNNNNNNNNNNNNNNNNNNNNNNNNNNNNNNNNNNNNNNNNNNNNNNNNNNNNNNNNNNNNNNNNNNNNNNNNNNNN

>BIOUG00198-E02 Cataleptoneta 1707811

NNNNNNNNNNNNNNNNNNNNNNNNNNNNNCTTCTATGGTTGGTACTGCTTTAAGAATGTTGATTCGTGTTGAATTGATGCAAGGTGGATCATTTCTGGGAAACGATCATTTGTATAATGTGGTGGTGACTGCTCATGCATTTGTGATGATTTTTTTTATGGTTATACCAATTTTAATTGGTGGATTTGGAAATTGATTGGTTCCAATGATGATTGGGGCTCCAGATATGGCTTTTCCGCGTATGAATAATTTGAGATTTTGATTGTTGCCTCCTTCNNNNNNNNNNNNNNNNNNNNNNNNNNNNNNNNNNNNNNNNNNNNNNNNNNNNNNNNNNNNNNNNNNNNNNNNNNNNNNNNNNNNNNNNNNNNNNNNNNNNNNNNNNNNNNNNNNNNNNNNNNNNNNNNNNNNNNNNNNNNNNNNNNNNNNNNNNNNNNNNNNNNNNNNNNNNNNNNNNNNNNNNNNNNNNNNNNNNNNNNNNNNNNNNNNNNNNNNNNNNNNNNNNNNNNNNNNNNNNNNNNNNNNNNNNNNNNNNNNNNNNNNNNNNNNNNNNNNNNNNNNNNNNNNNNNNNNNNNNNNNNNNNNNNNNNNNNNNNNNNNNNNNNNNNNNNNNNNNNNNNNNNNNNNNNNNNNNNNNNNNNNNNNNNNNNNNNNNNNN

>BIOUG00198-E01 Cataleptoneta 1707810

NNNNNNNNNNNNNNNNNNNNNNNNNNNNNNNNNNNNNNNNNNNACTGCTTTAAGAATGTTGATTCGTGTTGAATTGATGCAAGGTGGATCATTTCTGGGAAACGATCATTTGTATAATGTGGTGGTGACTGCTCATGCATTTGTGATGATTTTTTTTATGGTTATACCAATTTTAATTGGTGGATTTGGAAATTGATTGGTTCCAATGATGATTGGGGCTCCAGATATGGCTTTTCCGCGTATGAATAATTTGAGATTTTGATTGTTGCCTCCTTCATTTTTTTNNNNNNNNNNNNNNNNNNNNNNNNNNNNNNNNNNNNNNNNNNNNNNNNNNNNNNNNNNNNNNNNNNNNNNNNNNNNNNNNNNNNNNNNNNNNNNNNNNNNNNNNNNNNNNNNNNNNNNNNNNNNNNNNNNNNNNNNNNNNNNNNNNNNNNNNNNNNNNNNNNNNNNNNNNNNNNNNNNNNNNNNNNNNNNNNNNNNNNNNNNNNNNNNNNNNNNNNNNNNNNNNNNNNNNNNNNNNNNNNNNNNNNNNNNNNNNNNNNNNNNNNNNNNNNNNNNNNNNNNNNNNNNNNNNNNNNNNNNNNNNNNNNNNNNNNNNNNNNNNNNNNNNNNNNNNNNNNNNNNNNNNNNNNNNNNNNNNNNNNNNNNNNNNNNN

# Final alignment

>KU318413 Protoleptoneta_bulgarica

CGAATGAATAATTTGAGTTTTTGATTGTTGCCACCCTCTTTATTTTTGTTGGTTGTGTCTGGCATAGTTGAAATAGGGGTAGGTACTGGTTGGACGGTGTATCCTCCGTTGTCTTCGTATTTGGGGCATTCTCATGTTTCTGTTGATTTTGCTATTTTTTCTTTGCATTTGGCTGGGGCTTCGTCAATTATGGGGGCTATTAATTTTATTAGAACCATTATTAACATGCGTTCTGTTGGTATGAGAATGGATAGGGTTCCATTGTTTGTGTGATCGGTATTAATTACTGCTGTTTTGTTGTTGTTGTCTCTTCCTGTATTGGCTGGGGCTATTACTATGTTGTTAACGGATCGGAACTTTAATACTTCTTTTTTTGATCCTGCTGGAGGCGGGGATCCAATTCTTTTTCAGCACTTN

>BGE_00510_B06 Barusia korculana

CGAATAAATAATTTAAGTTTTTGATTACTACCTCCTTCTTTAGTTTTATTAATTGTTTCTAGAATAGTGGAGATTGGTGTAGGCACTGGATGGACCGTGTACCCGCCTTTGGCTTCGTATGTTGGACATGCTTACATTTCTGTGGATTTTGCTATTTTTTCTTTACATTTGGCTGGTGCTTCATCCATTATGGGGGCTATTAATTTTATTACTACAGTTATTAATATACGTAGAGGGGGGATAACTATGGAGAAAGTCCCATTGTTTGTTTGAAGAGTTTTGATTACAGCGGTTTTGCTATTATTATCTTTGCCTGTATTGGCGGGGGCTATCACCATATTACTTACTGATCGTAATTTTAATACATCATTTTTTGATCCAGCAGGAGGAGGGGATCCAGTCTTGTTTCAACATTTA

>BGE_00512_E05 Barusia insulana

CGAATAAATAATTTAAGATTCTGGTTGTTGCCCCCTTCTTTAGTTTTGTTAGTTGTTTCTAGAATAGTGGAGATTGGCGTGGGGACTGGATGGACTGTTTATCCTCCCCTAGCTTCGTATGTAGGACATGCTGTTGTTTCTGTGGATTTTGCTATTTTTTCTTTGCATTTGGCTGGTGCCTCATCTATTATGGGGGCTATTAATTTTATTACTACAGTTATCAATATACGAAGAGGTGGTATAACTATAGAGAAAGTTCCGTTATTTGTTTGGAGAGTTTTAATTACAGCGGTTTTATTGTTATTATCTTTACCTGTATTGGCCGGGGCTATTACTATGTTACTTACTGATCGGAATTTTAATACATCATTTTTTGACCCTGCTGGAGGGGGGGATCCAGTATTGTTTCAGCATTTA

>BGE_00513_H08 Barusia cf. maheni

CGAATGAATAATTTAAGATTTTGGTTGCTTCCACCTTCTTTAGTTTTGTTAATTATTTCTAGAATAGTGGAGATTGGAGCAGGAACTGGGTGAACTGTGTATCCGCCTTTAGCTTCATATGTTGGGCATGCTCATGTTTCTGTAGACTTTGCTATTTTTTCTTTGCATTTGGCTGGTGCTTCATCTATTATGGGGGCTATTAATTTTATTACTACGGTAATAAACATACGAAGAGGAGGAATGACCATAGATAAGGTTCCTTTATTTGTTTGAAGAGTGTTAATTACAGCGGTTTTATTATTATTGTCTTTGCCTGTGTTGGCTGGGGCTATTACTATGTTGCTTACTGATCGAAATTTTAATACATCATTTTTTGACCCTGCTGGTGGGGGGGATCCAGTATTGTTTCAGCATTTA

>BGE_00521_E05 Sulcia 26743027

CGAATGAATAACTTAAGTTTTTGGTTATTACCTCCTTCATTATCTTTGCTTCTTATTTCTGGAATGGTGGAAATGGGAGTGGGAACTGGGTGGACTATTTACCCTCCGTTGGCTTCTTATGTGGGGCATGCCTTTGTTTCTGTAGATTTTGCTATTTTTTCTTTACATTTGGCTGGAGCTTCTTCTATTATGGGGGCAATTAATTTTATTACTACTGTGGTAAACATACGAGGTGCGGGAATAACTATGGATAAGGTTCCTTTATTTGTATGGTCTGTTTTGATTACTGCTGTGTTATTACTTTTGTCTTTGCCTGTGTTGGCTGGTGCGATTACAATGTTGTTAACAGATCGTAATTTTAATACTTCGTTTTTTGATCCTGCAGGAGGGGGGGATCCTGTTTTATTTCAACATTTG

>BGE_00513_D08 Sulcia 24761902

CGTATAAATAATTTAAGTTTTTGATTGTTACCCCCTTCATTATCTTTGCTTCTTATTTCTGGAATGGTAGAAATAGGGGTGGGAACTGGATGGACTATTTATCCTCCATTGGCTTCTTATGTAGGGCATGCCTTTGTTTCTGTGGATTTTGCTATTTTTTCTTTGCATTTGGCTGGAGCTTCTTCTATTATGGGGGCAATTAATTTTATTACTACAGTGGTAAATATGCGAGGTGCTGGGATAACTATGGATAAGGTCCCTTTATTTGTGTGGTCTGTTTTAATTACTGCTGTGTTGTTACTTTTGTCTTTGCCTGTGTTGGCTGGAGCAATTACAATATTGTTAACGGATCGTAATTTTAATACTTCGTTTTTTGATCCTGCAGGAGGAGGAGATCCTGTTTTATTTCAACATTTG

>BGE_00521_D05 Sulcia 26743015

CGTATGAATAATTTAAGTTTTTGGTTATTACCTCCTTCATTATCTTTGCTTCTTATTTCTGGAATGGTTGAGATAGGGGTAGGGACTGGGTGGACTATTTATCCTCCTTTAGCTTCTTATGTGGGGCATGCCTTTGTTTCTGTGGATTTTGCTATTTTTTCTTTACATTTGGCTGGAGCTTCTTCTATTATGGGGGCGATTAATTTTATTACTACTGTGGTAAATATGCGAGGTGCTGGGATGACTATAGATAAGGTTCCTTTATTTGTGTGATCTGTTTTAGTTACTGCTGTGTTATTGCTCTTGTCTTTACCTGTGTTGGCTGGGGCCATTACAATATTATTGACAGATCGTAATTTTAATACTTCATTTTTTGATCCCGCAGGAGGAGGGGATCCTGTGTTATTCCAGCATTTG

>BSCRO41624 Cataleptoneta_lingulata

CGAATAAATAATTTAAGTTTTTGATTATTACCACCTTCGTTAATTTTGTTGATTATTTCTAGTATGGTAGAAATTGGTGTAGGTACTGGTTGAACTGTTTACCCTCCTTTAGCTTCTTATGTTGGGCATNNNNNNNNNNNNGTAGATTATGCTATTTTTTCATTACATTTAGCTGGTGCTTCGTCTATTATAGGTGCAGTTAATTTTATTACTACTGTAGTAAATATACGTAGTGGTGGAATAACTATGGAAAAAACGCCTTTATTTGTATGGTCTGTTTTAATTACTGCGGTGTTGTTATTATTGTCTTTACCCGTGTTGGCAGGTGCTATTACTATGTTATTGACGGATCGAAATTTTAATACTTCGTTTTTTGATCCTGCTGGTGGAGGGGATCCTGTTTTATTTCAACATTTN

>BSUIO106025 Cataleptoneta_detriticola

CGTATGAATAACTTGAGATTTTGGTTATTGCCTCCTTCTTTGTTATTATTAATTGTTTCTGGAATGGTAGAATTGGGAGTGGGGACGGGTTGGACTGTCTATCCTCCTTTGGCTTCTNNNNNNNNNNNNNNNNNNNNNTCTGTGGATTTTGCTATTTTTTCTTTACATTTGGCAGGGGCATCATCTATTATGGGGGCTATTAATTTTATTACTACTATTNNNNNNNNNNNNNNNNNNNNNNNNNNNNNNGAAAAAGTACCTTTGTTTGTATGGTCTGTATTGGTTACAGCAGTTTTATTATTGTTGTCGTTGCCTGTTTTAGCAGGAGCAATTACAATGTTATTAACTGATCGTAATTTTAATACTTCTTTTTTTGATCCTGCCGGGGGTGGAGATCCAGTATTGTTTCAACATTTN

>JN378155 Leptoneta_microphthalma

CGTATGAATAATTTGAGTTTTTGGTTGTTACCTCCTTCTTTATTTTTGTTGGTGGTTTCTAGAATAGTTGAAATAGGGGTTGGAACAGGTTGAACTGTATATCCTCCATTATCTTCTAATGTTGGACATGCTTTTGTGTCGGTTGATTTTGCTATTTTTTCTCTTCATTTGGCTGGAGCTTCTTCTATTATGGGGGCTGTAAATTTTATTACTACTATTGTAAATATGCGAACTTTTGGTATAACTATAGAAAAAGTTCCTTTGTTTGTTTGATCAGTGTTAGTGACTGCTGTTCTGTTGTTGCTTTCTTTACCTGTGTTAGCAGGTGCCATTACTATGTTGTTAACTGATCGTAATTTTAATACATCTTTTTTTGATCCTGCTGGTGGGGGGGATCCAGTGTTATTTCAACACTTN

>JN378156 Leptoneta convexa voucher 9040712

CGTATGAATAATTTGAGTTTTTGGTTATTACCCCCTTCTTTATTTTTATTAGTGGTTTCTAGAATAGTTGAAATAGGAGTAGGAACAGGTTGGACTGTGTATCCTCCATTGTCTTCTAATGTAGGGCATGCTTTTGTGTCGGTTGATTTTGCTATTTTTTCTCTTCATTTAGCTGGGGCTTCTTCTATTATAGGGGCTGTTAATTTCATTACTACTATTATTAATATGCGAACTTTTGGTATGAGTATGGAAAAGGTTCCCTTGTTTGTTTGATCAGTGTTAGTGACTGCTGTTTTGTTGTTGTTATCTTTACCTGTATTAGCTGGTGCTATTACTATGTTGTTAACTGATCGTAATTTTAATACCTCTTTTTTTGACCCTGCTGGCGGGGGGGATCCAGTGTTATTTCAACATTTG

>MT607793 Leptoneta_paroculus

CGAATGAATAATTTAAGTTTTTGATTGTTGCCTCCTTCTTTATTTTTATTGGTGGTTTCTAGAATAGTTGAAATAGGGGTGGGTACAGGATGAACTGTGTATCCTCCCTTGTCTTCTAATGTAGGGCATGCTTTTGTTTCTGTAGATTTTGCTATTTTTTCTCTTCATTTGGCGGGGGCTTCTTCTATTATGGGTGCTGTTAATTTTATTACTACTATTGTAAATATGCGAACTTTTGGAATGACAATGGAAAAAGTTCCATTGTTTGTGTGATCAGTGTTAGTTACTGCTGTTTTATTGTTGTTATCTTTACCAGTATTAGCTGGAGCTATTACTATATTATTAACAGATCGTAATTTTAATACTTCTTTTTTTGACCCCGCTGGGGGCGGGGATCCGGTATTGTTTCAACATTTN

>MW997984 Leptoneta_paroculus

CGAATGAATAATTTAAGTTTTTGATTGTTGCCTCCTTCTTTATTTTTATTGGTGGTTTCTAGAATAGTTGAAATAGGGGTGGGTACAGGATGAACTGTGTATCCTCCCTTGTCTTCTAATGTAGGGCATGCTTTTGTTTCTGTAGATTTTGCTATTTTTTCTCTTCATTTGGCGGGGGCTTCTTCTATTATGGGTGCTGTTAATTTTATTACTACTATTGTAAATATGCGAACTTTTGGAATGACAATGGAAAAAGTTCCATTGTTTGTGTGATCAGTGTTAGTTACTGCTGTTTTATTGTTGTTATCTTTACCAGTATTAGCTGGAGCTATTACTATATTATTAACAGATCGTAATTTTAATACTTCTTTTTTTGACCCCGCTGGGGGCGGGGATCCGGTATTGTTTCAACATTTN

>KY017764 Leptoneta_infuscata

CGTATAAATAATTTAAGATTTTGGTTGTTGCCACCTTCTTTATTTTTGTTAATTGTTTCTAGTATAGTAGAAATAGGGGTGGGAACTGGTTGGACTGTTTATCCCCCTTTAGCTTCTAATGTTGGTCATGCTTTTGTATCTGTAGATTTTGCTATTTTTTCTCTTCATTTGGCTGGAGCTTCTTCTATTATGGGGGCTATTAATTTTATTACTACTATTATGAATATGCGTACTTTTGGGATGAGAATGGAGAAGGTTCCATTATTTGTTTGGTCGGTATTAGTAACTGCTATTTTGTTATTATTGTCTTTACCTGTGTTAGCAGGTGCTATTACTATATTGCTAACAGATCGAAACTTTAATACTTCTTTTTTTGATCCTGCTGGAGGGGGAGATCCAGTTTTATTTCAACATTTN

>MT98795 Leptonetela_caucasica

CGAATAAATAATTTGAGATTTTGGTTATTGCCACCCTCTTTATTTTTATTGGTAGTTTCTGGTATGGTAGAGATAGGGGTGGGCACTGGTTGAACTATTTATCCTCCTTTGGCTGCTTATTTGGGGCATTCTTTTGTGTCAGTGGATTATGCTATTTTTTCTTTACATTTAGCTGGTGCTTCTTCTATTATGGGGGCTGTTAATTTTATTACTACTATTTTTAATATGCGTATAAATGGGATGAGAATGGAGAAGATGCCATTATTTGTTTGGTCAGTATTAATTACAGCAGTTTTATTATTATTGTCACTTCCAGTATTGGCTGGGGCCATTACTATGTTGCTAACAGATCGAAATTTTAATACTTCTTTTTTTGATCCGGCAGGTGGGGGAGATCCTGTTTTATTTCAGCATTTN

>MG573884 Leptonetela_paragamiani

CGAATGAATAATTTGAGTTTTTGATTGTTACCTCCATCTTTGTTTTTATTGGTAGTTTCTGGGATGGTAGAGGTAGGGGTTGGGACGGGATGAACTATTTATCCTCCTTTGGCTGCATATGTGGGACATTCTTTTGTGTCGGTGGATTATGCTATTTTTTCTTTGCATTTAGCGGGAGCGTCTTCTATTATAGGGGCTGTTAATTTTATTACGACTATTTTGAATATGCGCATGCTTGGAATAAGCATGGATAAAACACCTTTATTTGTTTGGTCTGTGTTGATTACAGCGGTTTTGTTGTTATTGTCTCTTCCTGTGCTCGCGGGGGCGATTACGATATTGTTAACGGATCGGAATTTTAATACTTCGTTTTTTGACCCCGCGGGGGGAGGGGATCCTGTTTTATTTCAACATCTN

>MG573899 Leptonetela_penevi

CGGATAAATAATTTGAGTTTTTGATTGTTGCCGCCGTCTTTGTTTTTGCTGGTAGTTTCTGGGATAGTGGAGGTAGGGGTTGGTACGGGGTGAACTATTTATCCTCCTTTGGCTGCATATGTGGGACATTCGTTTGTGTCGGTGGATTATGCTATTTTTTCTTTACATTTAGCGGGAGCGTCTTCTATTATAGGGGCTGTTAATTTTATTACGACTATTTTGAATATACGAATGTCTGGAATAAGTATGGATAAGACGCCTTTGTTTGTTTGATCAGTGTTGATTACGGCGGTACTGTTGTTGTTATCTCTTCCTGTATTAGCGGGGGCAATTACGATGTTGTTAACGGATCGGAATTTCAATACTTCATTTTTTGATCCTGCAGGGGGAGGGGATCCTGTTTTGTTTCAACATTTN

>MG573895 Leptonetela_chiosensis

CGGATAAATAATTTAAGTTTTTGGTTGTTGCCGCCATCTTTGTTTTTGTTGGTGGCTTCTGGGTTAGTGGAAATAGGGGTGGGGACTGGGTGGACTGTTTATCCGCCATTGGCTGCGTATTCTGGTCATTCTTTTGTGTCAGTAGATTATGCTATTTTTTCTTTACATTTAGCGGGGGCGTCTTCTATTATGGGGGCTGTTAATTTTATTACGACTATTTTGAATATGCGAACTAGTGGCATGAGAGTGGATAGGATGCCTTTATTTGTTTGGTCTGTGCTTATTACTGCGGTGTTATTATTATTATCCCTTCCAGTGTTAGCTGGGGCTATTACGATGTTATTGACGGATCGTAATTTTAATACTTCATTTTTTGATCCTGCTGGAGGGGGGGATCCTGTGTTGTTTCAGCATTTN

>MG573909 Leptonetela_thracia

CGTATAAATAATTTGAGTTTTTGATTGTTGCCTCCGTCTTTGTTTATATTGGTTGTTTCTGGTGTGGTGGGGGCAGGGGTGGGTACTGGATGGACGGTTTATCCTCCGTTGGCTGCGTATTCTGGGCATTCATTTGTGTCGGTGGATTATGCTATTTTTTCTTTACATTTGGCTGGAGCATCTTCTATTATGGGGGCTATCAATTTTATTACAACTGTTTTGAATATGCGAACGAGGGGGATAAGAATGGATAGGATGCCTTTATTTGTTTGGTCTGTTTTAATTACAGCGGTTTTGTTGTTGTTATCACTTCCAGTATTGGCGGGTGCTATTACTATATTGTTGACGGATCGAAATTTTAACACTTCATTCTTTGATCCTGCAGGAGGGGGGGATCCAGTGTTATTTCAACATTTN

>MT987955 Cataleptoneta_aesculapii

CGTATGAATAATTTAAGATTTTGGTTGTTGCCCCCTTCTTTATTTTTTTTGATTGTTTCTAGAATAGTAGAGATAGGAGTAGGGACGGGGTGGACGTTGTATCCTCCTCTGGCTAGTTATGTGGGGCATGGTTTTATTTCGGTGGATTATGCAATTTTTTCTCTTCATTTGGCTGGTGCTTCGTCTATTATGGGGGCTATTAATTTTATTACTACTATTATTAACATGCGAAGAAATGGGCTAGTAATGGAGAGGGTATCATTGTTTGTTTGGTCTGTTTTGATTACAGCGGTGTTGTTGTTGTTATCTTTACCTGTTTTGGCGGGAGCCATTACTATATTGTTGACTGATCGAAACTTTAATACTTCTTTTTTTGACCCTGCGGGGGGTGGTGATCCGATTTTGTTTCAACATTTN

>MG573904 Leptonetela_arvanitidisi

CGAATGAATAATTTAAGTTTTTGGTTATTACCTCCGTCGCTTTTTTTGTTGGTAGTCTCTAGACTAGTAGGGGTGGGGGCTGGCACGGGTTGAACTGTTTATCCCCCGTTAGCGGCATATTTAGGGCATTCGTTTGTGTCAGTAGATTATGCTATTTTCTCTTTACATTTAGCTGGGGCGTCTTCTATTATAGGGGCTGTCAATTTTATTACGACTATTTTAAATATGCGTACATGGGGTATGAGAATAGATAAGACTCCCTTGTTTGTTTGATCTGTGTTGATTACAGCGGTTTTACTGTTGTTATCCCTTCCCGTGTTAGCGGGAGCGATCACAATGTTATTGACTGATCGTAATTTTAATACTTCATTTTTTGATCCTGCGGGGGGGGGGGATCCTGTGTTATTTCAGCATTTN

>Cataleptoneta_sulfur_S2

CGTATGAATAATTTGAGTTTTTGATTGTTGCCGCCATCGTTGACTTTGTTAGTAATTTCAAGAATGGTGGAAATTGGGGCGGGGACTGGATGAACTGTGTATCCTCCTTTAGCTTCGTATATAGGGCATGCATTTTGTTCCGTAGATTTTGTTATTTTTTCCTTACATTTTGCTGGGGCGTCTTCGATTATAGGGGCTATTAATTTTATTACTACTATTATTAATATACGAAGAGGAGGGATAACAATAGAAAAGGTACCTTTGTTTGTTTGATCTGTTTTGGTTACTGCCGTATTGCTTTTATTGTCTTTACCTGTATTGGCTGGTGCTATTACTATATTGTTGACGGATCGAAACTTTAATACATCTTTTTTCGATCCTGCCGGGGGGGGGGATCCGGTTCTATTTCAGCATTTN

>Cataleptoneta_sulfur_T2

CGTATGAATAATTTGAGTTTTTGATTGTTGCCGCCATCGTTGATTTTGTTAGTAATTTCAAGAATGGTGGAAATTGGGGCGGGGACTGGATGAACTGTGTATCCTCCTTTAGCTTCGTATATAGGGCATGCATTTTGTTCCGTAGATTTTGTTATTTTTTCCTTACATTTTGCTGGGGCGTCTTCGATTATAGGGGCTATTAATTTTATTACTACTATTATTAATATACGAAGTGGAGGGATAACAATAGAAAAGGTACCTTTGTTTGTTTGATCTGTTTTGGTTACTGCCGTATTGCTTTTATTGTCTTTACCTGTATTGGCTGGTGCTATTACTATATTGTTGACGGATCGAAACTTTAATACATCTTTTTTCGACCCTGCCGGGGGGGGGGATCCGGTTCTATTTCAGCATTTN

>MT987968 Barusia sp. 3 HZ-2020

CGGATAAATAATTTGAGTTTTTGATTGTTACCCCCTTCGTTAAGACTTTTGCTTATTTCTGGGATGGTGGAAATAGGGGCAGGAACGGGATGAACTATTTATCCTCCTTTGGCTTCTTATGTGGGGCATCCTTTTGTGTCTGTGGATTTTGCTATCTTTTCTTTACATTTAGCAGGGGCGTCTTCTATTATAGGAGCTATTAATTTTATCACGACTATTTTAAATATACGGAGTGTAGGAATGACGATGGATAAGGTGCCTTTATTTGTTTGGTCGGTTTTAGTGACAGCGGTGTTGTTGTTGTTATCCTTGCCGGTATTAGCGGGGGCGGTCACAATATTATTGACGGATCGAAATTTTAATACGTCTTTTTTTGACCCTGCTGGAGGAGGAGATCCGGTGTTGTTTCAACATTTA

>MT987970 Barusia sp. 6 HZ-2020

CGGATGAATAATTTGAGTTTTTGATTGTTGCCACCGTCATTAAGATTATTAATTATTTCAGGAATAGTGGAGATAGGAGCGGGAACTGGGTGAACGATTTATCCGCCCTTGGCTTCTTATATTGGTCATCCGTTTGTTTCTGTGGATTTTGCTATTTTTTCTTTACATTTAGCGGGGGCGTCTTCTATTATAGGGGCTATTAATTTTATTACAACCGTGTTAAATATACGTAGAGTTGGGATGACTATAGATAAAGTGCCTTTATTTGTTTGGTCTGTTTTGGTCACTGCGGTGTTGTTGTTGTTGTCTTTACCTGTTTTGGCAGGTGCGATTACAATATTGTTGACGGATCGGAATTTTAATACATCATTTTTTGATCCTGCCGGGGGAGGGGATCCAGTGTTATTTCAACATTTA

>MT987971 Barusia sp. 7 HZ-2020

CGGATGAATAATTTGAGTTTTTGATTGTTGCCACCGTCATTAAGATTATTGATTATTTCGGGAATGGTGGAGATAGGAGCGGGAACTGGGTGAACGATTTATCCGCCCTTGGCTTCTTATATTGGTCATCCGTTTGTTTCTGTGGATTTTGCTATTTTTTCTTTACATTTAGCGGGGGCGTCTTCTATTATAGGGGCTATTAATTTTATTACAACCGTGTTAAATATACGTAGAGTTGGGATGACTATAGATAAAGTGCCTTTATTTGTTTGGTCTGTTTTGGTCACTGCGGTGTTGTTGTTATTATCTTTACCTGTTTTGGCAGGTGCGATTACAATATTGTTGACGGATCGGAATTTTAATACATCATTTTTTGATCCTGCAGGGGGAGGGGACCCAGTGTTATTTCAACATTTA

>JN378135 Calileptoneta_noyoana

CGTATAAATAATTTGAGTTTTTGATTACTTCCGCCTTCTTTATTATTGTTGTTATTTTCTAGAATAGTGGAGTTAGGGGCGGGTACTGGATGAACTGTTTATCCTCCGCTAGCATCTTATTTTGGGCATGCATTTGTTTCTGTTGATTTTGCTATTTTTTCTTTACACTTGGCAGGGGCTTCTTCTATTATAGGGGCGGTTAATTTTATTACTACTATTATTAATATACGTGTAAGAGGTATATCTTTTGATAAGGTATCTCTTTTTGTATGATCAGTTTTGATTACTGCGGTTTTGTTATTATTATCTCTTCCGGTATTAGCTGGTGCTATTACTATGTTGTTAACTGATCGTAATTTTAATACTTCTTTTTTTGATCCTTCAGGAGGAGGAGATCCTATTTTGTTTCAGCATTTG

>JN378140 Calileptoneta_helferi_voucher_904

CGTATGAATAATTTAAGATTTTGGTTGTTGCCGCCTTCTTTATTATTGTTGTTGTTTTCTAGCATGGTCGAGTTAGGGGCAGGTACCGGGTGAACTGTTTATCCCCCGCTTGCTTCTTATTTTGGGCATTCTTTTATTTCTGTGGATTTTGCTATTTTTTCTTTGCACTTAGCTGGGGCTTCTTCTATTATGGGGGCGGTAAACTTTATTTCTACTATTTTTAACATGCGAATGAAGGGCATGACTATAGATAGGGTCCCTCTTTTTGTGTGATCTGTTTTAATTACCGCGGTTTTGTTATTATTATCCCTTCCAGTGTTGGCTGGGGCCATTACTATGTTGTTGACTGATCGTAATTTTAATACGTCTTTTTTCGATCCTGCCGGTGGGGGGGACCCAGTTTTATTTCAGCATTTG

>JN378147 Calileptoneta_sp__undet__JL_2011_9040372

NNNNNNNNTAATTTGAGTTTTTGGTTGTTGCCACCCTCTTTGTTGTTGTTGTTATTTTCTAGTATGGTGGAGTTGGGGGCGGGTACTGGATGAACCATTTATCCTCCGCTTTCTTCGTATTTTGGTCATTCTTTTATTTCTGTGGATTTTGCTATTTTTTCTTTGCATTTAGCCGGAGCTTCTTCTATTATGGGAGCGGTGAATTTTATTACTACTATTGTAAATATACGTGTGGCTGGTATATCTATGGATAGGGTACCTCTTTTTGTTTGATCTGTTTTAATCACTGCAGTTTTGTTGTTATTATCGCTCCCGGTTTTGGCGGGAGCTATCACTATGTTATTAACTGATCGAAATTTTAATACTTCTTTTTTTGACCCCTCTGGGGGAGGGGATCCTATTTTGTTTCAGCATTTG

>JN378137 Calileptoneta_sp__undet__JL_2011_9040234

CGNATAAATAATTTAAGTTTTTGGTTGTTACCTCCCTCTCTATTGTTGTTGTTGTTTTCTAGAATGGTGGAATTAGGGGTGGGGACGGGGTGGACGGTATACCCCCCTCTTGCTTCTTATTTCGGACACTCTTTTATTTCTGTTGATTTTGCTATTTTTTCTTTACATTTGGCTGGAGCTTCTTCTATTATGGGGGCGGTTAATTTTATTTCTACTATTTTTAACATGCGAAGTGTGGGTATATCTATAGATAAAATTTCTCTCTTTGTATGATCTATACTAATTACGGCAATTTTATTGTTGTTGTCTCTTCCCGTATTAGCAGGTGCTATCACGATATTATTAACAGATCGAAATTTTAATACTTCTTTTTTTGACCCTGCAGGGGGTGGGGATCCTGTTTTATTTCAACATTTG

# Best Model

Starting SMS v1.8.1

Input alignment	: align_rename.phy

Data type	: DNA

Number of taxa	: 51

Number of sites	: 655

Number of branches : 99

Criterion	: AIC

~~~~~~~~~~~~~~~~~~~~~~~~~~~~~~~~~~~~~~~~~~~~~~~~~~~~~~~~~~~~

Step 1 : Set a fixed topology	AIC=14640.78586

~~~~~~~~~~~~~~~~~~~~~~~~~~~~~~~~~~~~~~~~~~~~~~~~~~~~~~~~~~~~

Step 2 : Select the best decoration	AIC=14640.78586	decoration : '+G+I'

~~~~~~~~~~~~~~~~~~~~~~~~~~~~~~~~~~~~~~~~~~~~~~~~~~~~~~~~~~~~

Step 3 : Select the best matrix	AIC=0	matrix : 'GTR'

~~~~~~~~~~~~~~~~~~~~~~~~~~~~~~~~~~~~~~~~~~~~~~~~~~~~~~~~~~~~

Step 4 : Select the best final decoration	AIC=14640.78586	decoration : '+G+I'

~~~~~~~~~~~~~~~~~~~~~~~~~~~~~~~~~~~~~~~~~~~~~~~~~~~~~~~~~~~~

Selected model	: GTR +G+I

~~~~~~~~~~~~~~~~~~~~~~~~~~~~~~~~~~~~~~~~~~~~~~~~~~~~~~~~~~~~

Substitution model	: GTR

Equilibrium frequencies	: ML optimized

Proportion of invariable sites	: estimated (0.470)

Number of substitution rate categories	: 4

Gamma shape parameter	: estimated (0.719)

~~~~~~~~~~~~~~~~~~~~~~~~~~~~~~~~~~~~~~~~~~~~~~~~~~~~~~~~~~~~

# ML Tree

((((JN378137-Calileptoneta_sp__undet__JL_2011_9040234:0.4972677,JN378140-Calileptoneta_helferi_voucher_904:0.13008817):0.04364013[0.54],(JN378147-Calileptoneta_sp__undet__JL_2011_9040372:0.23832072,JN378135-Calileptoneta_noyoana:0.27007525):0.09451337[0.841]):0.37403929[0.988],(KU318413-Protoleptoneta_bulgarica:0.49478171,(MT98795-Leptonetela_caucasica:0.18321107,(MG573895-Leptonetela_chiosensis:0.25036223,(MG573909-Leptonetela_thracia:0.2717681,(MG573904-Leptonetela_arvanitidisi:0.2962897,(MG573899-Leptonetela_penevi:0.07894485,MG573884-Leptonetela_paragamiani:0.11364051):0.10901902[0.885]):0.12797456[0.899]):0.03336906[0.216]):0.14380411[0.847]):0.18673429[0.927]):0.04034733[0.466]):0.096806155[0.955],(((MT987955-Cataleptoneta_aesculapii:0.02244246,(BIOUG00198-E02-Cataleptoneta-1707811:0.00000008,(BIOUG00198-D12-Cataleptoneta-1707809:0.00000011,BIOUG00198-E01-Cataleptoneta-1707810:0.00000007):0.00000008[0]):0.26923905[0.82]):0.49966678[0.503],((Cataleptoneta_sulfur_T2-Cataleptoneta_sulfur_T2:0.0000006,Cataleptoneta_sulfur_S2-Cataleptoneta_sulfur_S2:0.0075271):0.44409684[1],((BSCRO41624-Cataleptoneta_lingulata:0.32142872,(BGE_00512_E05-Barusia-insulana:0.06521943,(BGE_00513_H08-Barusia-cf-maheni:0.16361134,BGE_00510_B06-Barusia-korculana:0.16640602):0.07318471[0.577]):0.3572151[0.98]):0.06495351[0.431],((MT987968-Barusia-sp-3-HZ-2020:0.22861922,(MT987970-Barusia-sp-6-HZ-2020:0.0000019,MT987971-Barusia-sp-7-HZ-2020:0.01829753):0.20186153[0.958]):0.20137372[0.926],((BGE_00513_B12-Sulcia-24761882:0.23875911,(BGE_00513_D09-Sulcia-24761903:0.07380973,BGE_00513_C03-Sulcia-24761885:0.01940076):0.22747821[0.993]):0.07900117[0.649],((BGE_00521_A11-Sulcia-26742985:0.09178031,(BGE_00521_F03-Sulcia-26743037:0.00416298,BGE_00521_E07-Sulcia-26743029:0.008429):0.08562463[0.963]):0.096836[0.947],((BGE_00513_G04-Sulcia-24761934:0.00000007,BGE_00513_G02-Sulcia-24761932:0.00512963):0.07291201[0.972],(BGE_00521_D05-Sulcia-26743015:0.08236943,(BGE_00513_D08-Sulcia-24761902:0.06362917,(BGE_00513_C08-Sulcia-24761890:0.05791684,(BGE_00521_C09-Sulcia-26743007:0.01598764,((BGE_00521_C05-Sulcia-26743003:0.00343756,BGE_00521_B06-Sulcia-26742992:0.00132134):0.06423568[1],(BGE_00521_D09-Sulcia-26743019:0.01071862,(BGE_00521_A01-Sulcia-26742975:0.00343593,(BGE_00521_E08-Sulcia-26743030:0.00000001,(BGE_00521_E05-Sulcia-26743027:0.00000001,(BGE_00521_B04-Sulcia-26742990:0.00000001,(BGE_00521_C11-Sulcia-26743009:0.00000001,BGE_00521_E09-Sulcia-26743031:0.00000001):0.00000001[0]):0.00000001[0]):0.00000001[0]):0.01244962[0.989]):0.00000009[0]):0.00611087[0.8]):0.00874921[0.624]):0.01661177[0.115]):0.04377084[0.968]):0.01352114[0.735]):0.03089301[0.739]):0.10389219[0.957]):0.08513637[0.88]):0.03078759[0.044]):0.1624009[0.947]):0.05191678[0.197]):0.0559003[0.75]):0.0485388[0.761],(BSUIO106025-Cataleptoneta_detriticola:0.41382198,(KY017764-Leptoneta_infuscata:0.16163216,((JN378156-Leptoneta-convexa-voucher-9040712:0.04995769,JN378155-Leptoneta_microphthalma:0.10754962):0.11185277[0.934],(MW997984-Leptoneta_paroculus:0.00000001,MT607793-Leptoneta_paroculus:0.00000001):0.11662932[0.922]):0.14403123[0.843]):0.14081848[0.86]):0.02400406[0]):0.096806155);

# Small Matrix, final alignment

>KU318413 Protoleptoneta_bulgarica

CGAATGAATAATTTGAGTTTTTGATTGTTGCCACCCTCTTTATTTTTGTTGGTTGTGTCTGGCATAGTTGAAATAGGGGTAGGTACTGGTTGGACGGTGTATCCTCCGTTGTCTTCGTATTTGGGGCATTCTCATGTTTCTGTTGATTTTGCTATTTTTTCTTTGCATTTGGCTGGGGCTTCGTCAATTATGGGGGCTATTAATTTTATTAGAACCATTATTAACATGCGTTCTGTTGGTATGAGAATGGATAGGGTTCCATTGTTTGTGTGATCGGTATTAATTACTGCTGTTTTGTTGTTGTTGTCTCTTCCTGTATTGGCTGGGGCTATTACTATGTTGTTAACGGATCGGAACTTTAATACTTCTTTTTTTGATCCTGCTGGAGGCGGGGATCCAATTCTTTTTCAGCACTTN

>BGE_00510_B06 Barusia korculana

CGAATAAATAATTTAAGTTTTTGATTACTACCTCCTTCTTTAGTTTTATTAATTGTTTCTAGAATAGTGGAGATTGGTGTAGGCACTGGATGGACCGTGTACCCGCCTTTGGCTTCGTATGTTGGACATGCTTACATTTCTGTGGATTTTGCTATTTTTTCTTTACATTTGGCTGGTGCTTCATCCATTATGGGGGCTATTAATTTTATTACTACAGTTATTAATATACGTAGAGGGGGGATAACTATGGAGAAAGTCCCATTGTTTGTTTGAAGAGTTTTGATTACAGCGGTTTTGCTATTATTATCTTTGCCTGTATTGGCGGGGGCTATCACCATATTACTTACTGATCGTAATTTTAATACATCATTTTTTGATCCAGCAGGAGGAGGGGATCCAGTCTTGTTTCAACATTTA

>BGE_00512_E05 Barusia insulana

CGAATAAATAATTTAAGATTCTGGTTGTTGCCCCCTTCTTTAGTTTTGTTAGTTGTTTCTAGAATAGTGGAGATTGGCGTGGGGACTGGATGGACTGTTTATCCTCCCCTAGCTTCGTATGTAGGACATGCTGTTGTTTCTGTGGATTTTGCTATTTTTTCTTTGCATTTGGCTGGTGCCTCATCTATTATGGGGGCTATTAATTTTATTACTACAGTTATCAATATACGAAGAGGTGGTATAACTATAGAGAAAGTTCCGTTATTTGTTTGGAGAGTTTTAATTACAGCGGTTTTATTGTTATTATCTTTACCTGTATTGGCCGGGGCTATTACTATGTTACTTACTGATCGGAATTTTAATACATCATTTTTTGACCCTGCTGGAGGGGGGGATCCAGTATTGTTTCAGCATTTA

>BGE_00513_H08 Barusia cf. maheni

CGAATGAATAATTTAAGATTTTGGTTGCTTCCACCTTCTTTAGTTTTGTTAATTATTTCTAGAATAGTGGAGATTGGAGCAGGAACTGGGTGAACTGTGTATCCGCCTTTAGCTTCATATGTTGGGCATGCTCATGTTTCTGTAGACTTTGCTATTTTTTCTTTGCATTTGGCTGGTGCTTCATCTATTATGGGGGCTATTAATTTTATTACTACGGTAATAAACATACGAAGAGGAGGAATGACCATAGATAAGGTTCCTTTATTTGTTTGAAGAGTGTTAATTACAGCGGTTTTATTATTATTGTCTTTGCCTGTGTTGGCTGGGGCTATTACTATGTTGCTTACTGATCGAAATTTTAATACATCATTTTTTGACCCTGCTGGTGGGGGGGATCCAGTATTGTTTCAGCATTTA

>BGE_00521_E05 Sulcia 26743027

CGAATGAATAACTTAAGTTTTTGGTTATTACCTCCTTCATTATCTTTGCTTCTTATTTCTGGAATGGTGGAAATGGGAGTGGGAACTGGGTGGACTATTTACCCTCCGTTGGCTTCTTATGTGGGGCATGCCTTTGTTTCTGTAGATTTTGCTATTTTTTCTTTACATTTGGCTGGAGCTTCTTCTATTATGGGGGCAATTAATTTTATTACTACTGTGGTAAACATACGAGGTGCGGGAATAACTATGGATAAGGTTCCTTTATTTGTATGGTCTGTTTTGATTACTGCTGTGTTATTACTTTTGTCTTTGCCTGTGTTGGCTGGTGCGATTACAATGTTGTTAACAGATCGTAATTTTAATACTTCGTTTTTTGATCCTGCAGGAGGGGGGGATCCTGTTTTATTTCAACATTTG

>BGE_00513_D08 Sulcia 24761902

CGTATAAATAATTTAAGTTTTTGATTGTTACCCCCTTCATTATCTTTGCTTCTTATTTCTGGAATGGTAGAAATAGGGGTGGGAACTGGATGGACTATTTATCCTCCATTGGCTTCTTATGTAGGGCATGCCTTTGTTTCTGTGGATTTTGCTATTTTTTCTTTGCATTTGGCTGGAGCTTCTTCTATTATGGGGGCAATTAATTTTATTACTACAGTGGTAAATATGCGAGGTGCTGGGATAACTATGGATAAGGTCCCTTTATTTGTGTGGTCTGTTTTAATTACTGCTGTGTTGTTACTTTTGTCTTTGCCTGTGTTGGCTGGAGCAATTACAATATTGTTAACGGATCGTAATTTTAATACTTCGTTTTTTGATCCTGCAGGAGGAGGAGATCCTGTTTTATTTCAACATTTG

>BGE_00521_D05 Sulcia 26743015

CGTATGAATAATTTAAGTTTTTGGTTATTACCTCCTTCATTATCTTTGCTTCTTATTTCTGGAATGGTTGAGATAGGGGTAGGGACTGGGTGGACTATTTATCCTCCTTTAGCTTCTTATGTGGGGCATGCCTTTGTTTCTGTGGATTTTGCTATTTTTTCTTTACATTTGGCTGGAGCTTCTTCTATTATGGGGGCGATTAATTTTATTACTACTGTGGTAAATATGCGAGGTGCTGGGATGACTATAGATAAGGTTCCTTTATTTGTGTGATCTGTTTTAGTTACTGCTGTGTTATTGCTCTTGTCTTTACCTGTGTTGGCTGGGGCCATTACAATATTATTGACAGATCGTAATTTTAATACTTCATTTTTTGATCCCGCAGGAGGAGGGGATCCTGTGTTATTCCAGCATTTG

>BSCRO41624 Cataleptoneta_lingulata

CGAATAAATAATTTAAGTTTTTGATTATTACCACCTTCGTTAATTTTGTTGATTATTTCTAGTATGGTAGAAATTGGTGTAGGTACTGGTTGAACTGTTTACCCTCCTTTAGCTTCTTATGTTGGGCATNNNNNNNNNNNNGTAGATTATGCTATTTTTTCATTACATTTAGCTGGTGCTTCGTCTATTATAGGTGCAGTTAATTTTATTACTACTGTAGTAAATATACGTAGTGGTGGAATAACTATGGAAAAAACGCCTTTATTTGTATGGTCTGTTTTAATTACTGCGGTGTTGTTATTATTGTCTTTACCCGTGTTGGCAGGTGCTATTACTATGTTATTGACGGATCGAAATTTTAATACTTCGTTTTTTGATCCTGCTGGTGGAGGGGATCCTGTTTTATTTCAACATTTN

>BSUIO106025 Cataleptoneta_detriticola

CGTATGAATAACTTGAGATTTTGGTTATTGCCTCCTTCTTTGTTATTATTAATTGTTTCTGGAATGGTAGAATTGGGAGTGGGGACGGGTTGGACTGTCTATCCTCCTTTGGCTTCTNNNNNNNNNNNNNNNNNNNNNTCTGTGGATTTTGCTATTTTTTCTTTACATTTGGCAGGGGCATCATCTATTATGGGGGCTATTAATTTTATTACTACTATTNNNNNNNNNNNNNNNNNNNNNNNNNNNNNNGAAAAAGTACCTTTGTTTGTATGGTCTGTATTGGTTACAGCAGTTTTATTATTGTTGTCGTTGCCTGTTTTAGCAGGAGCAATTACAATGTTATTAACTGATCGTAATTTTAATACTTCTTTTTTTGATCCTGCCGGGGGTGGAGATCCAGTATTGTTTCAACATTTN

>JN378155 Leptoneta_microphthalma

CGTATGAATAATTTGAGTTTTTGGTTGTTACCTCCTTCTTTATTTTTGTTGGTGGTTTCTAGAATAGTTGAAATAGGGGTTGGAACAGGTTGAACTGTATATCCTCCATTATCTTCTAATGTTGGACATGCTTTTGTGTCGGTTGATTTTGCTATTTTTTCTCTTCATTTGGCTGGAGCTTCTTCTATTATGGGGGCTGTAAATTTTATTACTACTATTGTAAATATGCGAACTTTTGGTATAACTATAGAAAAAGTTCCTTTGTTTGTTTGATCAGTGTTAGTGACTGCTGTTCTGTTGTTGCTTTCTTTACCTGTGTTAGCAGGTGCCATTACTATGTTGTTAACTGATCGTAATTTTAATACATCTTTTTTTGATCCTGCTGGTGGGGGGGATCCAGTGTTATTTCAACACTTN

>JN378156 Leptoneta convexa voucher 9040712

CGTATGAATAATTTGAGTTTTTGGTTATTACCCCCTTCTTTATTTTTATTAGTGGTTTCTAGAATAGTTGAAATAGGAGTAGGAACAGGTTGGACTGTGTATCCTCCATTGTCTTCTAATGTAGGGCATGCTTTTGTGTCGGTTGATTTTGCTATTTTTTCTCTTCATTTAGCTGGGGCTTCTTCTATTATAGGGGCTGTTAATTTCATTACTACTATTATTAATATGCGAACTTTTGGTATGAGTATGGAAAAGGTTCCCTTGTTTGTTTGATCAGTGTTAGTGACTGCTGTTTTGTTGTTGTTATCTTTACCTGTATTAGCTGGTGCTATTACTATGTTGTTAACTGATCGTAATTTTAATACCTCTTTTTTTGACCCTGCTGGCGGGGGGGATCCAGTGTTATTTCAACATTTG

>MT607793 Leptoneta_paroculus

CGAATGAATAATTTAAGTTTTTGATTGTTGCCTCCTTCTTTATTTTTATTGGTGGTTTCTAGAATAGTTGAAATAGGGGTGGGTACAGGATGAACTGTGTATCCTCCCTTGTCTTCTAATGTAGGGCATGCTTTTGTTTCTGTAGATTTTGCTATTTTTTCTCTTCATTTGGCGGGGGCTTCTTCTATTATGGGTGCTGTTAATTTTATTACTACTATTGTAAATATGCGAACTTTTGGAATGACAATGGAAAAAGTTCCATTGTTTGTGTGATCAGTGTTAGTTACTGCTGTTTTATTGTTGTTATCTTTACCAGTATTAGCTGGAGCTATTACTATATTATTAACAGATCGTAATTTTAATACTTCTTTTTTTGACCCCGCTGGGGGCGGGGATCCGGTATTGTTTCAACATTTN

>MW997984 Leptoneta_paroculus

CGAATGAATAATTTAAGTTTTTGATTGTTGCCTCCTTCTTTATTTTTATTGGTGGTTTCTAGAATAGTTGAAATAGGGGTGGGTACAGGATGAACTGTGTATCCTCCCTTGTCTTCTAATGTAGGGCATGCTTTTGTTTCTGTAGATTTTGCTATTTTTTCTCTTCATTTGGCGGGGGCTTCTTCTATTATGGGTGCTGTTAATTTTATTACTACTATTGTAAATATGCGAACTTTTGGAATGACAATGGAAAAAGTTCCATTGTTTGTGTGATCAGTGTTAGTTACTGCTGTTTTATTGTTGTTATCTTTACCAGTATTAGCTGGAGCTATTACTATATTATTAACAGATCGTAATTTTAATACTTCTTTTTTTGACCCCGCTGGGGGCGGGGATCCGGTATTGTTTCAACATTTN

>KY017764 Leptoneta_infuscata

CGTATAAATAATTTAAGATTTTGGTTGTTGCCACCTTCTTTATTTTTGTTAATTGTTTCTAGTATAGTAGAAATAGGGGTGGGAACTGGTTGGACTGTTTATCCCCCTTTAGCTTCTAATGTTGGTCATGCTTTTGTATCTGTAGATTTTGCTATTTTTTCTCTTCATTTGGCTGGAGCTTCTTCTATTATGGGGGCTATTAATTTTATTACTACTATTATGAATATGCGTACTTTTGGGATGAGAATGGAGAAGGTTCCATTATTTGTTTGGTCGGTATTAGTAACTGCTATTTTGTTATTATTGTCTTTACCTGTGTTAGCAGGTGCTATTACTATATTGCTAACAGATCGAAACTTTAATACTTCTTTTTTTGATCCTGCTGGAGGGGGAGATCCAGTTTTATTTCAACATTTN

>MT98795 Leptonetela_caucasica

CGAATAAATAATTTGAGATTTTGGTTATTGCCACCCTCTTTATTTTTATTGGTAGTTTCTGGTATGGTAGAGATAGGGGTGGGCACTGGTTGAACTATTTATCCTCCTTTGGCTGCTTATTTGGGGCATTCTTTTGTGTCAGTGGATTATGCTATTTTTTCTTTACATTTAGCTGGTGCTTCTTCTATTATGGGGGCTGTTAATTTTATTACTACTATTTTTAATATGCGTATAAATGGGATGAGAATGGAGAAGATGCCATTATTTGTTTGGTCAGTATTAATTACAGCAGTTTTATTATTATTGTCACTTCCAGTATTGGCTGGGGCCATTACTATGTTGCTAACAGATCGAAATTTTAATACTTCTTTTTTTGATCCGGCAGGTGGGGGAGATCCTGTTTTATTTCAGCATTTN

>MG573884 Leptonetela_paragamiani

CGAATGAATAATTTGAGTTTTTGATTGTTACCTCCATCTTTGTTTTTATTGGTAGTTTCTGGGATGGTAGAGGTAGGGGTTGGGACGGGATGAACTATTTATCCTCCTTTGGCTGCATATGTGGGACATTCTTTTGTGTCGGTGGATTATGCTATTTTTTCTTTGCATTTAGCGGGAGCGTCTTCTATTATAGGGGCTGTTAATTTTATTACGACTATTTTGAATATGCGCATGCTTGGAATAAGCATGGATAAAACACCTTTATTTGTTTGGTCTGTGTTGATTACAGCGGTTTTGTTGTTATTGTCTCTTCCTGTGCTCGCGGGGGCGATTACGATATTGTTAACGGATCGGAATTTTAATACTTCGTTTTTTGACCCCGCGGGGGGAGGGGATCCTGTTTTATTTCAACATCTN

>MG573899 Leptonetela_penevi

CGGATAAATAATTTGAGTTTTTGATTGTTGCCGCCGTCTTTGTTTTTGCTGGTAGTTTCTGGGATAGTGGAGGTAGGGGTTGGTACGGGGTGAACTATTTATCCTCCTTTGGCTGCATATGTGGGACATTCGTTTGTGTCGGTGGATTATGCTATTTTTTCTTTACATTTAGCGGGAGCGTCTTCTATTATAGGGGCTGTTAATTTTATTACGACTATTTTGAATATACGAATGTCTGGAATAAGTATGGATAAGACGCCTTTGTTTGTTTGATCAGTGTTGATTACGGCGGTACTGTTGTTGTTATCTCTTCCTGTATTAGCGGGGGCAATTACGATGTTGTTAACGGATCGGAATTTCAATACTTCATTTTTTGATCCTGCAGGGGGAGGGGATCCTGTTTTGTTTCAACATTTN

>MG573895 Leptonetela_chiosensis

CGGATAAATAATTTAAGTTTTTGGTTGTTGCCGCCATCTTTGTTTTTGTTGGTGGCTTCTGGGTTAGTGGAAATAGGGGTGGGGACTGGGTGGACTGTTTATCCGCCATTGGCTGCGTATTCTGGTCATTCTTTTGTGTCAGTAGATTATGCTATTTTTTCTTTACATTTAGCGGGGGCGTCTTCTATTATGGGGGCTGTTAATTTTATTACGACTATTTTGAATATGCGAACTAGTGGCATGAGAGTGGATAGGATGCCTTTATTTGTTTGGTCTGTGCTTATTACTGCGGTGTTATTATTATTATCCCTTCCAGTGTTAGCTGGGGCTATTACGATGTTATTGACGGATCGTAATTTTAATACTTCATTTTTTGATCCTGCTGGAGGGGGGGATCCTGTGTTGTTTCAGCATTTN

>MG573909 Leptonetela_thracia

CGTATAAATAATTTGAGTTTTTGATTGTTGCCTCCGTCTTTGTTTATATTGGTTGTTTCTGGTGTGGTGGGGGCAGGGGTGGGTACTGGATGGACGGTTTATCCTCCGTTGGCTGCGTATTCTGGGCATTCATTTGTGTCGGTGGATTATGCTATTTTTTCTTTACATTTGGCTGGAGCATCTTCTATTATGGGGGCTATCAATTTTATTACAACTGTTTTGAATATGCGAACGAGGGGGATAAGAATGGATAGGATGCCTTTATTTGTTTGGTCTGTTTTAATTACAGCGGTTTTGTTGTTGTTATCACTTCCAGTATTGGCGGGTGCTATTACTATATTGTTGACGGATCGAAATTTTAACACTTCATTCTTTGATCCTGCAGGAGGGGGGGATCCAGTGTTATTTCAACATTTN

>MT987955 Cataleptoneta_aesculapii

CGTATGAATAATTTAAGATTTTGGTTGTTGCCCCCTTCTTTATTTTTTTTGATTGTTTCTAGAATAGTAGAGATAGGAGTAGGGACGGGGTGGACGTTGTATCCTCCTCTGGCTAGTTATGTGGGGCATGGTTTTATTTCGGTGGATTATGCAATTTTTTCTCTTCATTTGGCTGGTGCTTCGTCTATTATGGGGGCTATTAATTTTATTACTACTATTATTAACATGCGAAGAAATGGGCTAGTAATGGAGAGGGTATCATTGTTTGTTTGGTCTGTTTTGATTACAGCGGTGTTGTTGTTGTTATCTTTACCTGTTTTGGCGGGAGCCATTACTATATTGTTGACTGATCGAAACTTTAATACTTCTTTTTTTGACCCTGCGGGGGGTGGTGATCCGATTTTGTTTCAACATTTN

>MG573904 Leptonetela_arvanitidisi

CGAATGAATAATTTAAGTTTTTGGTTATTACCTCCGTCGCTTTTTTTGTTGGTAGTCTCTAGACTAGTAGGGGTGGGGGCTGGCACGGGTTGAACTGTTTATCCCCCGTTAGCGGCATATTTAGGGCATTCGTTTGTGTCAGTAGATTATGCTATTTTCTCTTTACATTTAGCTGGGGCGTCTTCTATTATAGGGGCTGTCAATTTTATTACGACTATTTTAAATATGCGTACATGGGGTATGAGAATAGATAAGACTCCCTTGTTTGTTTGATCTGTGTTGATTACAGCGGTTTTACTGTTGTTATCCCTTCCCGTGTTAGCGGGAGCGATCACAATGTTATTGACTGATCGTAATTTTAATACTTCATTTTTTGATCCTGCGGGGGGGGGGGATCCTGTGTTATTTCAGCATTTN

>Cataleptoneta_sulfur_S2

CGTATGAATAATTTGAGTTTTTGATTGTTGCCGCCATCGTTGACTTTGTTAGTAATTTCAAGAATGGTGGAAATTGGGGCGGGGACTGGATGAACTGTGTATCCTCCTTTAGCTTCGTATATAGGGCATGCATTTTGTTCCGTAGATTTTGTTATTTTTTCCTTACATTTTGCTGGGGCGTCTTCGATTATAGGGGCTATTAATTTTATTACTACTATTATTAATATACGAAGAGGAGGGATAACAATAGAAAAGGTACCTTTGTTTGTTTGATCTGTTTTGGTTACTGCCGTATTGCTTTTATTGTCTTTACCTGTATTGGCTGGTGCTATTACTATATTGTTGACGGATCGAAACTTTAATACATCTTTTTTCGATCCTGCCGGGGGGGGGGATCCGGTTCTATTTCAGCATTTN

>Cataleptoneta_sulfur_T2

CGTATGAATAATTTGAGTTTTTGATTGTTGCCGCCATCGTTGATTTTGTTAGTAATTTCAAGAATGGTGGAAATTGGGGCGGGGACTGGATGAACTGTGTATCCTCCTTTAGCTTCGTATATAGGGCATGCATTTTGTTCCGTAGATTTTGTTATTTTTTCCTTACATTTTGCTGGGGCGTCTTCGATTATAGGGGCTATTAATTTTATTACTACTATTATTAATATACGAAGTGGAGGGATAACAATAGAAAAGGTACCTTTGTTTGTTTGATCTGTTTTGGTTACTGCCGTATTGCTTTTATTGTCTTTACCTGTATTGGCTGGTGCTATTACTATATTGTTGACGGATCGAAACTTTAATACATCTTTTTTCGACCCTGCCGGGGGGGGGGATCCGGTTCTATTTCAGCATTTN

>MT987968 Barusia sp. 3 HZ-2020

CGGATAAATAATTTGAGTTTTTGATTGTTACCCCCTTCGTTAAGACTTTTGCTTATTTCTGGGATGGTGGAAATAGGGGCAGGAACGGGATGAACTATTTATCCTCCTTTGGCTTCTTATGTGGGGCATCCTTTTGTGTCTGTGGATTTTGCTATCTTTTCTTTACATTTAGCAGGGGCGTCTTCTATTATAGGAGCTATTAATTTTATCACGACTATTTTAAATATACGGAGTGTAGGAATGACGATGGATAAGGTGCCTTTATTTGTTTGGTCGGTTTTAGTGACAGCGGTGTTGTTGTTGTTATCCTTGCCGGTATTAGCGGGGGCGGTCACAATATTATTGACGGATCGAAATTTTAATACGTCTTTTTTTGACCCTGCTGGAGGAGGAGATCCGGTGTTGTTTCAACATTTA

>MT987970 Barusia sp. 6 HZ-2020

CGGATGAATAATTTGAGTTTTTGATTGTTGCCACCGTCATTAAGATTATTAATTATTTCAGGAATAGTGGAGATAGGAGCGGGAACTGGGTGAACGATTTATCCGCCCTTGGCTTCTTATATTGGTCATCCGTTTGTTTCTGTGGATTTTGCTATTTTTTCTTTACATTTAGCGGGGGCGTCTTCTATTATAGGGGCTATTAATTTTATTACAACCGTGTTAAATATACGTAGAGTTGGGATGACTATAGATAAAGTGCCTTTATTTGTTTGGTCTGTTTTGGTCACTGCGGTGTTGTTGTTGTTGTCTTTACCTGTTTTGGCAGGTGCGATTACAATATTGTTGACGGATCGGAATTTTAATACATCATTTTTTGATCCTGCCGGGGGAGGGGATCCAGTGTTATTTCAACATTTA

>MT987971 Barusia sp. 7 HZ-2020

CGGATGAATAATTTGAGTTTTTGATTGTTGCCACCGTCATTAAGATTATTGATTATTTCGGGAATGGTGGAGATAGGAGCGGGAACTGGGTGAACGATTTATCCGCCCTTGGCTTCTTATATTGGTCATCCGTTTGTTTCTGTGGATTTTGCTATTTTTTCTTTACATTTAGCGGGGGCGTCTTCTATTATAGGGGCTATTAATTTTATTACAACCGTGTTAAATATACGTAGAGTTGGGATGACTATAGATAAAGTGCCTTTATTTGTTTGGTCTGTTTTGGTCACTGCGGTGTTGTTGTTATTATCTTTACCTGTTTTGGCAGGTGCGATTACAATATTGTTGACGGATCGGAATTTTAATACATCATTTTTTGATCCTGCAGGGGGAGGGGACCCAGTGTTATTTCAACATTTA

>JN378135 Calileptoneta_noyoana

CGTATAAATAATTTGAGTTTTTGATTACTTCCGCCTTCTTTATTATTGTTGTTATTTTCTAGAATAGTGGAGTTAGGGGCGGGTACTGGATGAACTGTTTATCCTCCGCTAGCATCTTATTTTGGGCATGCATTTGTTTCTGTTGATTTTGCTATTTTTTCTTTACACTTGGCAGGGGCTTCTTCTATTATAGGGGCGGTTAATTTTATTACTACTATTATTAATATACGTGTAAGAGGTATATCTTTTGATAAGGTATCTCTTTTTGTATGATCAGTTTTGATTACTGCGGTTTTGTTATTATTATCTCTTCCGGTATTAGCTGGTGCTATTACTATGTTGTTAACTGATCGTAATTTTAATACTTCTTTTTTTGATCCTTCAGGAGGAGGAGATCCTATTTTGTTTCAGCATTTG

>JN378140 Calileptoneta_helferi_voucher_904

CGTATGAATAATTTAAGATTTTGGTTGTTGCCGCCTTCTTTATTATTGTTGTTGTTTTCTAGCATGGTCGAGTTAGGGGCAGGTACCGGGTGAACTGTTTATCCCCCGCTTGCTTCTTATTTTGGGCATTCTTTTATTTCTGTGGATTTTGCTATTTTTTCTTTGCACTTAGCTGGGGCTTCTTCTATTATGGGGGCGGTAAACTTTATTTCTACTATTTTTAACATGCGAATGAAGGGCATGACTATAGATAGGGTCCCTCTTTTTGTGTGATCTGTTTTAATTACCGCGGTTTTGTTATTATTATCCCTTCCAGTGTTGGCTGGGGCCATTACTATGTTGTTGACTGATCGTAATTTTAATACGTCTTTTTTCGATCCTGCCGGTGGGGGGGACCCAGTTTTATTTCAGCATTTG

>JN378147 Calileptoneta_sp__undet__JL_2011_9040372

NNNNNNNNTAATTTGAGTTTTTGGTTGTTGCCACCCTCTTTGTTGTTGTTGTTATTTTCTAGTATGGTGGAGTTGGGGGCGGGTACTGGATGAACCATTTATCCTCCGCTTTCTTCGTATTTTGGTCATTCTTTTATTTCTGTGGATTTTGCTATTTTTTCTTTGCATTTAGCCGGAGCTTCTTCTATTATGGGAGCGGTGAATTTTATTACTACTATTGTAAATATACGTGTGGCTGGTATATCTATGGATAGGGTACCTCTTTTTGTTTGATCTGTTTTAATCACTGCAGTTTTGTTGTTATTATCGCTCCCGGTTTTGGCGGGAGCTATCACTATGTTATTAACTGATCGAAATTTTAATACTTCTTTTTTTGACCCCTCTGGGGGAGGGGATCCTATTTTGTTTCAGCATTTG

>JN378137 Calileptoneta_sp__undet__JL_2011_9040234

CGNATAAATAATTTAAGTTTTTGGTTGTTACCTCCCTCTCTATTGTTGTTGTTGTTTTCTAGAATGGTGGAATTAGGGGTGGGGACGGGGTGGACGGTATACCCCCCTCTTGCTTCTTATTTCGGACACTCTTTTATTTCTGTTGATTTTGCTATTTTTTCTTTACATTTGGCTGGAGCTTCTTCTATTATGGGGGCGGTTAATTTTATTTCTACTATTTTTAACATGCGAAGTGTGGGTATATCTATAGATAAAATTTCTCTCTTTGTATGATCTATACTAATTACGGCAATTTTATTGTTGTTGTCTCTTCCCGTATTAGCAGGTGCTATCACGATATTATTAACAGATCGAAATTTTAATACTTCTTTTTTTGACCCTGCAGGGGGTGGGGATCCTGTTTTATTTCAACATTTG

# Best Model

Starting SMS v1.8.1

~~~~~~~~~~~~~~~~~~~~~~~~~~~~~~~~~~~~~~~~~~~~~~~~~~~~~~~~~~~~

Input alignment	: align_rename.phy

Data type	: DNA

Number of taxa	: 30

Number of sites	: 417

Number of branches : 57

Criterion	: AIC

~~~~~~~~~~~~~~~~~~~~~~~~~~~~~~~~~~~~~~~~~~~~~~~~~~~~~~~~~~~~

Step 1 : Set a fixed topology	AIC=9443.86622

~~~~~~~~~~~~~~~~~~~~~~~~~~~~~~~~~~~~~~~~~~~~~~~~~~~~~~~~~~~~

Step 2 : Select the best decoration	AIC=9443.86622	decoration : '+G+I'

~~~~~~~~~~~~~~~~~~~~~~~~~~~~~~~~~~~~~~~~~~~~~~~~~~~~~~~~~~~~

Step 3 : Select the best matrix	AIC=0	matrix : 'GTR'

~~~~~~~~~~~~~~~~~~~~~~~~~~~~~~~~~~~~~~~~~~~~~~~~~~~~~~~~~~~~

Step 4 : Select the best final decoration	AIC=9443.86622	decoration : '+G+I'

~~~~~~~~~~~~~~~~~~~~~~~~~~~~~~~~~~~~~~~~~~~~~~~~~~~~~~~~~~~~

Selected model	: GTR +G+I

# ML Tree

(Cataleptoneta_sulfur_S2:0.01286695,Cataleptoneta_sulfur_T2:0.00000423,((MT987968-Barusia-sp-3-HZ-2020:0.59586165,(MT987970-Barusia-sp-6-HZ-2020:0.00000278,MT987971-Barusia-sp-7-HZ-2020:0.0321149)0.901:0.35525487)0.8545:0.46305789,(BSCRO41624-Cataleptoneta_lingulata:0.85311385,((BGE_00521_D05-Sulcia-26743015:0.20020177,(BGE_00513_D08-Sulcia-24761902:0.06660883,BGE_00521_E05-Sulcia-26743027:0.14830744)0.498:0.03482792)0.952:0.67389064,(((BGE_00512_E05-Barusia-insulana:0.10582342,(BGE_00510_B06-Barusia-korculana:0.45208227,BGE_00513_H08-Barusia-cf-maheni:0.3224973)0.671:0.15686024)0.975:0.67155602,(MT987955-Cataleptoneta_aesculapii:1.15899401,BSUIO106025-Cataleptoneta_detriticola:0.90519112)0.16900000000000004:0.00119835)0.5662499999999999:0.12272185,((KY017764-Leptoneta_infuscata:0.33021457,((MT607793-Leptoneta_paroculus:0.00000002,MW997984-Leptoneta_paroculus:0.00000002)0.954:0.26001038,(JN378155-Leptoneta_microphthalma:0.24255406,JN378156-Leptoneta-convexa-voucher-9040712:0.11347173)0.7989999999999999:0.26255291)0.8373333333333334:0.33255171)0.864:0.33445701,(((JN378135-Calileptoneta_noyoana:0.62758395,JN378147-Calileptoneta_sp__undet__JL_2011_9040372:0.4972349)0.754:0.18629732,(JN378140-Calileptoneta_helferi_voucher_904:0.28705869,JN378137-Calileptoneta_sp__undet__JL_2011_9040234:1.08772315)0.481:0.10419971)0.99:0.96202843,(KU318413-Protoleptoneta_bulgarica:1.14602035,(MT98795-Leptonetela_caucasica:0.28207029,((MG573904-Leptonetela_arvanitidisi:0.69187907,(MG573899-Leptonetela_penevi:0.17333867,MG573884-Leptonetela_paragamiani:0.23065187)0.771:0.18441415)0.8265:0.29074372,(MG573895-Leptonetela_chiosensis:0.54402249,MG573909-Leptonetela_thracia:0.60447018)0.45299999999999996:0.06553332)0.91225:0.41488107)0.9308:0.40819772)0.8555:0.09260244)0.8248:0.38967593)0.7388461538461538:0.08557974)0.636:0.26327974)0.5854:0.16209342)0.6677500000000001:0.13696936)1:0.82525577);

# Species Delimitations ASAP
